# Rearrangements of viral and human genomes at human papillomavirus integration events and their allele-specific impacts on cancer genome regulation

**DOI:** 10.1101/gr.279041.124

**Published:** 2025-04

**Authors:** Vanessa L. Porter, Michelle Ng, Kieran O'Neill, Signe MacLennan, Richard D. Corbett, Luka Culibrk, Zeid Hamadeh, Marissa Iden, Rachel Schmidt, Shirng-Wern Tsaih, Carolyn Nakisige, Martin Origa, Jackson Orem, Glenn Chang, Jeremy Fan, Ka Ming Nip, Vahid Akbari, Simon K. Chan, James Hopkins, Richard A. Moore, Eric Chuah, Karen L. Mungall, Andrew J. Mungall, Inanc Birol, Steven J.M. Jones, Janet S. Rader, Marco A. Marra

**Affiliations:** 1 Canada's Michael Smith Genome Sciences Centre, BC Cancer, Vancouver, British Columbia V5Z 4S6, Canada;; 2 Department of Medical Genetics, University of British Columbia, Vancouver, British Columbia V6T 1Z3, Canada;; 3 Michael Smith Laboratories, University of British Columbia, Vancouver, British Columbia V6T 1Z4, Canada;; 4 Bioinformatics Graduate Program, University of British Columbia, Vancouver, British Columbia V6T 1Z2, Canada;; 5 Cytogenomics Laboratory, Vancouver General Hospital, Vancouver, British Columbia V5Z 1N1, Canada;; 6 Department of Pathology and Laboratory Medicine, University of British Columbia, Vancouver, British Columbia V6T 1Z7, Canada;; 7 Department of Obstetrics and Gynecology, Medical College of Wisconsin, Milwaukee, Wisconsin 53226, USA;; 8 Medical College of Wisconsin Cancer Center, Milwaukee, Wisconsin 53226, USA;; 9 Uganda Cancer Institute, Kampala, Uganda;; 10 Genome Science and Technology Graduate Program, University of British Columbia, Vancouver, British Columbia V6T 1Z2, Canada

## Abstract

Human papillomavirus (HPV) integration has been implicated in transforming HPV infection into cancer. To resolve genome dysregulation associated with HPV integration, we performed Oxford Nanopore Technologies long-read sequencing on 72 cervical cancer genomes from a Ugandan data set that was previously characterized using short-read sequencing. We find recurrent structural rearrangement patterns at HPV integration events, which we categorize as del(etion)-like, dup(lication)-like, translocation, multi-breakpoint, or repeat region integrations. Integrations involving amplified HPV–human concatemers, particularly multi-breakpoint events, frequently harbor heterogeneous forms and copy numbers of the viral genome. Transcriptionally active integrants are characterized by unmethylated regions in both the viral and human genomes downstream from the viral transcription start site, resulting in HPV–human fusion transcripts. In contrast, integrants without evidence of expression lack consistent methylation patterns. Furthermore, whereas transcriptional dysregulation is limited to genes within 200 kb of an HPV integrant, dysregulation of the human epigenome in the form of allelic differentially methylated regions affects megabase expanses of the genome, irrespective of the integrant's transcriptional status. By elucidating the structural, epigenetic, and allele-specific impacts of HPV integration, we provide insight into the role of integrated HPV in cervical cancer.

Human papillomavirus (HPV), an 8 kilobase (kb), double-stranded, circular DNA virus, drives nearly all cervical cancers and a subset of head and neck cancers and anogenital cancers (de Sanjosé et al. 2018). Vaccination against high-risk HPV types has effectively reduced cervical cancer rates (Falcaro et al. 2021). However, cervical cancer incidence remains high in countries lacking vaccine availability (Bruni et al. 2016). The HPV genome normally exists as episomes in infected cells; however, during HPV-driven oncogenesis, the viral DNA often becomes integrated into the host cell genome (Huang et al. 2008). HPV integration is associated with changes in the sequence content of HPV, viral gene copy number, and epigenetic regulation of the viral genome (Groves et al. 2016; Warburton et al. 2018, 2021). Genomic and epigenomic changes can also occur in adjacent human genomic regions (Adey et al. 2013; Groves et al. 2021; Symer et al. 2022; Mima et al. 2023; Tian et al. 2023), and may have cancer-promoting consequences, as they often affect oncogenes within or near the site of HPV integration (Iden et al. 2021).

HPV integration is not a normal part of the HPV life cycle and occurs when double-stranded breaks in noncontiguous regions of the human genome are bridged using surrogate double-stranded DNA from the virus (Akagi et al. 2014). In cancers, HPV integration sites occur throughout the genome, but several loci are recurrently affected, including regions near *MYC*, *TP63*, *FHIT*, and *KLF5* (Hu et al. 2015; Bodelon et al. 2016; Gagliardi et al. 2020; Warburton et al. 2021; Symer et al. 2022; Fan et al. 2023). Different reports have indicated that integration may occur more frequently at regions with HPV microhomology, at fragile sites, or at sites that are actively transcribed (Hu et al. 2015; Warburton et al. 2021). Some of these apparent discrepancies may be attributed to differences in the sequencing approaches used (i.e., whole genome, HPV capture, RNA) (Fan et al. 2023). HPV integration sites are also associated with dense structural alterations, perhaps as a result of unstable HPV–human concatemers and intermediates that can form during replication and cause multiple HPV–human breakpoints across a genomic locus, which is referred to as an integration event (Kadaja et al. 2009; Akagi et al. 2023). The genomic consequences of HPV integration have thus far been difficult to study due to the limitations of short reads in capturing the complexity of human–HPV structures. However, recent advancements in long-read DNA sequencing technology have enhanced our ability to interpret repetitive sequences, structural alterations, and DNA methylation signals (Kovaka et al. 2023). Combined with enhanced haplotype phasing capabilities (Akbari et al. 2021), long-read sequencing may, therefore, be used to resolve complex HPV-integrated genomes and methylomes.

To investigate the structural changes resulting from HPV integration and their impacts on human and viral genome regulation, we used Oxford Nanopore Technologies (ONT) long-read sequencing to characterize HPV-positive cervical cancer tumor genomes from the HIV Tumor Molecular Characterization Project (HTMCP). We found 438 HPV–human integration breakpoints mapping to 129 integration events across 69 HPV-integrated cervical cancer samples, and characterized the genomic structures, methylation patterns, and transcriptional regulation associated with these loci. Our analysis revealed the extent of *cis*-linked genomic dysregulation resulting from HPV integration, including structural rearrangements and modulation of virus and host gene expression and epigenetic regulation.

## Results

### Long-read whole-genome sequencing of cervical tumors

We sequenced 72 cervical cancer samples from the HTMCP cohort using whole-genome Oxford Nanopore Technologies (ONT) long-read sequencing (Methods). The samples yielded an average of 102 gigabase pairs (Gb) of data (range 52.6–153 Gb; median coverage = 34×; Supplemental Fig. S1). We achieved long read lengths in our samples (median N50 = 17.5 kb; range 9.0–34.1 kb; Supplemental Fig. S1) that were of high read quality, as measured by the base error rate (median = 4.8%; range 2.2%–8.4%) and the proportion of artifactual chimeric DNA fusions (median = 5.0%; range 1.0%–12.4%). These data are supplemented by high-quality, short-read whole-genome and RNA sequencing (RNA-seq), as previously described (Gagliardi et al. 2020).

### Detection of HPV integration using long-read sequencing

**Figure 1.**
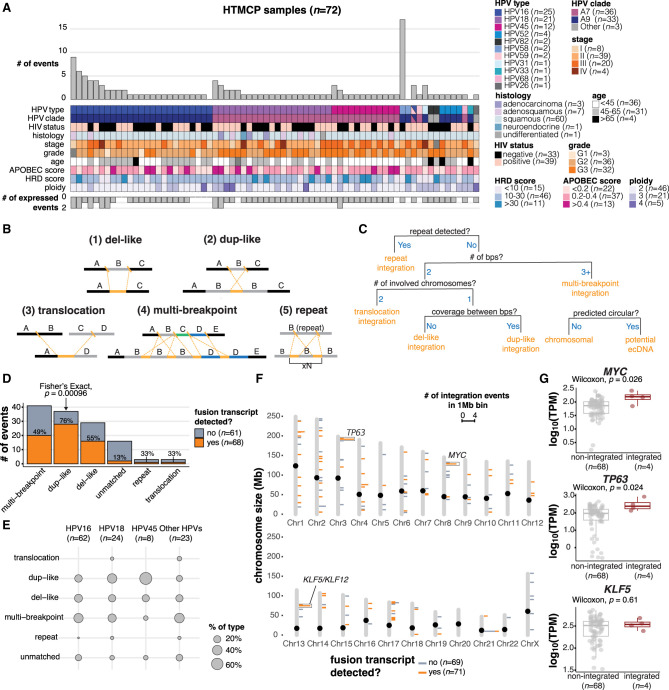
Detection and categorization of HPV integration events in cervical cancers. (*A*) The number of HPV integration events detected in the DNA (# of events) and detected in DNA and RNA (# of expressed events) across the HTMCP samples, as well as clinical and molecular characteristics. (*B*) Schematic illustrations of integration event categories. Orange segments indicate the insertion of HPV integrants. (*C*) Decision chart for categorizing HPV integration events (“bps” = breakpoints). (*D*) The frequency of the HPV integration categories across the samples. The percentage of events that produce an HPV–human fusion transcript is indicated for each integration type. (*E*) The percentage of events belonging to each integration category for HPV16, HPV18, HPV45, and all other HPV types. (*F*) The genomic locations of integration events across the cohort, colored by the transcriptional status of the event. Bins with 2+ integration events are highlighted with boxes. Notable cancer genes within bins recurrently affected by integration are indicated. (*G*) Gene expression differences between samples with HPV integration within 1 Mb of *MYC*, *TP63*, and *KLF5* compared to the remaining samples in the data set. Box plots represent the median and upper and lower quartiles of the distribution; whiskers represent the limits of the distribution (1.5 IQR below Q1 or 1.5 IQR above Q3). *P*-values were calculated using the Wilcoxon rank-sum test.

The molecular and clinical properties of the 72 samples are shown in Figure 1A (Supplemental Table S1). From a sequence perspective, we define HPV integration as the recombination of HPV with human DNA, which can be detected from chimeric reads that align to both the HPV and human genomes. We observed that HPV integration events involved at least two double-stranded human genome breaks and two double-stranded HPV genome breaks (Fig. 1B). Breakpoints were grouped into an “integration event” if either of the following conditions was met: (1) the HPV breakpoints co-occurred on one or more of the same reads, or (2) the breakpoints mapped within 500 kb of each other (The Cancer Genome Atlas Research Network 2017; Gagliardi et al. 2020; Methods). Applying these conditions, we detected 438 integration breakpoints belonging to 129 integration events across 69 samples with HPV integration (Fig. 1A; Supplemental Table S2). To determine whether an event was transcribed, we searched for HPV–human fusion transcripts occurring in the vicinity of the integration event using existing RNA-seq data (Methods). At most, two events per sample produced fusion transcripts, but even samples without a recurrent RNA fusion site near an integration event expressed HPV (Fig. 1A; Supplemental Table S2). In three samples with deep and evenly distributed read coverage across the HPV genome, we did not detect HPV integration breakpoints, indicative of highly amplified episomal HPV. One sample harbored three integration events from two different HPV types, HPV16 (two events) and HPV59 (one event), although only the HPV59 event was expressed (Supplemental Table S2).

Next, we compared the number and genomic locations of HPV integration breakpoints identified in our long-read sequencing data to previous short-read sequencing data (Gagliardi et al. 2020; Methods). Across the 69 HTMCP samples with HPV integration, 424 and 438 integration breakpoints were called in the short- and long-read sequencing data sets, respectively (Supplemental Tables S2 and S3). Integration breakpoint calls using short- and long reads were identical for 33 samples. Eighteen samples contained more calls in the long-read data, and 18 contained more calls in the short-read data (Supplemental Fig. S2A,B). Two samples where HPV integration was not detected with short reads contained one integration breakpoint each in the long-read data. One of these samples had even and deep read coverage indicative of episomal HPV, but an integration event was detected at a low variant allele frequency (VAF = 0.00025), suggesting that integration may have occurred subclonally (Supplemental Table S2). The second sample contained an integration site within a repetitive region of Chromosome 21p11 that was detected with long reads, but the region contained no short reads with adequate mapping quality.

Two samples had highly discordant integration breakpoint calls when detecting HPV–human junction sites, with an HPV58-integrated sample having 59 more calls in the long-read data (82 vs. 23) and an HPV16-integrated sample having 53 more calls in the short-read data (56 vs. 3; Supplemental Fig. S2A,B). We confirmed the overlap between integration breakpoint calls in each technology for these two samples. In the HPV58-integrated sample, 67/105 (64%) integration breakpoints called in either the long- or short-read data overlapped repetitive regions, and 30/59 (51%) breakpoints resided in repetitive regions in the HPV16-integrated sample, suggesting that read alignments may be confounded by repetitive sequences.

As expected, the number of integration breakpoint calls per sample was correlated between technologies (Supplemental Fig. S2C; Spearman's correlation, *R* = 0.78, *P* = 3.8 × 10^−15^). Both technologies detected similar proportions of breakpoint calls within CpG islands and exonic, intronic, and repetitive regions (Supplemental Fig. S2D). Long reads detected more integration breakpoints per sample, and discordance between calls made using long- and short-read technologies could be due to low-confidence mapping of short reads in repetitive regions.

### Structural alterations associated with HPV integration events

Based on the sequence alignment patterns of HPV-containing reads and the HPV integration breakpoints, we defined five categories of integration structures in the 69 integrated samples. These were (del)etion-like, (dup)lication-like, translocation, multi-breakpoint, and repeat region integrations, which we categorized using a custom workflow (Fig. 1B,C; Methods). Multi-breakpoint integrations were the most prevalent, comprising 41/129 (32%) of integration events in our data set (Fig. 1D; Supplemental Table S2). Next were dup-like (37/129) and del-like integrations (29/129), which differed by the presence or absence of the intervening human sequence between the two HPV breakpoints (Fig. 1B,C). We also investigated the potential of integration events existing as extrachromosomal circular DNA (ecDNA) using the events’ de novo assemblies (Methods). We identified eight events as potential ecDNAs, most of which (6/8) were categorized as dup-like integrations (Supplemental Table S4). Translocation (3/129), and repeat region (3/129) integrations were rare (Fig. 1D; Supplemental Table S2). The remainder of the events (16/129) were unmatched single breakpoints that we were unable to categorize (Supplemental Table S2). While multi-breakpoint events were the most prevalent, only 49% of these events produced HPV fusion transcripts, in contrast to 76% of dup-like events. In fact, dup-like events were more often expressed when compared to all other types of events (Fisher's exact test, *P* = 0.00096, Fig. 1D). The distributions of the integration categories also differed between HPV types (Fig. 1E).

Consistent with previous reports, we observed regions of recurrent HPV integration (>2 events within 1 Mb bins) in the genome (Hu et al. 2015; Bodelon et al. 2016; The Cancer Genome Atlas Research Network 2017; Gagliardi et al. 2020; Warburton et al. 2021; Symer et al. 2022; Fan et al. 2023), most of which contained events that produced fusion transcripts (Fig. 1F). The most frequently integrated loci in our data set were near *KLF5/KLF12* (13q22; Supplemental Fig. S3A), *MYC* (8q24; Supplemental Fig. S3B), and *TP63* (3q28; Supplemental Fig. S3C). All of these loci contained four integration events within 1 Mb of the respective gene and all integration events produced HPV–human fusion transcripts. All events in the 8q24 locus were multi-breakpoint events, whereas the 13q22 and 3q28 loci exclusively contained dup-like events (Supplemental Table S2). Another recurrently integrated locus was detected near the repeat-containing ribosomal gene *RNA28SN2* (21p11) in four samples (Fig. 1F). Three of the four events were not expressed, but we suspect this was due to a limited ability to map short RNA-seq reads in this low-complexity locus. The one expressed event was a multi-chromosomal event with a fusion transcript detected in a more mappable region overlapping *FAM83B* on Chromosome 6p12. We compared the expression of *MYC*, *TP63*, and *KLF5* in samples with and without HPV integration within 1 Mb of the respective genes (Fig. 1G; Supplemental Table S5) and observed that *MYC* and *TP63* expression was significantly increased in the integrated samples, whereas *KLF5* expression was not affected (Wilcoxon; *MYC P* = 0.026; *TP63 P* = 0.024; *KLF5 P* = 0.61; Fig. 1G).

### HPV structure and copy number heterogeneity within integration events

**Figure 2.**
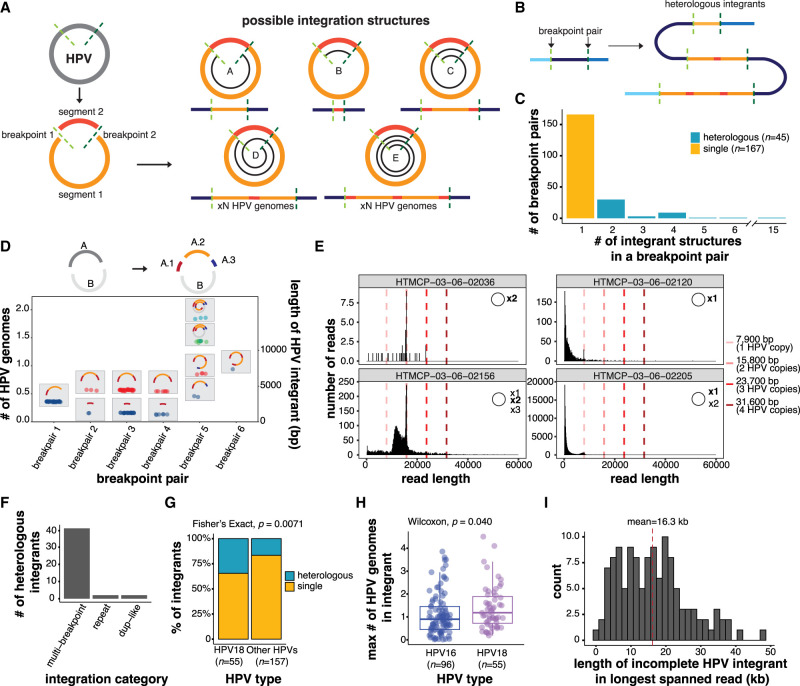
Heterologous structures of the HPV genome before and after integration. (*A*) Schematic of possible HPV integrant structures. The spirals in structures A–E show the portion(s) of the HPV genome that could be contained within an integrant. (*B*) Schematic showing how several heterologous integrants can exist between a single breakpoint pair, with the size of the integrant varying by *n* HPV copies. The colors correspond to the regions of the HPV genome as depicted in *A*. (*C*) The number of integrant structures between all identified breakpoint pairs within the cohort. Integrants with 2+ identified structures were classified as heterologous. (*D*) The sizes of the HPV integrants in a multi-breakpoint event with schematics depicting the various integrant structures detected. The HPV genome, in this case, was broken into two segments A and B, and the A segment was further broken into three segments (A.1, A.2, and A.3). These segments were variably rearranged into new structures across the breakpoint pairs. Each point represents the size of an HPV integrant contained on an individual read, which is then grouped together by color (e.g., blue, red, green, light blue) if they do not differ in size by more than 300 bp. Each color in a breakpoint pair thus represents one unique integrant structure, as indicated in the accompanying schematics. (*E*) The lengths of HPV-aligned reads in four predominantly episomal samples. The existence of HPV episomes and episome concatemers is supported by the accumulation of read counts in bin sizes corresponding to one or more HPV genome copies, as indicated by the dotted lines. (*F*) Frequency of heterologous integrants in the different integration categories. Only categories harboring heterologous integrants are shown. (*G*) The percentage of integrants from different HPV types that form single or heterologous structures. (*H*) The maximum size of the integrant structure in each breakpoint pair in HPV16 and HPV18 integrants, represented as the number of HPV genome copies. (*I*) Distribution showing the maximum number of HPV copies found in the longest spanning read for each incomplete integrant. The *x*-axis shows the length of the longest spanning read. Box plots represent the median and upper and lower quartiles of the distribution; whiskers represent the limits of the distribution (1.5 IQR below Q1 or 1.5 IQR above Q3). *P*-values were calculated using the Wilcoxon rank-sum test and the Fisher's exact test, as indicated in the figure.

We defined an HPV integrant as an uninterrupted segment of the integrated virus genome situated between two HPV–human breakpoints. Examples of possible integrant structures are shown in Figure 2A. An HPV breakpoint can be unambiguously linked to another HPV breakpoint when the entirety of the integrant is contained on a single read, and we refer to each unique pairing as a breakpoint pair (Supplemental Table S6). Using such reads, we found evidence for different configurations of viral genome segments—some of which were rearranged and/or concatemerized—between a single breakpoint pair (Fig. 2B). We refer to these as heterologous integrants, and hypothesize that they may arise as a result of unequal amplification of the HPV genome during the replication of HPV–human concatemers, akin to “heterocateny” described by Akagi et al. (2023) and the “onion-skin” structures described by Kadaja et al. (2009) (Fig. 2B). Heterologous integrants were found in 21% (45/212) of breakpoint pairs in our cohort (Fig. 2C; Supplemental Table S6), and up to 15 unique integrant configurations were observed within a single breakpoint pair (Fig. 2C). Figure 2D summarizes the different configurations of HPV integrants present in an example multi-breakpoint event. This sample harbored four connected breakpoints within the HPV genome, splitting it into four segments, which were variably rearranged across the integration event (Fig. 2D). We used read lengths and alignments to distinguish the HPV integrant sizes and structures (Methods), and detected various combinations—some of them periodic—of the four HPV segments between the different HPV–human breakpoint pairs within the event (Fig. 2D).

Heterogeneity in HPV genome structure has been reported in the episome prior to integration (Rossi et al. 2023). We therefore investigated HPV multimers, i.e., concatenated HPV genomes, and other HPV genome structural variants (SVs) in our four predominantly episomal samples (including one sample with subclonal integration, VAF = 0.00025). HPV genome multimers were found to be the dominant configuration in two samples, while the other two contained predominantly single-copy episomes, although we cannot exclude the possibility that shorter reads may bias these two samples toward detection of smaller configurations (Fig. 2E). Sample HTMCP-03-06-02156 showed an accumulation of read lengths between one and two HPV copies, indicating episomal structural variation, and we indeed found 23 viral SVs in this sample (Supplemental Fig. S4). We thus hypothesize that integrant rearrangement and copy number heterogeneity may partially originate within the episome prior to HPV integration.

Most heterologous integrants (91%) originated from multi-breakpoint events and were not detected in del-like or translocation integration events, compatible with the notion that amplified copy number is associated with their formation (Fig. 2F; Supplemental Table S6). HPV18 integrants were more frequently heterologous than other HPV types (35% vs. 21%; Fisher's exact test, *P* = 0.0071; Fig. 2G) and contained significantly more viral genome copies per integrant than HPV16 (Wilcoxon, *P* = 0.040; Fig. 2H).

We were unable to determine the complete integrant for 123 breakpoints because no reads linked the detected breakpoint to any other. For each of these incomplete integrants, we used the longest HPV sequence contained on a read to determine the minimum size of the integrant (Fig. 2I; Supplemental Table S6). Incomplete integrants often contained at least two HPV copies (mean = 2.1) and the longest incomplete HPV integrant was supported by a read that spanned 48 kb (Fig. 2I). In contrast, the largest complete integrant between a breakpoint pair was 37 kb. Thus, even with long-read sequencing, we did not achieve complete resolution of HPV integrant lengths in 123/335 (37%) of HPV breakpoints.

### Comparison of two-breakpoint events

**Figure 3.**
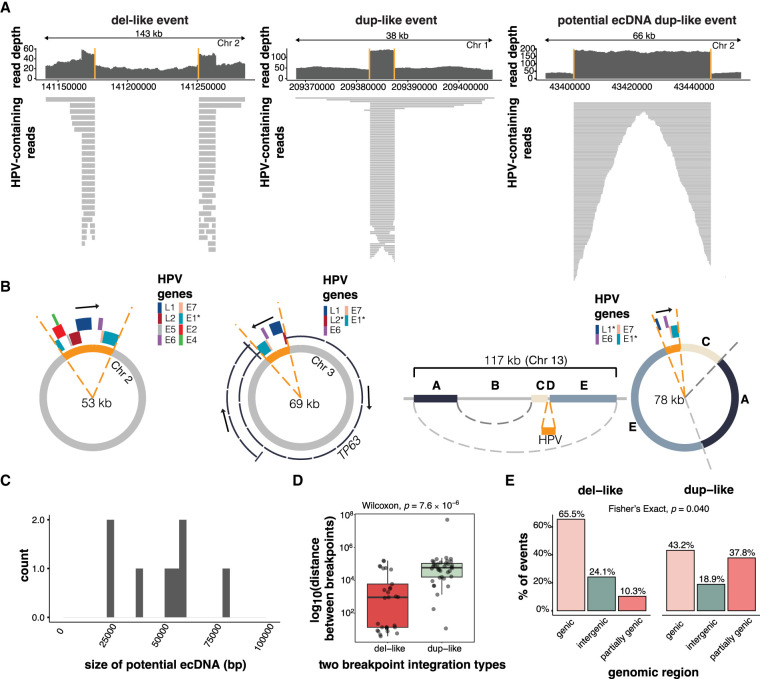
The characteristics of two-breakpoint events and potential ecDNAs. (*A*) Examples of read coverage patterns from a del-like event, a dup-like event, and a potential ecDNA dup-like event. Orange lines indicate the integration breakpoints. (*B*) Circular assemblies from three potential ecDNA integration events. The orange portions show the integrated HPV segment, including the viral genes. The direction of HPV gene transcription is shown by a black arrow. The right-most example depicts a complex event in which three nonadjacent human segments have been combined in the potential ecDNA. (*C*) The size distribution of potential HPV–human hybrid ecDNAs (*n* = 8). (*D*) The genomic distance between breakpoints in del-like versus dup-like events. Box plots represent the median and upper and lower quartiles of the distribution; whiskers represent the limits of the distribution (1.5 IQR below Q1 or 1.5 IQR above Q3). (*E*) The percentage of events occurring in genic (>90% within a gene), intergenic (<10% within a gene), and partially genic (10%–90% within a gene) regions, plotted by integration category. The *P*-value in *D* was calculated using a Wilcoxon ranked-sum test. The *P*-value in *E* was calculated using a Fisher's exact test.

Two-breakpoint integration events involving one chromosome comprised 66/129 (51%) of events in our cohort and occurred when one HPV integrant was inserted between two breaks in the human genome. These were associated with either del-like insertions or dup-like expansions (Fig. 1B), which were differentiated based on the alignment patterns of the HPV-containing chimeric reads. Most predicted ecDNAs (7/8) were also two-breakpoint events (Supplemental Table S4). Del-like events were characterized by a copy number loss between the integration breakpoints, in some cases accompanied by amplification of the regions flanking the integration (Fig. 3A). In dup-like events, including six potential ecDNAs, the region between the breakpoints was amplified; in predicted ecDNAs, read alignments were fully contained between and did not extend beyond the breakpoints (Fig. 3A).

We predict that candidate ecDNA integration events exist outside the chromosome in a self-contained circular structure, where the human region between the integration breakpoints is bridged by HPV (examples shown in Fig. 3B). The first example shows a near-complete copy of HPV18 connecting two breakpoints from an intergenic region on Chromosome 2 (Fig 3B, left). The second example shows a candidate ecDNA that contains a portion of *TP63* along with a portion of HPV45 (Fig. 3B, middle). The third example shows an atypical ecDNA integration event, in which the ecDNA contains part of HPV52 along with a rearranged intergenic segment of Chromosome 13 (Fig. 3B, right). The eight candidate ecDNAs had an average assembly size of 48.8 kb (range 22.4–78.0 kb; Fig 3C) and predominantly involved clade A7 HPV types (4 HPV18, 3 HPV45; Supplemental Table S4). We predicted no HPV16 ecDNAs.

The human–HPV breakpoints in dup-like and del-like integration events were distributed differently across the human genome. Firstly, there was greater spacing between the two breakpoints in dup-like events, with a median distance of 59.1 kb compared to 938 bp (Wilcoxon, *P* = 7.6 × 10^−6^; Fig. 3D; Supplemental Table S7). The dup-like events were also less likely to be completely contained within genic regions and more likely to have a breakpoint within the 3′ or 5′ end of a gene (Fisher's exact, *P* = 0.040; Fig. 3E; Supplemental Table S7).

### HPV integration is associated with full chromosome arm translocations

Translocation integrations, in which two breakpoints occurred on different chromosomes (Akagi et al. 2023), were rare in our study. There were only three instances, involving three different HPV types (HPV18, 52, and 31). The read alignments from the HPV52 translocation integration event are shown in Supplemental Figure S5. This event involved a focal amplification around the site of HPV integration and a segment of HPV containing *E6* and *E7* (Supplemental Fig. S5A). The Chromosome 8 breakpoint, although within a gene, occurred near the pericentromeric region and was adjacent to a one copy loss upstream of the breakpoint. The Chromosome 1 breakpoint occurred in a genic region on the p-arm and showed copy gain across a ∼70 Mb segment upstream of the integration breakpoint (Supplemental Fig. S5B). This indicates that the Chromosome 1 region was duplicated and recombined with the q-arm of Chromosome 8, the p-arm of Chromosome 8 was lost, and the HPV52 segment was focally amplified around the translocation junction (Supplemental Fig. S5C).

### Multi-breakpoint HPV integration events are structurally complex

**Figure 4.**
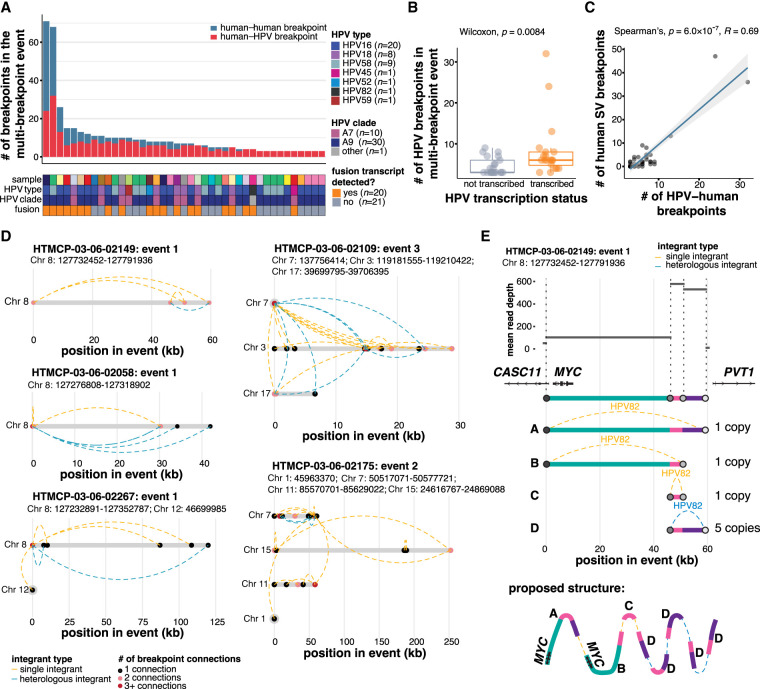
Complex structural variation is associated with multi-breakpoint integrations. (*A*) The number of human–HPV and human–human SV breakpoints across multi-breakpoint integration events, and the HPV type and clade in each. (*B*) The number of breakpoints per event in transcribed and nontranscribed multi-breakpoint events. Box plots represent the median and upper and lower quartiles of the distribution; whiskers represent the limits of the distribution (1.5 IQR below Q1 or 1.5 IQR above Q3). (*C*) Spearman's correlation between the number of HPV–human breakpoints and the number of human–human SV breakpoints in multi-breakpoint events. (*D*) Examples illustrating the connectivity between HPV breakpoint pairs in five multi-breakpoint events: three from the *MYC*-associated locus on Chromosome 8 and the two most rearranged multi-chromosomal events. Dots denote HPV breakpoints along the event, and dotted lines represent the HPV integrants that connect the breakpoints. The dots are colored according to the number of connections that converge at that position in the event. The integrants are colored according to whether single (orange) or heterologous (blue) integrant structures connect the breakpoints. (*E*) An example breaking down the copy number changes and the proposed structure of an event overlapping *MYC*. Each color represents a genomic segment on Chromosome 8 in between two human–HPV breakpoints. *P*-values in *B* and *C* were calculated using the Wilcoxon rank-sum test and Spearman's correlation test.

Multi-breakpoint events, comprising 41/129 (32%) of all events, were the most common type of event in our study. Figure 4A summarizes the number of HPV–human breakpoints per multi-breakpoint event, which ranged from 3 to 32 (Supplemental Table S8). On average, transcribed integration events contained more breakpoints per event than nontranscribed events (Wilcoxon, *P* = 0.0084; Fig. 4B). Integrations with higher numbers of HPV breakpoints were also associated with more human-only SVs overlapping the integration event (Spearman's correlation, *R* = 0.69, *P* = 6.0 × 10^−7^) indicating general instability within these regions (Fig. 4C; Supplemental Table S8).

We explored how HPV breakpoints were connected within multi-breakpoint events. Five examples are shown in Figure 4D, three of which originate from the recurrently integrated region on Chromosome 8 near *MYC* (Fig. 1F). The presence of heterologous integrants between breakpoint pairs is indicated, as is the degree of connectivity of each breakpoint (Fig. 4D). HTMCP-03-06-02058 and HTMCP-03-06-02267 both feature one highly connected breakpoint that pairs with all other breakpoints in the event. This contrasts with the HTMCP-03-06-02149 event, in which each breakpoint connects to two others. The two multi-chromosome events shown in Figure 4D were the two most rearranged events. The HTMCP-03-06-02109 event involved a heavily rearranged Chromosome 3, while Chromosomes 7 and 17 contained one and two breakpoints, respectively. The single breakpoint on Chromosome 7 is connected to almost every other breakpoint on the other two chromosomes. In contrast, HTMCP-03-06-02175 contained three highly connected breakpoints but none connected with all three other chromosomes.

To resolve the copy number patterns that can occur at multi-breakpoint events, we delved deeper into the structure of HTMCP-03-06-02149 event one (Fig. 4E). This event was of particular interest because it encompassed, but did not disrupt, the oncogene *MYC*. Read depth conversions were used to predict absolute copy number ratios for each segment bridged by an HPV breakpoint pair in the event. The three segments that were connected by nonheterologous integrants, including the region containing *MYC*, each showed a one copy increase above the baseline adjacent to the event (Fig. 4E). The segment between the heterologous integrant breakpoint pair showed a five-copy increase and this breakpoint pair was associated with heterologous integration, compatible with the notion that amplification may be involved in the generation of heterologous integrants (Fig. 4E). Thus, each of the five copies in the concatenated HPV–human amplification has altered HPV integrant structures. The resulting proposed structure, therefore, contained seven HPV integrants linking the human genome segments, including two uninterrupted copies of *MYC*, as illustrated in Figure 4E.

### HPV integration events show consistent orientation-dependent regulatory patterns

**Figure 5.**
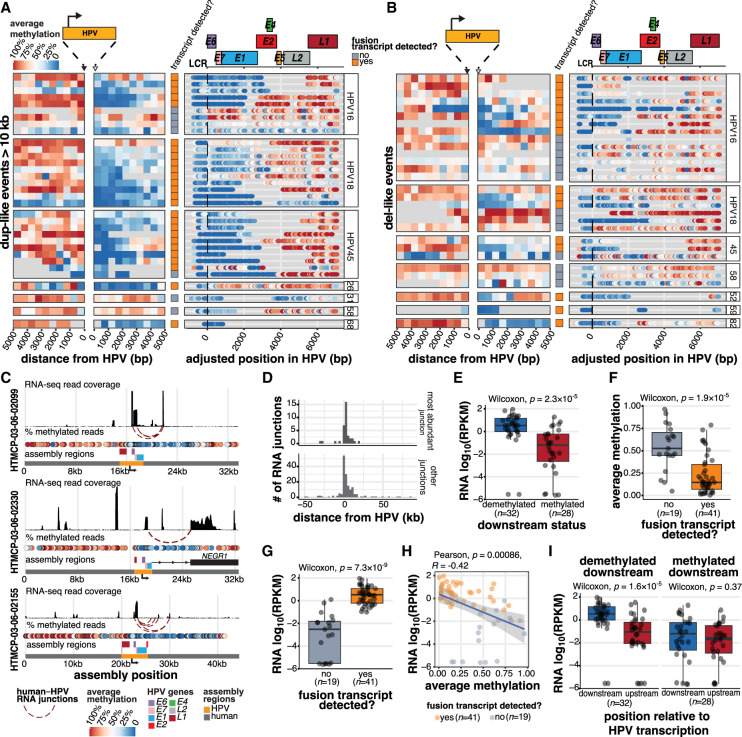
The production of HPV fusion transcripts correlates with distinct methylation patterns adjacent to and within the HPV integrant. (*A*,*B*) The proportion of HPV-containing reads showing methylation at positions within and adjacent to HPV integrants in (*A*) dup-like events (amplified region >10 kb) and (*B*) del-like events. The regions 5 kb upstream of and downstream from (relative to the direction of HPV transcription) are divided into 500 bp bins, and the average methylation frequency for all the CpGs within each bin is shown. Within HPV, the methylation of each CpG bin is shown as a colored dot. The transcriptional status is also indicated for each event (row). All the events are aligned to the start of the genic region (*E6* start) for each respective HPV type. The gene model for HPV16 is shown above for general reference. (*C*) The assemblies of three representative HPV integration events, including their human and HPV gene positions, RNA-seq coverage, and HPV–human fusion junctions. (*D*) The position of HPV–human RNA fusion points relative to the nearest DNA HPV breakpoint, oriented by the strand of the HPV integrant. The most abundantly expressed junctions (*n* = 36) are contrasted with all other identified junctions (*n* = 163). (*E*) The normalized expression (RPKM) within the 5 kb region downstream from the HPV integrant, stratified by the downstream methylation status. (*F*) The average downstream methylation (0 to 2500 bp), stratified by HPV transcription status. (*G*) The normalized expression in reads per kilobase per million (RPKM) within the 5 kb region downstream from the HPV integrant, stratified by the HPV transcription status. (*H*) Pearson's correlation between downstream methylation and expression in transcribed and nontranscribed events. (*I*) The difference in the expression (RPKM) upstream of and downstream from (±5 kb) the HPV integrant in events stratified by the downstream methylation status. In all cases, box plots represent the median and upper and lower quartiles of the distribution; whiskers represent the limits of the distribution (1.5 IQR below Q1 or 1.5 IQR above Q3). *P*-values were calculated using the Wilcoxon rank-sum test and Pearson's correlation, as indicated in the figures. The data points in *E–I* and the accompanying statistics are from the dup-like and del-like events shown in *A* and *B*.

ONT sequencing simultaneously yields DNA methylation information along with DNA sequence data (Simpson et al. 2017). We therefore investigated methylation patterns at HPV integration events, including within the integrated HPV genome and the flanking human regions, and related these to the transcriptional status of the event. Methylation of CpGs within HPV integrants and up to 5 kb on either side of the breakpoints is shown in Figure 5A and B for dup-like and del-like integration events (Supplemental Tables S9, S10). The integration events are oriented according to the direction of HPV transcription. In transcribed events, we observed that human regions upstream of HPV transcription were methylated, while human regions downstream from HPV transcription were unmethylated, and that the HPV late genes (*L1* and *L2*) were methylated, while the early genes (specifically *E6* and *E7*) were unmethylated after the long control region (LCR) up to the 3′ breakpoint (Fig. 5A,B). This pattern was especially pronounced in dup-like events (Fig. 5A). In contrast, events without evidence of HPV expression tended to lack consistent methylation patterns adjacent to HPV and contained partially methylated CpGs across the HPV genome (Fig. 5A,B). In all integrants, the LCR, a noncoding regulatory region that activates HPV early gene transcription (Maki et al. 1996), was invariably hypomethylated compared to the genic region of HPV (Fig. 5A,B).

We next investigated how the production of HPV fusion transcripts correlated with methylation patterns across HPV integration events. Integration often truncates or disrupts the HPV genome in such a way that the transcription termination signal for the early genes is lost (normally positioned after *E5*). We would thus expect transcripts produced from such integrants to read through downstream into the human genome, where transcription might terminate. We show the epigenetic and transcriptional landscapes of HPV integrants using the assemblies of three representative events (Fig. 5C). One event occurred in an intron of *NEGR1* upstream of its 3′ exon, and produced an *E7*^*NEGR1* fusion transcript containing the poly(A) tail of the human gene (Fig. 5C). However, the other two events did not overlap any genic elements on the same strand. We observed that, across all samples, HPV–human RNA fusions consistently occurred downstream from the nearest HPV integration breakpoint, especially when focusing on the most abundantly expressed fusion sites (Fig. 5D), and they occurred within the demethylated region adjacent to the HPV integrant (Fig 5C). In fact, events with a demethylated downstream region showed significantly higher normalized expression in the 5 kb bin downstream from HPV compared to events that were methylated (Wilcoxon, *P* = 2.3 × 10^−5^, Fig. 5E). Events that generated HPV fusion transcripts had lower downstream methylation (Wilcoxon, *P* = 1.9 × 10^−5^, Fig. 5F) and higher downstream expression (Wilcoxon, *P* = 7.3 × 10^−9^, Fig. 5G) than nontranscribed events, and expression and methylation in the downstream region were negatively correlated (Pearson's correlation, *R* = −0.42, *P* = 0.00086, Fig. 5H). However, the increased expression adjacent to the HPV integrant was unique to the downstream region: in events with a demethylated downstream region, there was a significant imbalance in expression between the upstream and downstream regions, relative to HPV, whereas no such difference was observed in the methylated events (Wilcoxon, *P* = 1.6 × 10^−5^, Fig. 5I).

The methylation patterns in the integrated HPV genomes contrasted with the four samples harboring episomal HPV (Supplemental Fig. S6A), in which viral sequences were hypomethylated compared to the integrated HPV genomes, particularly in the late genic region. The hypermethylation of select HPV genes, therefore, is a common feature of integrated HPV genomes in our cohort and was not seen in samples containing HPV episomes. In the three translocation events, HPV methylation did not follow the standard pattern and we suspect they instead are more influenced by the surrounding human methylation (Supplemental Fig. S6B).

### Allele-specific expression and methylation patterns in regions of HPV integration

**Figure 6.**
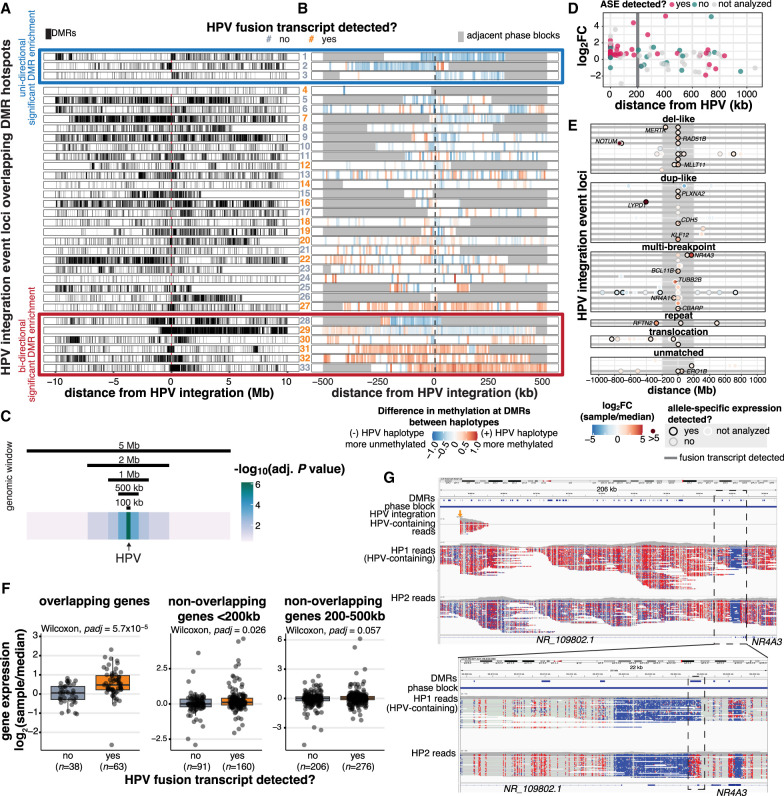
HPV integration is associated with dysregulation of the methylome and nearby genes on the integrated haplotype. (*A*) The distribution of DMRs across 10 Mb on either side of HPV integrations in the 33 events that overlapped a DMR hotspot. Each tick represents a DMR. Some event loci were situated <10 Mb from the end of the chromosome or from an unmappable region, resulting in a gap in DMRs before the end of the window (#7, 8, 12, 15, 19, 20, 23, 25, 26, and 29). (*B*) The direction of methylation changes in the HPV-containing haplotype with respect to the unintegrated haplotype within the phase block containing HPV integration. Adjacent phase blocks are shown as flanking gray bars. Events are ordered identically in *A* and *B*. The color of event numbers (center column) indicates the transcriptional status of the event. Events within the red (*bottom*) and blue (*top*) boxes show significant DMR enrichment (*P adj* < 0.05) either unidirectionally (blue box) or bidirectionally (red box) relative to the HPV integration event, as determined by a permutation test of the 500 kb bins flanking the event. (*C*) The significance of the association between HPV integration and high DMR density at all 147 HPV-integrated regions, using window sizes of 100,000 bp, 500,000 bp, 1,000,000 bp, 2,000,000 bp, and 5,000,000 bp around HPV. (*D*) The fold change and allele-specific expression (ASE) status of outlier genes (1.5 IQR below Q1 or 1.5 IQR above Q3) within 1 Mb of integration events. The log_2_ fold change (log_2_FC) of the integrated sample is relative to the median of the cohort. (*E*) The position of genes with outlier expression (1.5 IQR below Q1 or 1.5 IQR above Q3) relative to sites of HPV integration. Color indicates expression fold change in the integrated sample relative to the median of the cohort. The ASE status, integration event type, and transcriptional status of the event are also indicated. (*F*) The difference in gene expression fold change (integrated sample/median) at transcribed (yes) and nontranscribed (no) integration events. All box plots represent the median and upper and lower quartiles of the distribution; whiskers represent the limits of the distribution (1.5 IQR below Q1 or 1.5 IQR above Q3). Adjusted *P*-values in *F* were calculated using Benjamini–Hochberg-corrected Wilcoxon rank-sum tests. (*G*) Integrative Genomics Viewer snapshots showing wide (*top*) and zoomed (*bottom*) views of the haplotype-specific methylation changes around *NR4A3* and HPV integration in HTMCP-03-06-02428 (#31), with reads separated into the two haplotypes (HP1 and HP2). The sample's DMRs, phase blocks, and HPV integration breakpoints are also indicated in the top three tracks. Reads are colored by CpG methylation status, with red indicating methylated and blue indicating unmethylated.

HPV integrates into only one allele, leaving the other unaffected (Karimzadeh et al. 2023). We leveraged this to explore how HPV integration may have allele-specific impacts on the epigenome and human gene targets on a broader scale. We phased reads into haplotypes and determined the allele from which the HPV-containing reads originated, and then identified differentially methylated regions (DMRs) between the two haplotypes across the tumor genome (Methods). DMR density was compared to a null distribution across the human chromosomes to identify regions with significantly dense clusters of DMRs, referred to as DMR hotspots (Methods). HPV integration sites were then intersected with the DMR hotspots to identify events that potentially affected DNA methylation in an allele-specific manner. Across the samples in our data set, 33 integration event loci overlapped autosomal DMR hotspots (Fig. 6A; Supplemental Fig. S7; Supplemental Table S11). HPV-overlapping DMR hotspots had a median size of 6.1 megabases (Mb; range 1.8–26 Mb) compared to the overall genome-wide median of 3.9 Mb (range 18 kb–56 Mb) and mostly overlapped multi-breakpoint events (Supplemental Fig. S8A,B).

There was variation in DMR distribution at the integration-associated hotspots, in regards to their position relative to HPV and in size and density (Fig. 6A). We therefore tested, in each integration region, if high DMR density was unique to the sample harboring HPV integration by comparing the DMR density in the adjacent 500 kb region to 1000 randomly selected regions (Methods; Supplemental Fig. S9). Three and six events were significantly enriched for DMRs on one (i.e., unidirectional) and both sides of HPV (i.e., bidirectional), respectively, in the integrated sample (*P adj* < 0.05; Benjamini–Hochberg corrected permutation test; Fig. 6A; Supplemental Figs. S10, S11). Four of the six bidirectional DMR-enriched events produced fusion transcripts (Fig. 6A; Supplemental Table S11), whereas none of the unidirectional DMR-enriched events had HPV–human transcripts detected (Fig. 6A; Supplemental Table S11). The density of DMRs appeared focal around unidirectional DMR-enriched integration events, affecting <1 Mb adjacent to the HPV integrant, while bidirectional enrichment was more distributed, spanning 1–8 Mb from the HPV integrant (Fig. 6A; Supplemental Fig. S12). DMR densities in the remaining 24 events were not statistically different from the same regions in other cervical cancer samples, suggesting the high density of DMRs in these regions may occur independently of HPV integration (Fig. 6A).

We also investigated the direction of DNA methylation changes on the integrated haplotype. All three unidirectional DMR-enriched integration events (events 1–3) were hypomethylated on the HPV-integrated allele, while bidirectional DMR-enriched events showed both hypermethylation (events 31–33) and hypomethylation (events 28–29) (Fig. 6B; Supplemental Table S11). We note that, though methylation changes generally occurred in a uniform direction over a broad region of the human genome in the significantly DMR-enriched events, transcribed events still followed the pattern described in Figure 5 in the CpGs adjacent to the HPV–human breakpoints, suggesting that the local and distal methylation changes may be independent phenomena (Supplemental Fig. S11B).

We also analyzed the association between HPV integration and DMR density across our cohort and observed that regions surrounding HPV integration exhibited a significantly higher density of DMRs compared to 10,000 randomly selected control regions with comparable GC-content (Supplemental Fig. S13A; Methods). This effect was more pronounced closer to the integration event, but was significant up to a 2 Mb (±1 Mb) window (Wilcoxon, *P adj* < 0.05, Fig. 6C; Supplemental Fig. S13B), indicating that HPV's impact on DNA methylation was strongest near the integration event and gradually diminished around ±1 Mb away from the event (Fig. 6C).

We next searched for nearby genes whose transcription was potentially affected by HPV integration. We limited our analysis to loci within 1 Mb of HPV integration sites, which is approximately the window within which DMR enrichment was observed. We identified 2066 genes near HPV integrants across 68 samples. Of these, 101 genes had outlier expression levels in 31 integrated samples and 43 integration event loci (Fig. 6D,E; Supplemental Table S12; Supplemental Fig. S14). We examined these genes for allele-specific expression (ASE), which would be consistent with a *cis*-acting element (e.g., HPV integration) altering gene expression on one allele. Of the expression outliers that we could test for ASE, 69% (44/64) showed ASE (Methods; Supplemental Table S12). Fifty percent of the outlier genes (50/101) were within 200 kb of the HPV integration site (in 26 samples and 31 events), and these tended to be overexpressed (48/50 genes; 96%) and have ASE (32/38 tested genes; 84%). Outlier genes between 200 kb and 1 Mb away (51/101) contained significantly fewer that were overexpressed (30/51 genes; 59%; Fisher's exact test, *P* = 7.4 × 10^−6^), indicating that HPV integration may have greater influence on gene expression within 200 kb of the event.

When comparing gene expression in the integrated sample versus the rest of the cohort, genes overlapping expressed HPV integration events (i.e., HPV–human fusion transcripts were detected) showed significantly higher upregulation than those overlapping nonexpressed HPV integration events (Fig. 6F). We also observed greater upregulation of nonoverlapping genes within 200 kb of expressed HPV integration events (Fig. 6F). Genes 200–500 kb away from HPV integration showed no significant difference between expressed and nonexpressed events (Fig. 6F).

The nuclear receptor genes *NR4A1* and *NR4A3* (Wenzl et al. 2015) were the genes most highly overexpressed in an allele-specific manner from HPV-containing haplotypes (Supplemental Figs. 14, 15A,B). *NR4A1* (Chromosome 12) and *NR4A3* (Chromosome 9) were detected from two independent integration events in two different samples. Nonintegrated samples showed ASE of *NR4A1* and *NR4A3* (11 and 2, respectively), but in both cases, the major allele frequency observed in the integrated sample was much higher than the majority of samples (Supplemental Fig. 15A,B). Two outlier genes, *NR4A3* and *CBARP*, overlapped significant DMR-enriched events (event 31 and 29; Fig. 6A,B; Supplemental Table S12), and the remaining seven significant DMR-enriched events were not associated with changes in gene expression within 200 kb, suggesting that allelic methylation and gene expression changes may be two independent consequences of HPV integration.

To determine how HPV may contribute to the upregulation of oncogenes, we explored the DNA methylation landscape around HPV integration in the *NR4A3*-activated sample. This event was a multi-breakpoint event, with one breakpoint upstream of *NR4A3* on Chromosome 9 that connected to a Chromosome 14 locus harboring 12 breakpoints (Supplemental Fig. S16). The Chromosome 9 locus overlapped a DMR hotspot that was hypermethylated on the integrated allele (Fig. 6G, top). However, several DMRs near the *NR4A3* promoter were unmethylated on the integrated allele, including an 821 bp DMR in exon two of *NR4A3* at the 3′ end of an unmethylated promoter element (Fig. 6G, bottom), distinguishing it from other cervical cancer samples we profiled (Supplemental Fig. S17, bottom). The broad methylation on the HPV-containing allele was also unique to the integrated sample (Supplemental Fig. S17, top). We thus observed, in the *NR4A3*-activated sample, both HPV-associated large-scale allelic methylation as well as focal demethylation of potential *NR4A3* regulatory elements, and high expression of *NR4A3* transcripts from the integrated allele.

## Discussion

HPV integration plays a crucial role in the development of HPV-driven cancers and is associated with complex dysregulation of the host genome, the nature and effects of which remain incompletely understood. Our study aimed to provide a detailed overview of genomic structures and epigenomic and transcriptional changes associated with HPV integration events in cervical tumors harboring various HPV types. We used ONT long-read sequencing to generate reads that could span the distances between HPV–human breakpoints, thereby enabling the reconstruction of complex events. Based on the HPV-containing reads, we categorized integration events, and related HPV-associated transcription and structural alterations to local (±5 kb) and distal (±1 Mb) regulatory changes in the tumor.

The simplest HPV integration structures we observed involved two virus–human breakpoints. Categories of two-breakpoint events included dup-like and del-like, and, less frequently, translocation events. Dup-like events, and to a lesser extent, del-like events, showed consistent methylation patterns on the HPV integrant and adjacent human DNA, with methylation in the human region upstream of the first breakpoint persisting along HPV sequences until the LCR, where a hypomethylated state began and extended through the second breakpoint into the downstream human DNA. A demethylated downstream region was associated with the production of HPV–human fusion transcripts, which in some cases contained part of a human gene. De novo assembly indicated a circular topology for some events, which were categorized as putative ecDNAs. Some HPV–human chimeric candidate ecDNAs contained no human coding sequences, consistent with hypotheses that co-amplified HPV–human regions can act as cellular super-enhancers (Warburton et al. 2018) and that ecDNAs can act as mobile enhancer elements (Zhu et al. 2021).

In 21% of HPV integrants, we observed different rearrangements and copy numbers of the viral genome between the same two HPV–human breakpoints. The phenomenon of heterologous integration was first suggested by Kadaja et al. (2009), who showed that, upon integration, re-replication of the HPV genome generates intermediate structures whose resolution by DNA damage repair machinery leaves heterologous structures. In our cohort, heterologous integrants were only detected in regions with amplification between the breakpoints. This supports Kadaja et al.’s observations and potentially extends them by raising the possibility that the heterologous integrants may represent different copies within an amplification rather than different cell populations in a heterogeneous tumor, although we note that our bulk sequencing approach has limited ability to distinguish between these two possibilities.

Of the integration types, multi-breakpoint events were the most prevalent and complex. These events contained 3 to 33 HPV–human breakpoints across one or more loci, and were associated with amplified virus–human concatemers and other SVs in the tumor genome, akin to “heterocateny” described by Akagi et al. (2023). We reconstructed the two most complex multi-breakpoint integration events in our data set: One contained a single breakpoint that connected to most other breakpoints in the event, while the other involved three highly connected breakpoints that joined different regions of the event, suggesting different mechanisms. The single highly connected breakpoint suggests that the event arose at a single point in time within a highly fragmented region, which was reassembled using multiple HPV integrants to link the fragments, akin to a local chromothripsis event (Li et al. 2020). In contrast, the event with multiple highly connected segments is compatible with the notion that the integration gained complexity during the tumor's evolution (Akagi et al. 2023). When new double-stranded breaks occur, HPV integrants scattered across the genome may enable recombination between these regions through shared homology.

HPV integration has been reported to affect nearby gene expression, both through the production of viral–human fusion transcripts and by introducing and amplifying enhancers that can affect host genes (Adey et al. 2013; Brant et al. 2019; Gagliardi et al. 2020; Groves et al. 2021; Fan et al. 2023; Liu et al. 2023; Tian et al. 2023; Singh et al. 2024). We confirmed local and distal changes to gene regulation and extended these observations into the methylome. Adjacent to HPV integrants, we uncovered a novel association between the transcriptional status of the event and its downstream methylation state. High expression levels downstream from integrants overlapped demethylated regions on the human genome, which we hypothesize may play a role in the termination of HPV–human fusion transcripts. We also identified allelic changes in methylation and gene expression. HPV-integrated regions were associated with allelic DMRs up to ±1 Mb away from the integration event, and in nine events, we could specifically associate DMR density with the presence of HPV. Both transcribed and nontranscribed events were associated with DMR-enriched regions. Meanwhile, gene expression changes tended to occur within 200 kb of a transcribed event. We hypothesize that the local and distal effects of HPV integration on the methylome may occur through independent mechanisms. Longer-range methylation changes on the HPV-integrated allele could arise as a host defense mechanism in response to viral integration, as reported for other viruses and endogenous retroviruses (Jähner and Jaenisch 1985; Blazkova et al. 2009; Watanabe et al. 2015).

The methylation patterns within and adjacent to HPV integrants indicate that certain epigenetic states in the viral and host genomes are necessary to link the two and perhaps enable the production of HPV–human fusion transcripts. In contrast, the unintegrated HPV episomes in our study were comparatively hypomethylated, most notably in the late genic region. It has long been reported that the rate of HPV integration increases with cervical dysplasia severity (Cheung et al. 2008; Vinokurova et al. 2008). This is consistent with clinical studies reporting a positive correlation between HPV genome methylation and cervical precancer severity (Noyez and van de Wal 1989; Clarke et al. 2012, 2018; Wentzensen et al. 2012; Mirabello et al. 2013, 2015; Vasiljević et al. 2014; Liu et al. 2017; Bowden et al. 2019). Methylation of the viral late genes (*L1/L2*) in particular exhibited high diagnostic sensitivity for detecting high-grade neoplasms in HPV16-positive women (Bowden et al. 2019), and we speculate this may be indicative of viral integration as infection persists and progresses into neoplasia.

In our cohort, the *MYC* locus on Chromosome 8 (Hu et al. 2015; Bodelon et al. 2016; Gagliardi et al. 2020; Symer et al. 2022) harbored recurrent multi-breakpoint events, which were associated with increased *MYC* expression in the integrated samples. Chromosome loops connecting the integration region to *MYC* in a haplotype-specific manner have been described in HeLa cells (Adey et al. 2013), providing a mechanism by which *MYC* upregulation may be achieved. Recurrent dup-like integration events on Chromosome 3 within *TP63* (Bodelon et al. 2016; Liu et al. 2016; Gagliardi et al. 2020; Kamal et al. 2021) were also observed in our data set, and were associated with elevated *TP63* expression. A mechanism has yet to be described, but three of the four integrants were transcribed on the opposite strand, suggesting an orientation-independent mechanism such as enhancer activation. We also observed HPV-associated allele-specific activation of *NR4A3*, which was situated 170 kb downstream from an HPV breakpoint belonging to a multi breakpoint event spanning two chromosomes. In salivary gland acinic cell carcinoma, the cancer is driven by translocations that juxtapose super-enhancers from other chromosomes upstream of *NR4A3* (Haller et al. 2019a,b). We speculate that HPV-driven activation of *NR4A3* may occur through a similar mechanism. The ASE of *NR4A3* and its distance from the HPV breakpoint also suggest a physical interaction between enhancer elements involved in the HPV integration event and *NR4A3.* These enhancers may come from the HPV LCR or from the chromosome segments involved in the multi-breakpoint event. Further studies are required to determine how HPV integration leads to the activation of certain genes while driving megabase-scale hypermethylation on affected haplotypes.

Although we have explored new aspects of HPV integration biology, our study has several limitations. First, we did not examine how variations in HPV integrant sequences, whether from sublineages of HPV types or somatic mutations, might influence the regulation of HPV integration events and their effects on the surrounding genome. Second, our methods for reconstructing HPV integration events used two conditions to group HPV breakpoints (Methods), the second of which assumes that closely juxtaposed breakpoints belong to the same event in the absence of reads spanning the breakpoints. For two-breakpoint events, we assume that each breakpoint represents opposite ends of the integrant, and from this, we infer orientation and the direction of transcription. However, for multi-breakpoint events, we do not assume any specific linkages or orientations unless supported by the reads. Third, we do not provide orthogonal experimental evidence of predicted ecDNA events and rely on the topology of the supporting reads. However, several groups have reported circular structures at HPV integration events and validated these results using specialized techniques (Akagi et al. 2023; Tian et al. 2023). We note that our informatics methods are limited by read lengths, so larger ecDNAs are likely missed by our current approach, which relies on reads fully spanning the event. Read lengths remain a general limitation of our study, particularly as relates to resolving heterologous integrants and multi-breakpoint events. Lastly, we used bulk long-read WGS in this study, making it difficult to distinguish whether observations exist within individual cells or across different cell populations in the tumors. To confidently discern these two possibilities, a single-cell approach would be required.

Overall, our study leveraged long-read sequencing to identify the genomic and epigenetic impacts of HPV integration in cervical cancer. Recurrent integration-related structural rearrangements were observed, which had varied effects on the viral and human genomes. Multi-breakpoint events tended to be structurally complex and harbored heterologous configurations of the HPV genome linking its integration breakpoints. In dup-like and del-like integrations, demethylation downstream from the viral LCR was associated with the production of HPV–human fusion transcripts. We also observed widespread methylation and expression changes in *cis* with HPV integration, both proximal and distal to the integration site. The methylome in particular showed allelic differences megabases away from HPV integration. Our findings illustrate the multiomic dysregulation associated with HPV integration in cervical cancer and how this may result in oncogenic functions.

## Methods

### Ethics approval and consent to participate

The HTMCP patient cohort design was approved by the Fred Hutchinson Cancer Research Center Institutional Review Board (7662) and complied with ethical regulation; patient accrual received institutional and governmental approval, and informed consent was obtained from all patients (Gagliardi et al. 2020). The molecular characterization performed in this study had approval from the BC Cancer Research Ethics Board (UBC BC Cancer REB H19-03010) and the Medical College of Wisconsin Institutional Review Board.

### Sample selection

Our sample selection balanced HPV types, homologous recombination deficiency (HRD) scores (which may impact SV generation) (Telli et al. 2016), and HPV integration statuses, to what was previously identified across the whole cohort using short-read sequencing (Gagliardi et al. 2020). Our final data set included 72 samples from the HTMCP (Gagliardi et al. 2020), which consisted of 25 HPV16 tumors, 21 HPV18 tumors, 12 HPV45 tumors, and 14 tumors with less common HPV types, approximately reflecting the proportions of HPV types present in the larger HTMCP cohort. Visual inspection of the HPV genome alignments was performed to confirm the HPV-containing reads were of high quality, both in mapping quality and in length and contiguity. Three samples that were initially sequenced were excluded in the final data set due to an insufficient number of HPV-aligning reads, ambiguous or low-quality alignments, or highly fragmented and uninterpretable reads across the HPV genome due to low N50s and high chimeric rates in the sample. None of these samples had HPV integration detected by our workflow, but their low-quality alignments disqualified them from accurately representing episomal HPV.

### ONT library construction and whole-genome sequencing

HTMCP samples either had sufficient DNA left over from an earlier extraction (Gagliardi et al. 2020) or required fresh extraction. For archival (frozen) tissues, nucleic acids were extracted using bead-based or column purification methods. Blue Pippin size selection (Sage Science) was performed on 5 µg DNA to deplete shorter DNA molecules (<15 kb) from the final library to achieve 2 µg of final input DNA at a concentration of 42 ng/µL. Blue Pippin was not performed on samples with insufficient DNA yields. Forty-one samples were sequenced on R9.4 flow cells and 31 samples used R10.4.1 flow cells, depending on when they were sequenced (Supplemental Table S13). The ONT ligation-based library preparation kit (SQK-LSK110 for R9 samples and SQK-LSK114 for R10 flow cells) was implemented on a NIMBUS liquid handling robot (Hamilton), followed by the whole genome PCR-free library construction for ONT sequencing, as described (Dixon et al. 2023). The libraries were loaded onto quality-controlled flow cells exhibiting >5000 active pores within 5 days of construction to preserve the pore-targeting adapter. The library recovery after ligation and bead purifications was expected to be >40% of input (i.e., >800 ng). For yields 300–500 ng, the entire library was loaded onto a PromethION flow cell. For libraries with >500 ng yield, two-thirds of the library was loaded initially followed by a nuclease (DNase I) flush of the flow cell to restore the activity of the pores that had become clogged with DNA. The remaining one-third of the library was then loaded onto the restored flow cell for a maximum total of 750 ng. PromethION flow cells were typically run for 72 h for maximal sequencing yield.

### ONT primary data analysis

Raw signal from the PromethION sequencer was basecalled using ONT's Guppy 5 with the “super-accurate” model for the 41 R9 samples, and Dorado (v. 0.6.1) (https://github.com/nanoporetech/dorado) for the 31 R10 samples (Supplemental Table S13). These sequence data were aligned using minimap (v. 2.15) (Li 2018) to a custom reference containing GRCh38 with no ALT contigs and 17 HPV types from the HTMCP cohort, downloaded from the PaVE database (https://pave.niaid.nih.gov/) (Van Doorslaer et al. 2017). Subsequent primary analysis was carried out via a NextFlow workflow (Supplemental Table S13). SVs were called from the aligned BAM using Sniffles (v. 1.0.12b and v. 2.0.7) (Sedlazeck et al. 2018; Smolka et al. 2024) and CuteSV (v. 1.0.12) (Jiang et al. 2020); however, downstream HPV integration analyses used custom SV detection parameters using Sniffles, as described later (v. 1.0.12) (Sedlazeck et al. 2018). Small variants were called using Clair3 (v. 0.1-r8) (Luo et al.) and phased using WhatsHap (v. 1.0) (Martin et al. 2016), with phase blocks retained for later analyses. For the 41 samples run on R9 flow cells, DNA methylation (5mC) was called at the read level using Nanopolish (v. 0.13.2) (Simpson 2018) and phased into haplotypes using NanoMethPhase (v. 1.0) (Akbari et al. 2021). For the 31 samples run on R10 flow cells, methylation calls were extracted from the BAM file using modbam2bed (v. 0.9.1; https://github.com/epi2me-labs/modbam2bed) and phasing information from WhatsHap (v. 1.0) (Martin et al. 2016) was added as an additional tag on the BAM (Supplemental Table S13). Statistical testing for DMRs was performed using DSS (v. 2.47.1) (Feng and Wu 2019) in both workflows. No batch effects were observed when comparing the methylation frequencies at the 100 most variable CpG islands, downloaded from the UCSC Genome Browser (https://genome.ucsc.edu) CpG islands (Supplemental Fig. S18).

### Identifying HPV integration breakpoints, integration events, and integration event loci

Sniffles (v. 1.0.12) (Sedlazeck et al. 2018) was used to call translocations on the ONT long-read WGS using the following specifications to maximize the accuracy for detected HPV integration breakpoints: max_distance = 50, max_num_splits = −1, report_BND, num_reads_report = −1, min_support = 5, and min_seq_size = 500. A minimum of five consensus reads was required to detect an HPV integration breakpoint. An R script (v. R 4.0; R Core Team 2020) was developed that then iteratively grouped HPV breakpoints together if one or more reads overlapped between the breakpoints as indicated in the VCF. A distance threshold of 500 kb was also implemented using BEDTools (v. 2.30.0) (Quinlan 2014) to further group breakpoints that mapped near to each other but lacked reads long enough to intersect. The first condition ensured breakpoints that appeared distant to each other on the reference, but that were physically linked through fusion rearrangements, could be paired together. The second condition linked breakpoints that mapped near each other but that lacked reads long enough to span between them. The collection of HPV breakpoints that were grouped together through these two methods were referred to as an integration event. All read names belonging to an integration event were retained for later analyses. Integration event loci were defined as the integration breakpoints within an event that map within 500 kb of each other, as determined using BEDTools (v. 2.30.0) (Quinlan 2014). Integration events spanning multiple chromosomes or large genomic distances would have multiple integration event loci. The integration event loci were used for regional analyses such as finding recurrently integrated loci, determining expression changes of neighboring genes, and overlapping DMR hotspots.

### Detection of HPV integration event expression using HPV–human fusion transcripts

Previously described short-read RNA-seq data (Gagliardi et al. 2020) were used to detect HPV–human breakpoints in fusion transcripts. These data are available for download through the database of Genotypes and Phenotypes (dbGaP) (https://www.ncbi.nlm.nih.gov/gap/, phs000528), as part of the NCI Cancer Genome Characterization Initiative (CGCI; phs000235). The data were realigned to the GRCh38 HPVs reference genome using STAR (v. 2.7.11) (Dobin et al. 2013) with the following parameters, as described by Fan et al. (2023): ‐‐outSAMstrandField intronMotif, ‐‐chimSegmentMin 12, ‐‐chimJunctionOverhangMin 8, ‐‐chimOutJunctionFormat 1, ‐‐alignSJDBoverhangMin 10, ‐‐alignMatesGapMax 100000, ‐‐alignIntronMax 100000, ‐‐limitSjdbInsertNsj 1500000, ‐‐alignSJstitchMismatchNmax 5 -1 5 5, ‐‐outSAMattrRGline ID:GRPundef, ‐‐chimMultimapScoreRange 3, ‐‐chimScoreJunctionNonGTAG 4, ‐‐chimMultimapNmax 20, ‐‐chimNonchimScoreDropMin 10, ‐‐peOverlapNbasesMin 12, ‐‐peOverlapMMp 0.1, ‐‐alignInsertionFlush Right, ‐‐alignSplicedMateMapLminOverLmate 0 ‐‐alignSplicedMateMapLmin 30, ‐‐chimOutType Junctions WithinBAM SoftClip. RNA junctions between the HPV genome and human chromosomes were filtered to those with at least 10 congruent reads and within 100 kb of an ONT-called integration event using BEDTools (v. 2.30.0) (Quinlan 2014) and a custom Python script. Thus, expressed HPV integration events had at least one HPV–human RNA junction with at least 10 reads within 100 kb of an HPV breakpoint. The number of supporting reads for each HPV–human junction was used to determine the relative expression level of each junction per sample.

### Comparison of HPV integration calls between ONT and Illumina

Previously described short-read WGS data (Gagliardi et al. 2020), available for download through dbGaP (https://www.ncbi.nlm.nih.gov/gap/, phs000528) as part of the NCI Cancer Genome Characterization Initiative (CGCI; phs000235), were realigned to the hg38/HPVs reference genome using minimap2 (v. 2.15) (Li 2018). The short-read SV caller Manta (v. 1.6.0) (Chen et al. 2016) was used to call translocations between the human chromosomes and HPV genomes, and a minimum congruent read pair threshold of five reads was applied with BCFtools (v. 1.15.1) (Danecek et al. 2021). Integration breakpoints within 500 kb of each other were combined into integration events using BEDTools (v. 2.30.0) (Quinlan 2014). Subsequently, integration breakpoint calls for 69 HTMCP samples (those with integration detected using one or both technologies) were summed and compared by sample and by event. The genomic overlap of integration breakpoint calls for Illumina and ONT was determined using the BEDTools (v. 2.27.1) (Quinlan 2014) intersect function against hg38 annotations obtained from the Table Browser page on the University of California, Santa Cruz website (http://genome.ucsc.edu) (Karolchik et al. 2004). Specifically, the GENCODE V43 knownGene annotation (Harrow et al. 2012) filtered to exons or introns, cpgIslandExt, and RepeatMasker (Chen 2004) annotations were downloaded as BED files (accessed May 2, 2023).

### Assembling HPV integration event contigs

Each HPV integration event was assembled into an integration contig for characterization. The events were first subsetted into event-only BAM files with Picard tools’ (v. 2.26.6; https://broadinstitute.github.io/picard/) FilterSamReads function using the read name text files created when grouping the integration events. The reads were then converted into FASTQ files using SAMtools (v. 1.12) (Li et al. 2009). Each set of event reads was then run through the assembler Flye (v. 2.9) (Kolmogorov et al. 2019) with three rounds of polishing. The assembly was mapped back to the reference chromosome for assembly annotation and the reads were mapped back to the assembly to check assembly quality using minimap2 (v. 2.23) (Li 2018). Sniffles (v. 1.0.12) (Sedlazeck et al. 2018) was run on the reads aligned to the assembly to check for rearrangements that may not be assembled correctly, such as insertions, deletions, and duplications.

### Detection of potential ecDNAs

Circularity was tested on all events to discover putative ecDNA events that were predicted to be extrachromosomal. The assemblies of events that successfully assembled were aligned to the reference genome using minimap2 (v. 2.23) (Li 2018). The alignments of the event reads were then subtracted using BEDTools (v. 2.30.0) (Quinlan 2014) from the assembly alignment. If there was minimal to no coverage outside the assembly region and there was adequate coverage (>2 reads) on each border of the assembly, and the assembly contained a contig that was predicted as circular by Flye (v. 2.9) (Kolmogorov et al. 2019), then the event was categorized as a putative ecDNA.

### Categorizing HPV integration events

The categorization of the integration events followed the decision chart in Fig. 1C. Repeats were detected on the FASTA files of the reads using RepeatMasker (v. 4.2.1) (Chen 2004). The output GFF file was then checked for the following repeat sequences: TAR1, ALR, HSAT4, HSAT5, HSAT6, and SST1. If over 50% of the reads contained repeat sequences, then the integration event was categorized as a repeat integration. If no repeat was detected, then the number of breakpoints in the event was counted. Events with more than two breakpoints were categorized as multi-breakpoint events. Two-breakpoint events were then categorized as translocation integration if the two breakpoints were on different chromosomes, del-like integration if the region in between the two breakpoints had no read coverage in the HPV-containing reads, and dup-like integration if there was read coverage between the breakpoints. Circularity was tested separately from this categorization based on assembly as outlined above.

### HPV integrant copy number calling

The HPV-containing read alignments in PAF format were analyzed using an R script (v. R 4.0; http://www.r-project.org) script to determine HPV integrant lengths in a read-specific manner. Reads containing the same human and HPV breakpoints were grouped together, allowing a leniency in the exact breakpoints of ±30 bp. The HPV alignments that occurred between two SV-detected breakpoints were extracted from each read and the total length of the HPV integrant was determined by its cumulative length on the read. If only one breakpoint was detected, the length of the HPV alignments before or after the break was calculated for analyses of incomplete HPV integrants. All unique breakpoint pairs (or incomplete breakpoints) were determined for each sample. HPV integrant sizes for each breakpoint pair were separated into read groups if there was a difference of >300 bp to the next closest size. Reads with integrant sizes within <300 bp of each other were therefore grouped together. Each unique breakpoint pair or breakpoint was the categorized: “heterologous” if >1 integrant size group was detected, “partial” if the HPV integrant was <1 HPV genome equivalent in size, “full” if the HPV integrant was >1 HPV genome equivalent in size, and “incomplete” if the breakpoint was not paired. The maximum integrant size of a heterologous integrant was determined by the mean size of the largest read group. The maximum size of an incomplete integrant was determined by the read containing the longest HPV alignment before or after the breakpoint.

### Comparison of two-breakpoint events

The genomic distance between breakpoints was calculated for all two-breakpoint events by subtracting the reference position of the most 5′ breakpoint from the most 3′ breakpoint. A Wilcoxon ranked-sum test was used to calculate the significance of the difference between dup-like and del-like events. The position of the two-breakpoint events relative to genic regions was determined by intersecting the region between the two breakpoints (event region) with the gene regions (Ensembl 100 GRCh38) using BEDTools (v. 2.30.0) (Quinlan 2014). The events were categorized as “genic” if >90% of the event region fell within a gene, “partially genic” if the event was between 10% and 90% within a gene, and “intergenic” if <10% of the event region was within a gene. The proportions of events that were genic, partially genic, and intergenic were compared using a Fisher's exact test.

### Copy number analysis

The copy number profiles (determined using Illumina WGS) were obtained for HTMCP-03-06-02054 and other samples containing translocation events. The Ploidetect (v. 3.0) (Culibrk et al. 2024) pipeline was run using the “short” sequence type (see https://github.com/lculibrk/Ploidetect-pipeline, section 1.1.4). The results were selected for the first predicted model that did not have a ploidy of 1.

### Multi-breakpoint event reconstruction and visualization

The multi-breakpoint event loci were visualized using a custom R script (v. R 4.0; http://www.r-project.org) that positioned the HPV integrant connections. The HPV breakpoint pairs were grouped into their respective events, and the selected multi-breakpoint events in Figure 4D were visualized by connecting the breakpoints within a breakpoint pair using an arched dotted line. The heterologous integrants were colored differently from integrants with a single integrant structure. The number of connections each breakpoint participated in (i.e., the number of breakpoint pairs it belonged to) was also indicated by the color of the dot. Read depth of the example event in Figure 4E was calculated using SAMtools (v. 1.12) (Li et al. 2009) in the regions between each breakpoint pair. SV breakpoints were mapped to the HPV integration events by using overlapping read names, as done for assembling HPV breakpoints into events, using a custom Python script.

### HPV genome methylation, flanking methylation, and flanking expression

The HPV genome methylation frequencies per CpG were calculated using the HPV-containing reads from each group of reads belonging to a single HPV integrant. These reads were used to calculate the average methylation flanking the integrant for del-like and dup-like integration events. For the flanking regions, methylation frequencies were averaged across 500 bp bins up to 5000 bp upstream and downstream from the breakpoints on the human DNA. The upstream/downstream directions were oriented according to the strandedness of the HPV integrant such that the directions were relative to that of HPV transcription. For visualization, the HPV genome positions were shifted to begin at the *E6* start position and end at the *L1* stop position, with all LCR positions represented as negative values so they could be visualized together.

The stranded position of HPV–human RNA fusion sites was compared to the relative position of the nearest DNA integration breakpoint. For assembly, we realigned RNA-seq data to the HPV integration event contig using minimap (v. 2.15) (Li 2018) and remapped the methylation and transcript fusion sites for visualization. For del-like and dup-like integration events, we calculated the RPKM of the RNA-seq reads in the 5 kb region upstream and downstream from HPV and used these normalized expression values to compare events. We analyzed a 0–2.5 kb window around HPV for upstream and downstream methylation due to read length limitations in some events. The average methylation value was calculated by averaging the methylation frequencies across all reads, and an average methylation frequency of <25% in the downstream region was considered “unmethylated.”

### Hotspots of DMRs

DMRs were identified using the DSS (Feng and Wu 2019) 2-sample analysis. DMR hotspots were identified using an R script (v. R 4.0; http://www.r-project.org) that compared the actual density of DMRs across each chromosome to a Gaussian null distribution of the same number of DMRs. DMR hotspots were defined as regions where the actual density of DMRs was significantly higher than the null distribution. These hotspots were intersected with the HPV integration event locations using BEDTools (v. 2.30.0) (Quinlan 2014). Events and hotspots on Chromosome X were excluded. The phase block borders of the HPV integration events were also identified by intersecting with BEDTools (v. 2.30.0) (Quinlan 2014), and the haplotype of the integration event was determined as the haplotype that contained the majority of HPV-containing reads at that integration event locus. The *P*-values for the enrichment of DMRs upstream and downstream from the HPV integration locus were calculated by generating 1000 random genomic regions of the same size as the test region (500 kb), calculating the DMR density within these regions for the test sample and all other samples, calculating the fold change in DMR density between the test sample vs. the mean of all other samples in each region, and determining the percentile of the test region's fold change compared to the null distribution of the 1000 random regions.

### Association of DMRs with HPV integration across tumors

The DMR density at five window sizes (100 kb, 500 kb, 1 Mb, 2 Mb, and 5 Mb) was calculated by dividing the number of bases within a DMR by the window size. We compared the HPV-integrated loci (*n* = 147) to a control set (*n* = 10,000) to determine significance. The control set was generated by first calculating the GC-content distribution of the HPV-integrated test regions HPV-integrated at the five window sizes. We then measured the background distribution of GC content in sliding windows of the same window sizes (100 kb, 500 kb, 1 Mb, 2 Mb, and 5 Mb) across the genome (sliding by 100 kb for each. We selected 10,000 control regions to create a GC-content distribution that mimics the test regions’ distribution and calculated the DMR density in randomly selected samples for each. We then compared the DMR density at the test regions to the control regions using a Benjamini–Hochberg-adjusted Wilcoxon rank-sum test.

### Accession of gene expression data

The gene expression data for the HTMCP cohort (Gagliardi et al. 2020) was downloaded from the GDC data portal on August 31^st^, 2023 (https://gdc.cancer.gov/). The transcripts per million (TPM) normalized values were used to create an expression matrix of the 72 samples used in this study.

### Gene expression outliers and allele-specific expression analysis

The distance between protein-coding genes (Ensembl 100 GRCh38) and integration event loci was calculated using BEDTools (v. 2.30.0) (Quinlan 2014). Genes that were within ±1 Mb of the integration events were tested to see if they were expression outliers, i.e., more than 1.5 IQR below Q1 or 1.5 IQR above Q3. The fold change of the outliers was calculated as log_2_(TPM of the tested sample/TPM of the median sample). ASE was determined using IMPALA using the phased VCF (Chang et al. 2023). Genes with ASE were defined as genes that had a major allele frequency threshold greater than 0.65 and an adjusted *P*-value <0.05. Genes that were unable to be tested for ASE did not contain a phased single nucleotide variant (SNV) within the genic region.

### Statistical analyses

No sample sizes other than previously specified molecular features of the initial data set were predetermined. Unless otherwise stated, all statistical tests correspond to two-sided tests. *P*-value methods and multiple-test correction are reported in the text. Wilcoxon in the text refers to the Wilcoxon rank-sum test.

## Data access

The whole-genome long-read sequencing data generated in this study have been submitted to the dbGaP database (https://www.ncbi.nlm.nih.gov/gap/) under accession number phs003780. Data analysis methods and source code can be found as Supplemental Code and as open-source workflows on GitHub (https://github.com/MarraLab/VPorter_HPV_integration).

## Supplemental Material

Supplement 1

Supplement 2

Supplement 3

Supplement 4

Supplement 5

Supplement 6

Supplement 7

Supplement 8

Supplement 9

Supplement 10

Supplement 11

Supplement 12

Supplement 13

Supplement 14

Supplement 15
