# Supplementary material for: Rearrangements of viral and human genomes at human papillomavirus integration events and their allele-specific impacts on cancer genome regulation
Source: Genome Res. 2025 Apr;35(4):653–70. doi: 10.1101/gr.279041.124 (PMC12047271; doi:10.1101/gr.279041.124)
Supplement: Supplement 1 [file Supplemental_Figures.pdf]

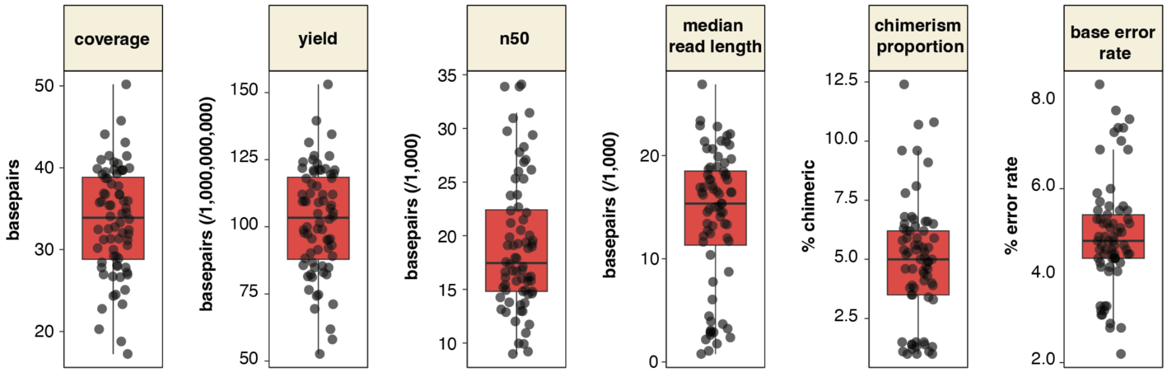

Supplemental Figure S1. Sequencing statistics of ONT long-read sequencing. The coverage, yield, number of reads, n50, median read length, chimerism rate, and base error rate of the 72 HTMCP samples sequenced using whole genome ONT long-read technology. Box plots represent the median and upper and lower quartiles of the distribution; whiskers represent the limits of the distribution (1.5 IQR below Q1 or 1.5 IQR above Q3).

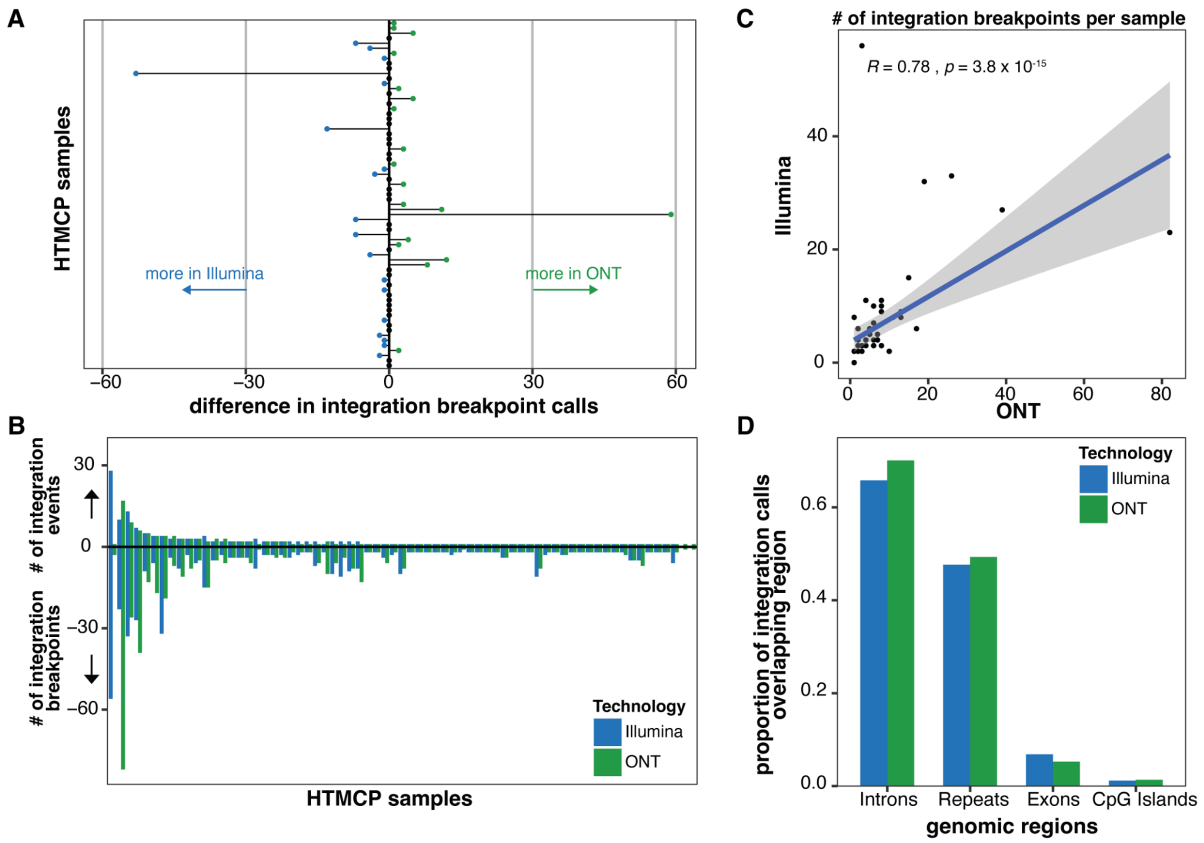

Supplemental Figure S2. Comparison of HPV integration calling using Illumina short-read sequencing and ONT long-read sequencing. (*A*) The differences in the number of HPV integration breakpoint calls per sample in short-read (Illumina) and long-read (ONT) WGS. For samples where more breakpoints were detected by short-reads, the difference is shown to the left, and where more breakpoints were detected by long-reads, the difference is shown to the right. (*B*) The number of integration breakpoints and events in short-read (Illumina) and long-read (ONT) WGS. (*C*) The Spearman's correlation between the number of HPV breakpoint calls per sample as determined by long-read sequencing (ONT) vs. short-read sequencing (Illumina). (*D*) Comparison of the genomic regions harboring breakpoint calls in the short-read (Illumina) and long-read (ONT) WGS.

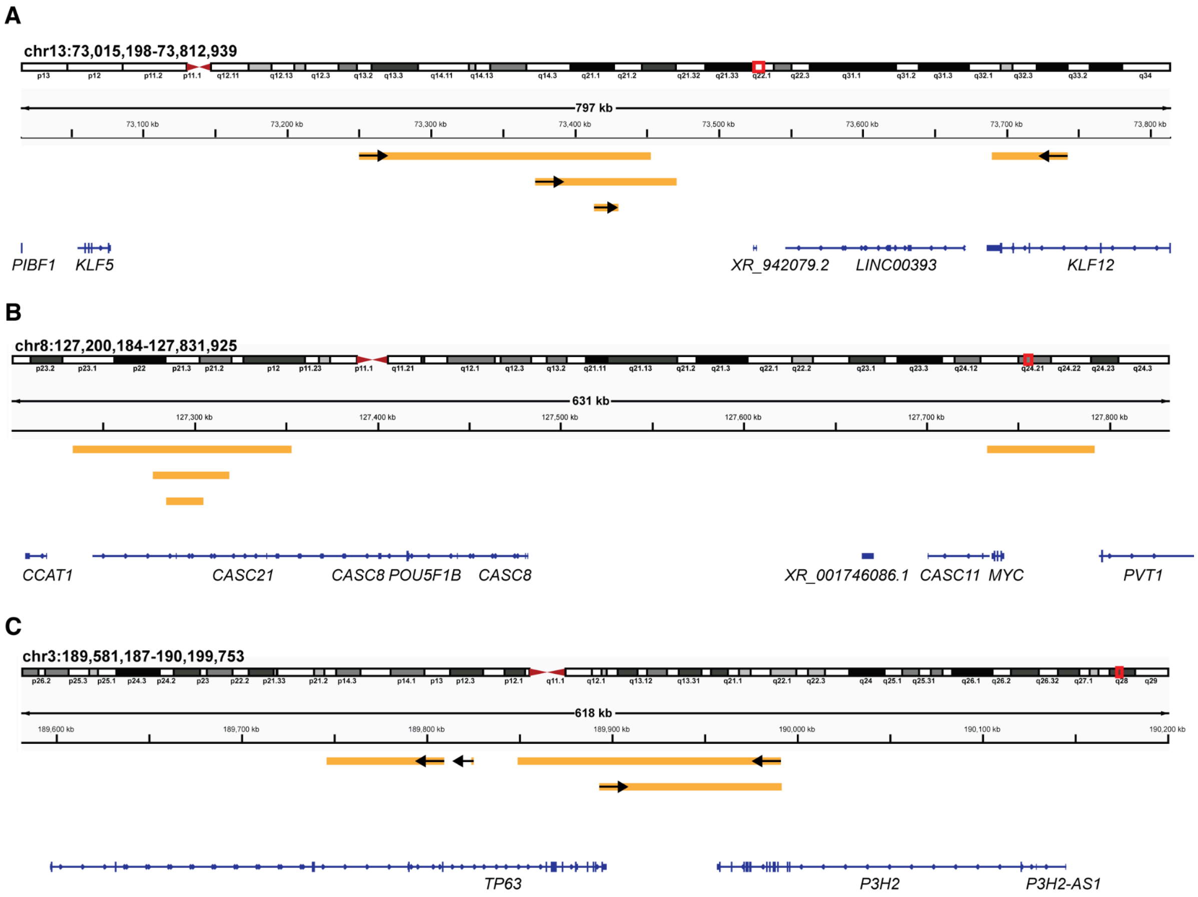

Supplemental Figure S3. The distribution of events at loci recurrently affected by HPV integration. (*A*) The four dup-like events in the intergenic region between *KLF5* and *KLF12* at the 13q22 locus. (*B*) The four multi-breakpoint events in the 8q24 locus around *MYC*. (*C*) The four dup-like events in the 3q28 locus around *TP63*. The integration events were visualized in the Integrative Genomics Viewer, with the integration events represented as regions spanning the 5' and 3' integration breakpoints. The direction of HPV transcription at the two breakpoint events (all *MYC*-locus events were multi-breakpoint) is represented by a black arrow.

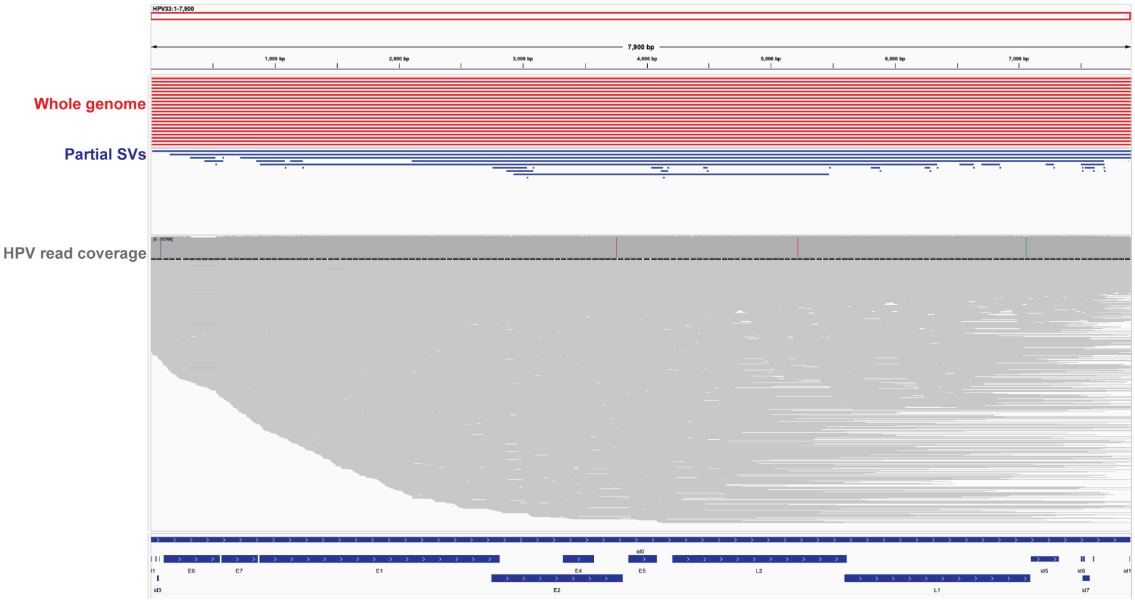

Supplemental Figure S4. Episomal SVs across the HPV33 genome in HTMCP-03-06-02156. Integrative Genomics Viewer snapshot of viral rearrangements in an episomal sample. The virus-virus structural variants (SVs) are shown in blue and span partial regions throughout the episome. The HPV read coverage and HPV33 gene positions are also shown below.

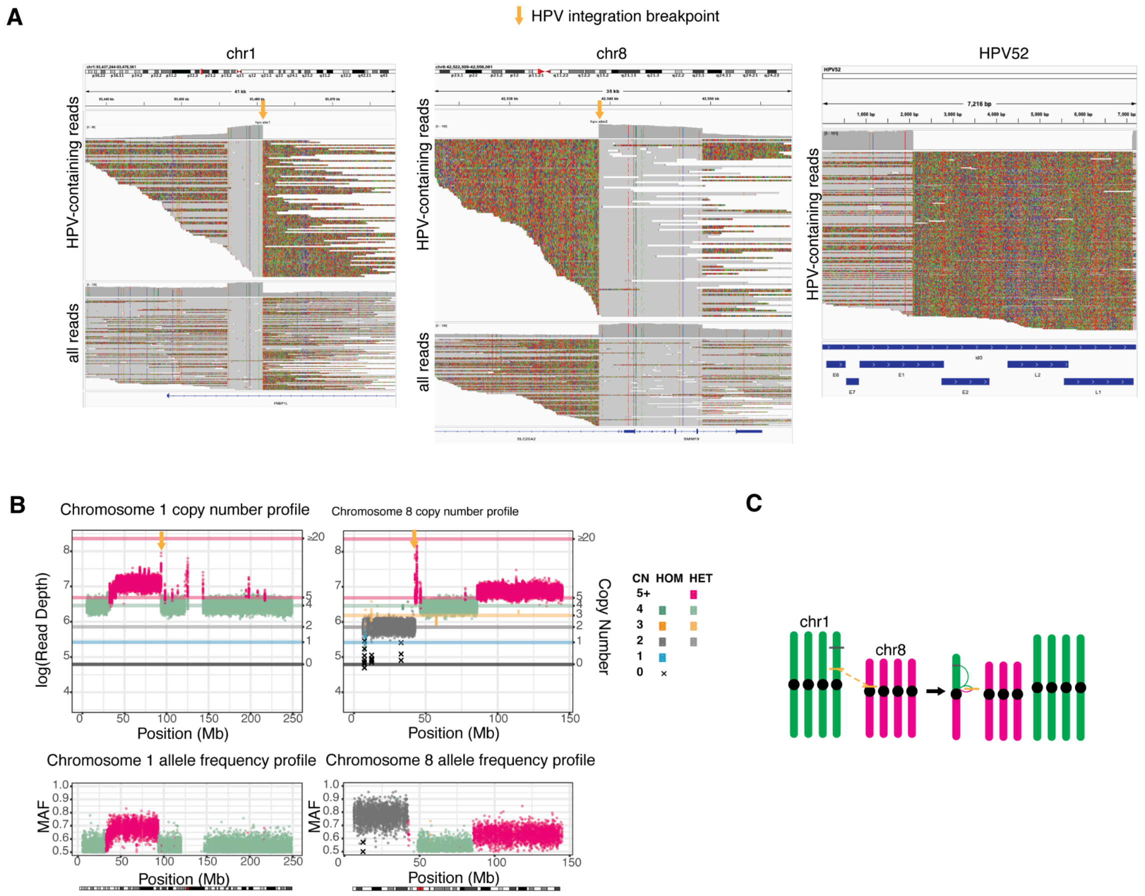

Supplemental Figure S5. Visualization of a translocation integration event. (*A*) Integrative Genomics Viewer snapshots with mismatched bases turned on and supplemental alignments included showing a HPV translocation event between Chromosome 1 (chr1) and Chromosome 8 (chr8). (*B*) The copy number profiles of Chromosome 1 and Chromosome 8, as determined using Illumina WGS. (*C*) Schematic illustrating the inferred chromosome arm exchange resulting from the translocation integration events in *A*. Orange arrows and lines denote the HPV integration breakpoints.

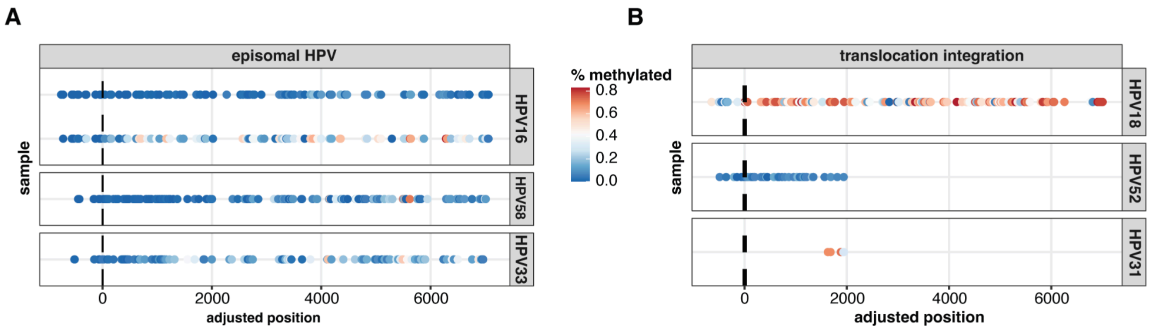

Supplemental Figure S6. The methylation frequency within HPV episomes and HPV integrants in (*A)* episomal HPV and (*B)* translocation events. Within HPV, the methylation of each CpG is shown as a point and colored by the methylation frequency. All the events are aligned to the start of the genic region (*E6* start) for each respective HPV type.

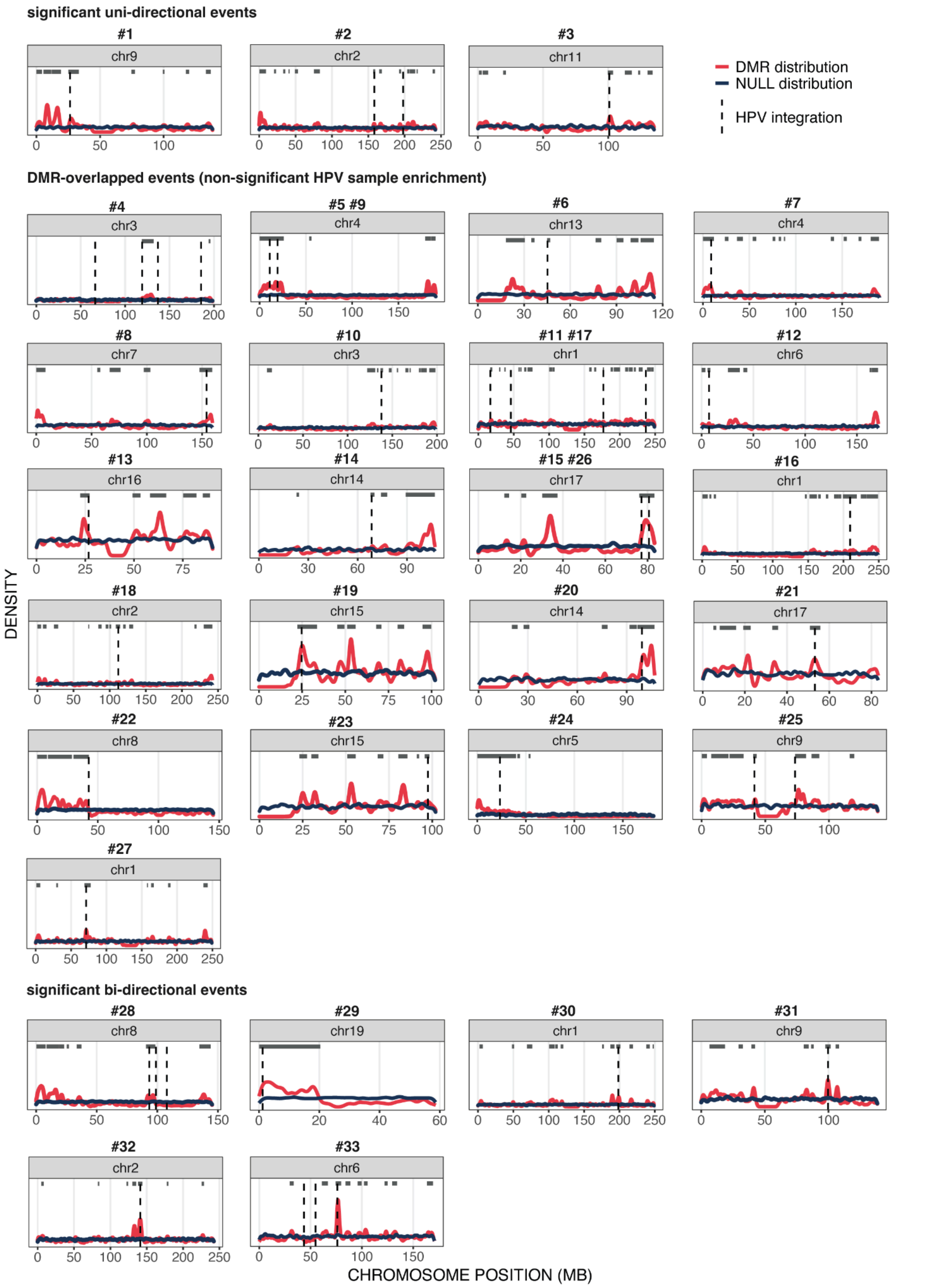

Supplemental Figure S7. DMR density hotspots on chromosomes harboring HPV integration. Chromosome-wide DMR density plots of all HPV integration events overlapping DMR hotspots. A null Bayesian distribution was compared to the actual distribution. Regions where the distributions significantly deviate are shown above as grey boxes. The dotted lines indicate sites of HPV integration.

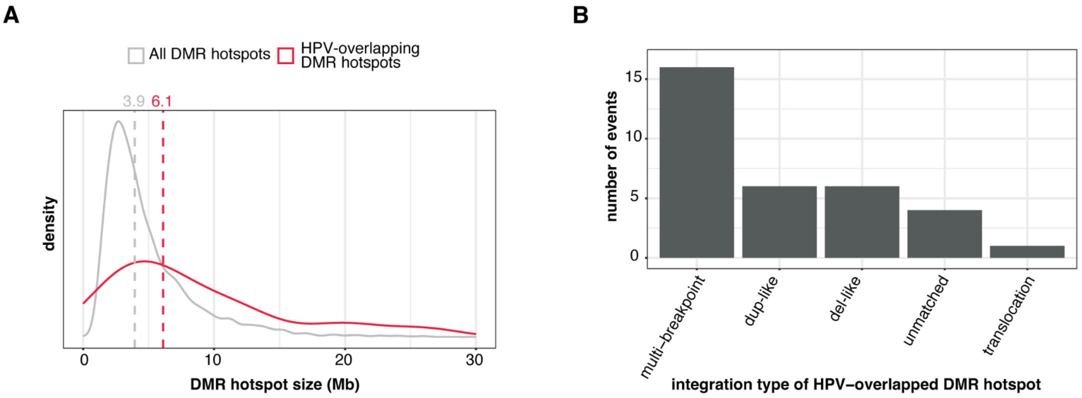

Supplemental Figure S8. Descriptive qualities of the HPV-overlapping DMR hotspots. (*A*) The genomic span of HPV-associated DMR hotspots compared to all DMR hotspots across the cohort. The dotted lines denote the median of each distribution. (*B*) The number of HPV integration events overlapping a DMR hotspot across the different integration categories.

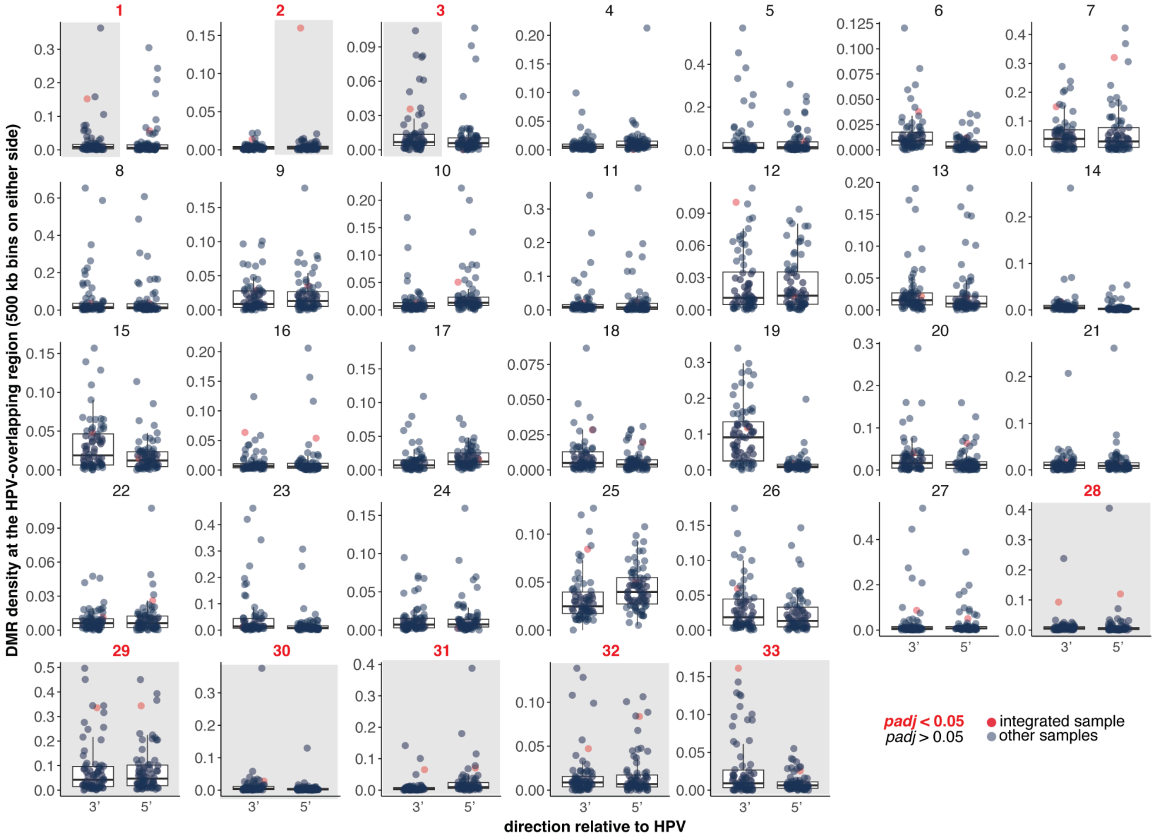

Supplemental Figure S9. Statistical testing of the 33 integration events overlapping DMR hotspots. For each integration event, the DMR density in the integrated sample was compared to all other samples in the cohort on both the 500 kb regions on either side of the event. *P* values were determined by a permutation test across the genome (*n* = 1000) with the window size equal to the tested region (500 kb). The events numbered in red were deemed significant (*padj* < 0.05) in at least one of the tested regions (3' or 5'). The significant regions are highlighted by grey boxes. Events 1-3 have significant uni-directional DMR enrichment, events 28-33 have significant bi-directional DMR enrichment, and the remaining events were not significant. Box plots represent the median and upper and lower quartiles of the distribution; whiskers represent the limits of the distribution (1.5 IQR below Q1 or 1.5 IQR above Q3).

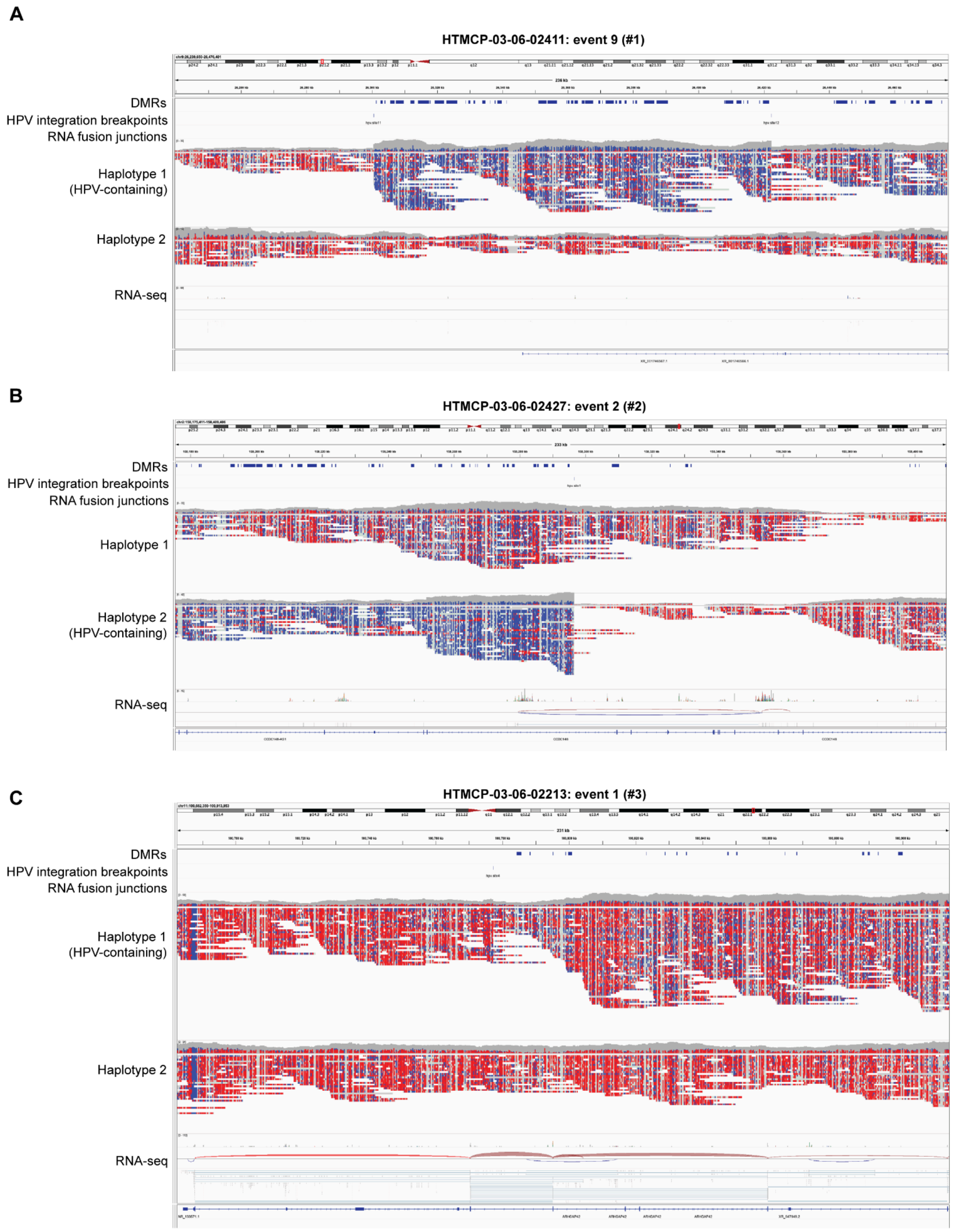

Supplemental Figure S10. Examples of differential methylation at HPV integration events overlapping uni-directional DMR hotspots. (*A*-*C*) Integrative Genomics Viewer snapshots of the genomic regions surrounding HPV integration events, including (*A*) HTMCP-03-06-02411: event 9 (dup-like event; #1), (*B*) HTMCP-03-06-02427: event 2 (unmatched event; #2), and (*C*) HTMCP-03-06-02213: event 1 (unmatched event; #3). The reads are separated by haplotype and CpGs are colored by methylation status (red = methylated, blue = unmethylated). Tracks showing the HPV integration breakpoints, the HPV-human RNA fusion sites, the DMRs, and the RNA-seq read coverage are also included in the snapshots. All three events did not have any evidence of expression in the RNA-seq data.

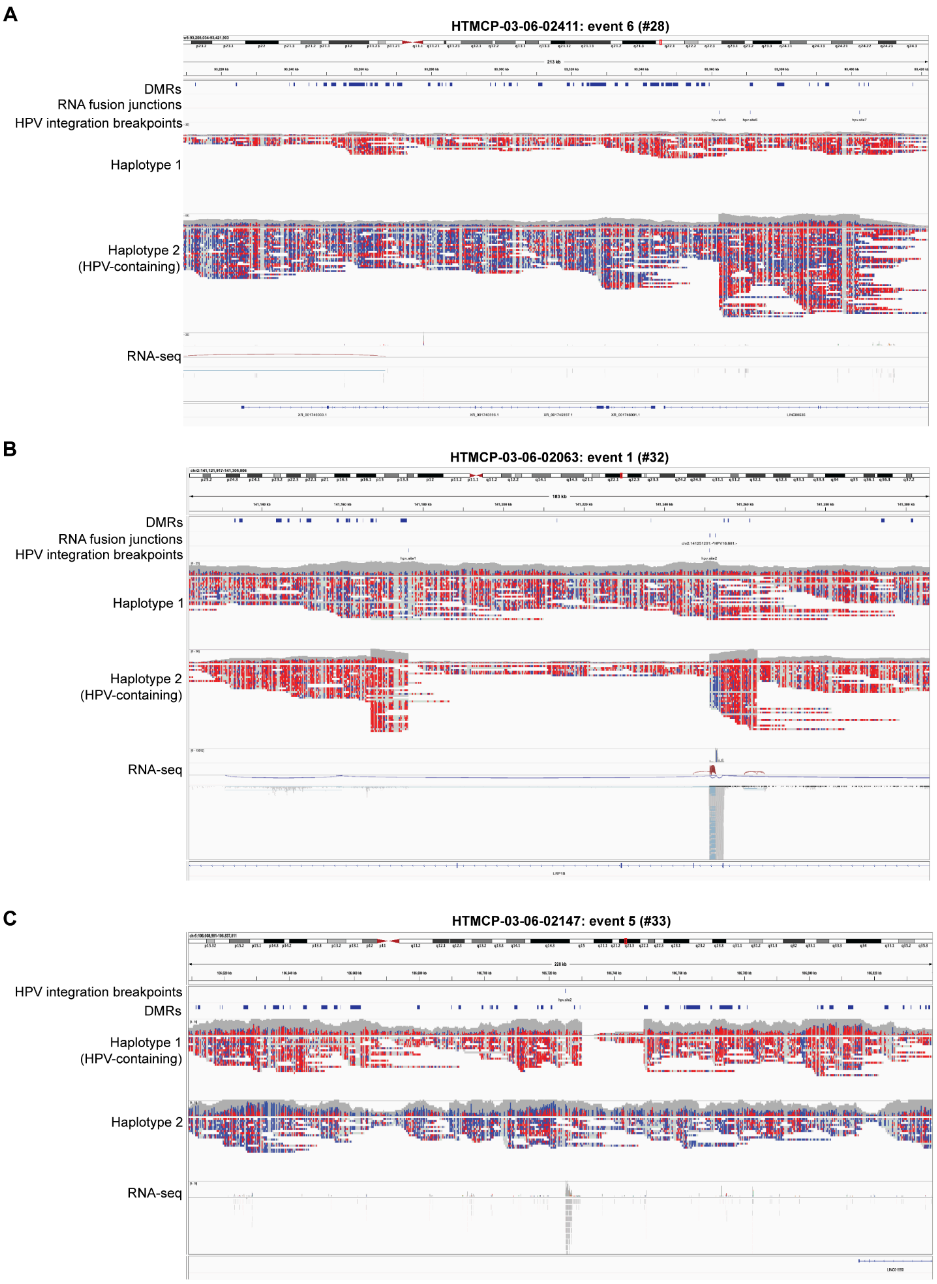

Supplemental Figure S11. Examples of differential methylation at HPV integration events overlapping bi-directional DMR hotspots. (*A*) Integrative Genomics Viewer snapshot of a multi-breakpoint event without any HPV-human RNA junctions detected (HTMCP-03-06-02411: event 6; #28). This example is unmethylated on the integrated allele (haplotype 2) and does not have any evidence of expression across the locus. (*B*) Integrative Genomics Viewer snapshot of a deletion event with HPV-human RNA junctions (HTMCP-03-06-02063: event 1; #32). This example is overall methylated on the integrated allele (haplotype 2) but an unmethylated segment overlaps the HPV-human fusion transcript. (*C*) Integrative Genomics Viewer snapshot of a dup-like event without any HPV-human RNA junctions detected (HTMCP-03-06-02147: event 5; #33). This example is methylated on the integrated allele (haplotype 2) and does not have any evidence of expression across the locus. The reads are separated by haplotype and CpGs are colored by methylation status (red = methylated, blue = unmethylated). Tracks showing the HPV integration breakpoints, HPV-human RNA fusion junctions, DMRs, and RNA-seq reads are also included in the snapshots.

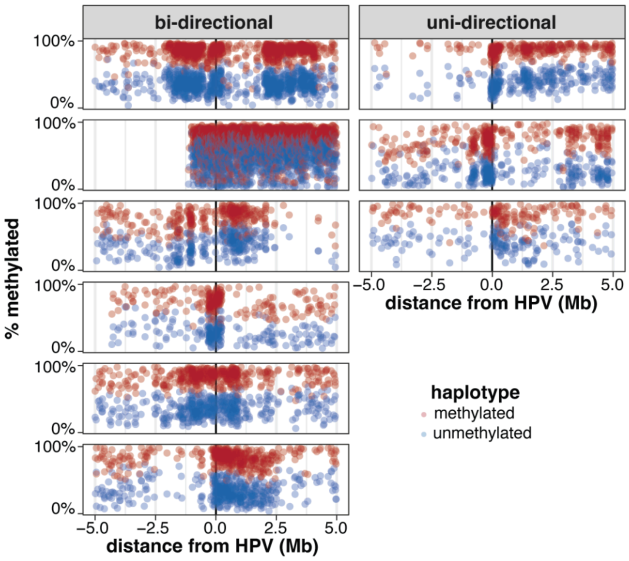

Supplemental Figure S12. A zoomed view of the DMR positions in the nine significantly DMR-enriched HPV integration event loci. The methylation frequencies of the two haplotypes are shown in blue and red, depending if they are the more or less methylated haplotype. The empty values on the negative end of the second bi-directional event are due to reaching the end of the chromosome.

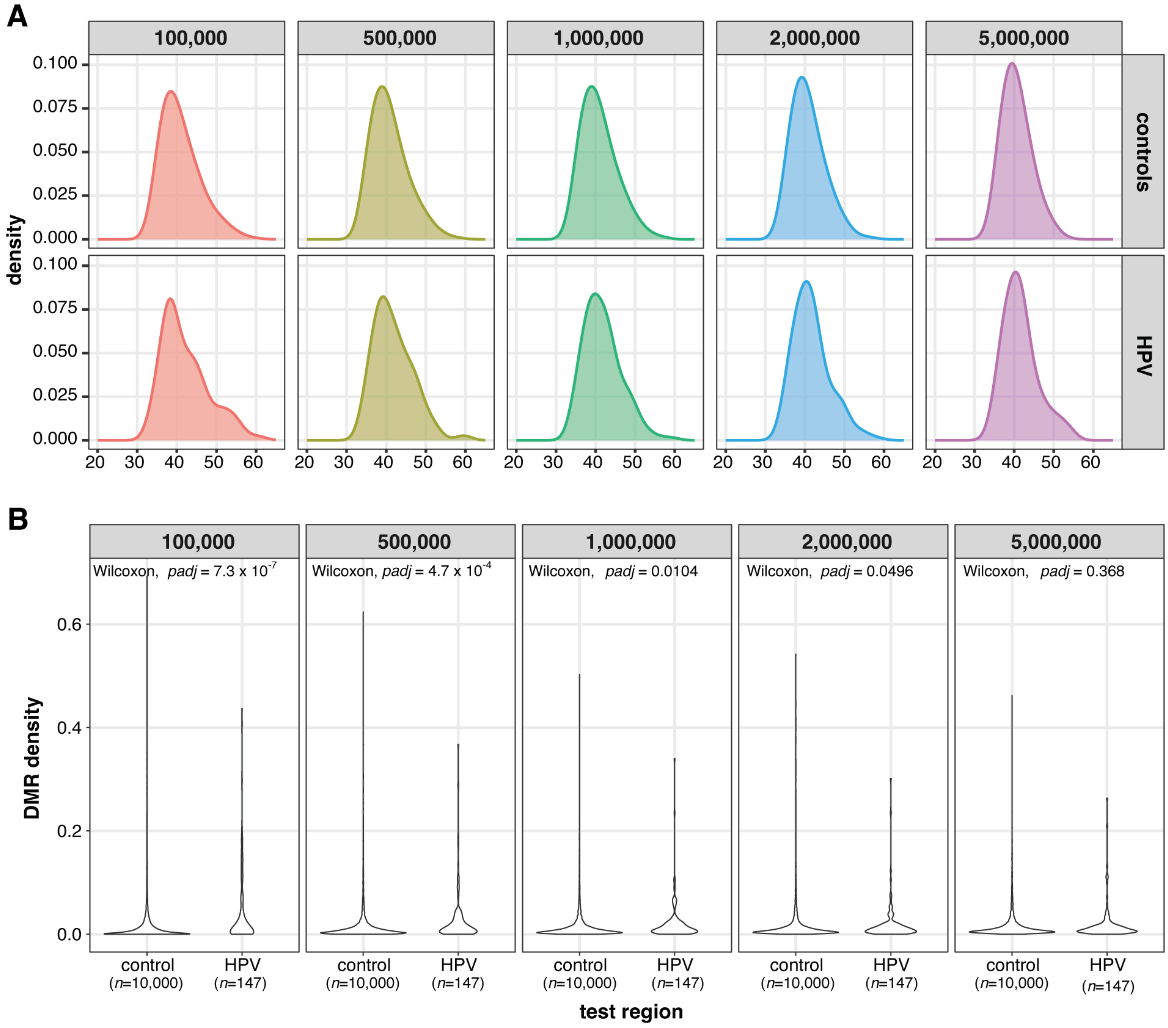

Supplemental Figure S13. High DMR density is found in genomic windows surrounding HPV integration. (*A*) The GC-content distribution of the 10,000 control regions and the HPV-integrated regions (*n*=147) at the specified window sizes, including 100,000 bp, 500,00 bp, 1,000,000 bp, 2,000,000 bp, and 5,000,000 bp. The control regions were chosen randomly to simulate the same distribution of the HPV region windows of the same size. (*B*) The DMR density at the 10,000 control regions vs. the HPV containing windows. The window sizes 100,000 bp, 500,00 bp, 1,000,000 bp, and 2,000,000 bp were found to be significant. Benjamini-Hochberg adjusted *P* values were calculated using the Wilcoxon rank-sum test.

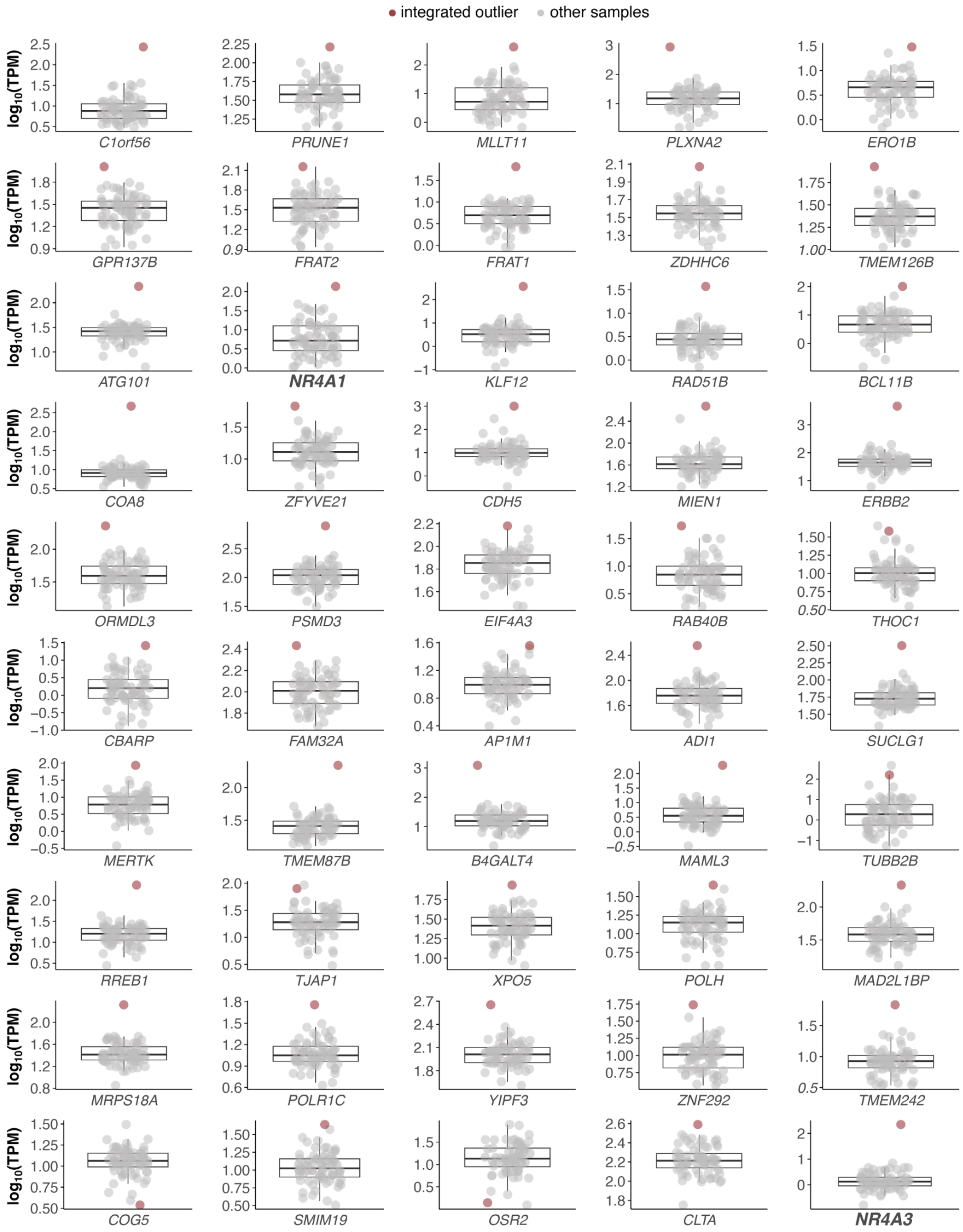

Supplemental Figure S14. Gene expression differences between integrated samples with outlier gene expression nearby (+/- 200 kb) HPV integration and other samples without HPV integration in that region. Genes of interest *NR4A1* and *NR4A3* are bolded. Box plots represent the median and upper and lower quartiles of the distribution; whiskers represent the limits of the distribution (1.5 IQR below Q1 or 1.5 IQR above Q3).

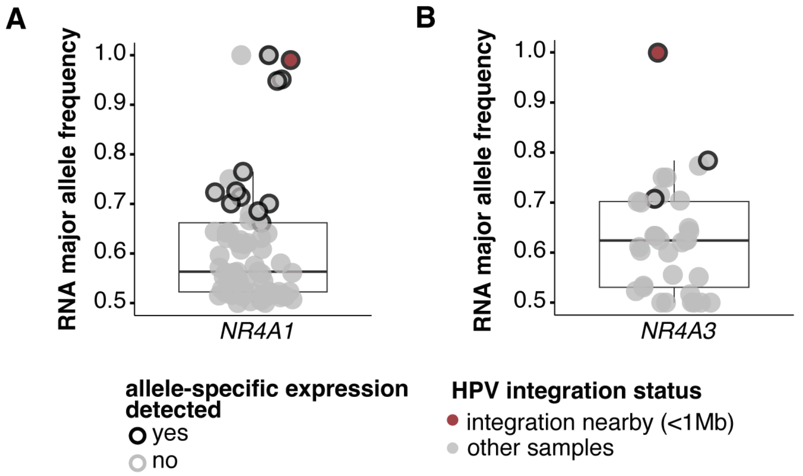

Supplemental Figure S15. The RNA-seq major allele frequency and ASE of (*A*) *NR4A1* and (*B*) *NR4A3* in the sample with HPV integration nearby compared to the other samples. Box plots represent the median and upper and lower quartiles of the distribution; whiskers represent the limits of the distribution (1.5 IQR below Q1 or 1.5 IQR above Q3).

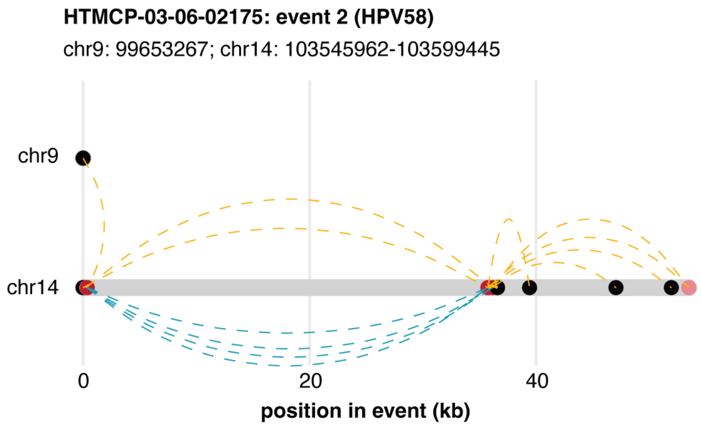

Supplemental Figure S16. The breakpoint structure of HTMCP-03-06-02428's event 1 neighboring *NR4A3* on chromosome 9. Dots denote HPV breakpoints along the event, and dotted lines represent the HPV integrants that connect the breakpoints. The dots are colored according to the number of connections that converge at that position in the event. The integrants are colored according to whether single (orange) or heterologous (blue) integrant structures connect the breakpoints.

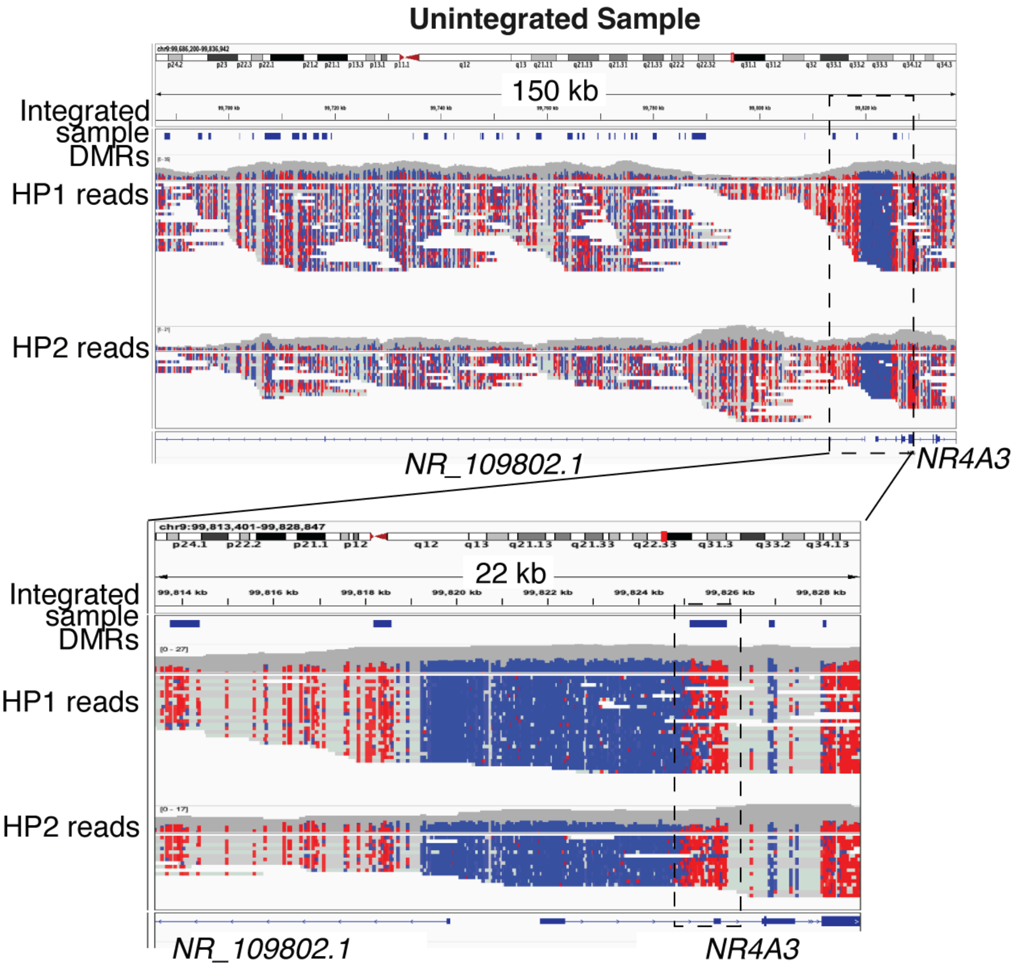

Supplemental Figure S17. Integrative Genomics Viewer snapshots showing wide (top) and zoomed (bottom) views of the haplotype-specific methylation changes around *NR4A3* in a sample without HPV integration within the region, with reads separated into the two haplotypes (HP1 and HP2). The integrated sample's DMRs are also indicated in the top track. Reads are colored by CpG methylation status, with red indicating methylated and blue indicating unmethylated.

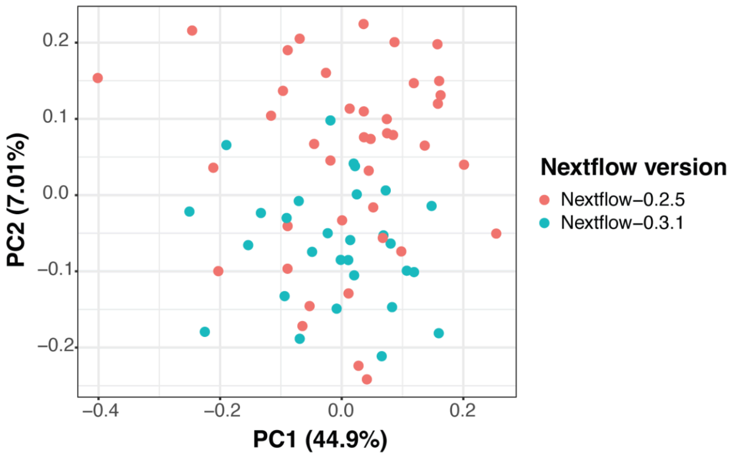

Supplemental Figure S18. Principal component clustering analysis on the average methylation at the 463 UCSC annotated CpG islands with each sample coloured by the Nextflow version used. Nextflow-0.2.5 was run on R9 flow cells, while Nextflow-0.3.1 was run on R10 flow cells.
