# Supplementary material for: Rearrangements of viral and human genomes at human papillomavirus integration events and their allele-specific impacts on cancer genome regulation
Source: Genome Res. 2025 Apr;35(4):653–70. doi: 10.1101/gr.279041.124 (PMC12047271; doi:10.1101/gr.279041.124)
Supplement: Supplement 15 [file Supplemental_Code.zip › Supplemental_Code/Porter-ONT-HPV-FigureScripts2023/figures/AllFigsMaster.html]

Manuscript Figures


# Manuscript Figures

#### Dr. Vanessa Porter

#### 2024-06-21

## Categorizing HPV Integration Events (Figure 1)

In this section of the analysis, we organize the workflow outputs to
summarize the categorization of HPV integration events. These figures
make up part of Figure 1 in the manuscript.

#### Integration event types

Here we are reading in the integration event types from the workflow
output. Each integration event is categorized as one of the following
based on breakpoint numbers, breakpoint positions, and read alignment
patterns:

1. dup-like integration
2. del-like integration
3. multi-breakpoint integration
4. translocation integration
5. repeat integration
6. unmatched integration

```
dim(int)
```

```
## [1] 129   3
```

```
kable(head(int))
```

| sample | event | type |
| --- | --- | --- |
| HTMCP-03-06-02001 | event1 | dup-like\_integration |
| HTMCP-03-06-02006 | event1 | del-like\_integration |
| HTMCP-03-06-02020 | event1 | dup-like\_integration |
| HTMCP-03-06-02037 | event1 | multi-breakpoint\_integration |
| HTMCP-03-06-02040 | event1 | del-like\_integration |
| HTMCP-03-06-02042 | event1 | del-like\_integration |

#### Integration breakpoints and events

We will also read in the event summary data, which groups each
integration breakpoint into an integration event.

```
dim(sum)
```

```
## [1] 441   9
```

```
kable(head(sum))
```

| sample | chr | pos | HPVchr | HPVpos | HPV.site | event | VAF | read.depth |
| --- | --- | --- | --- | --- | --- | --- | --- | --- |
| HTMCP-03-06-02001 | chr3 | 189823818 | HPV16 | 3973 | hpv.site1 | event1 | 0.491124 | 83 |
| HTMCP-03-06-02001 | chr3 | 189825181 | HPV16 | 3691 | hpv.site2 | event1 | 0.394737 | 60 |
| HTMCP-03-06-02006 | chr12 | 45934723 | HPV45 | 5340 | hpv.site1 | event1 | 0.246988 | 41 |
| HTMCP-03-06-02006 | chr12 | 45934737 | HPV45 | 1623 | hpv.site2 | event1 | 0.270588 | 46 |
| HTMCP-03-06-02020 | chr4 | 102391305 | HPV45 | 4282 | hpv.site1 | event1 | 0.342105 | 13 |
| HTMCP-03-06-02020 | chr4 | 102429177 | HPV45 | 2979 | hpv.site2 | event1 | 0.372093 | 16 |

#### Add the episomal samples to the event summary list

Events without any integration detected do not have an integration
type, so we will add these to the summary file so all samples are
included.

```
kable(head(summary1))
```

| sample | event | n |
| --- | --- | --- |
| HTMCP-03-06-02001 | event1 | 2 |
| HTMCP-03-06-02006 | event1 | 2 |
| HTMCP-03-06-02020 | event1 | 2 |
| HTMCP-03-06-02037 | event1 | 6 |
| HTMCP-03-06-02040 | event1 | 2 |
| HTMCP-03-06-02042 | event1 | 2 |

#### Add the transcriptional status to the summary files

The RNA HPV-human fusion breakpoints were detected using short-read
RNA-seq. These fusions were then intersected with the ONT-called
integration events to determine which event(s) had evidence of
expression in a sample.

```
# add the transcriptional status to the summary dataframes
sum$is.event.transcribed <- ifelse(paste0(sum$sample, sum$event) %in% paste0(ehpv$sample, ehpv$event), "yes", "no")
intOnly$is.event.transcribed <- ifelse(paste0(intOnly$sample, intOnly$event) %in% paste0(ehpv$sample, ehpv$event), "yes", "no")

kable(head(sum))
```

| sample | chr | pos | HPVchr | HPVpos | HPV.site | event | VAF | read.depth | integration\_type | is.event.transcribed |
| --- | --- | --- | --- | --- | --- | --- | --- | --- | --- | --- |
| HTMCP-03-06-02001 | chr3 | 189823818 | HPV16 | 3973 | hpv.site1 | event1 | 0.491124 | 83 | dup-like\_integration | yes |
| HTMCP-03-06-02001 | chr3 | 189825181 | HPV16 | 3691 | hpv.site2 | event1 | 0.394737 | 60 | dup-like\_integration | yes |
| HTMCP-03-06-02006 | chr12 | 45934723 | HPV45 | 5340 | hpv.site1 | event1 | 0.246988 | 41 | del-like\_integration | yes |
| HTMCP-03-06-02006 | chr12 | 45934737 | HPV45 | 1623 | hpv.site2 | event1 | 0.270588 | 46 | del-like\_integration | yes |
| HTMCP-03-06-02020 | chr4 | 102391305 | HPV45 | 4282 | hpv.site1 | event1 | 0.342105 | 13 | dup-like\_integration | yes |
| HTMCP-03-06-02020 | chr4 | 102429177 | HPV45 | 2979 | hpv.site2 | event1 | 0.372093 | 16 | dup-like\_integration | yes |

#### Summarize samples by number of integration sites/events

Here, the number of integration sites/events are summarized by sample
for an easy reference

```
kable(head(summary))
```

| sample | events | sites | transcribed.events | HPV.type | chromosomes |
| --- | --- | --- | --- | --- | --- |
| HTMCP-03-06-02001 | 1 | 2 | 1 | HPV16 | chr3 |
| HTMCP-03-06-02006 | 1 | 2 | 1 | HPV45 | chr12 |
| HTMCP-03-06-02020 | 1 | 2 | 1 | HPV45 | chr4 |
| HTMCP-03-06-02037 | 1 | 6 | 1 | HPV52 | chr6 |
| HTMCP-03-06-02040 | 1 | 2 | 1 | HPV52 | chr13 |
| HTMCP-03-06-02042 | 1 | 2 | 1 | HPV16 | chr1 |

#### Simplify the data for figures

Figures describing the integration events are created using
simplified versions of these tables. First, to describe the integration
types by HPV type, we will make a simplified HPV type. Events will
either be categorized as HPV16, HPV18, HPV45, or other HPV type.

#### Create a figure for the integration event types from various HPV types

This figure depicts what proportion of events from each HPV type
belong to each integration event category.

```
p <- ggplot(ntypes, aes(x = HPV.type.simp, y = type, size = percent)) +
  geom_point(colour="black",pch=21, fill = "grey") + 
  theme_minimal() +
  scale_size(range = c(2,15)) +
  labs(x = NULL, y = NULL) +
  theme(axis.text = element_text(size = 12, face = "bold", colour = "black"))
ggsave(filename = "figure1/intType_HPVs.pdf", plot = p, height = 5, width = 8, units = "in")
p
```

#### Create a figure for the number of integration types

This figure depicts the number of each integration event type found
across the cohort, and what proportion of the events are expressed (yes
= transcribed, no = not transcribed).

```
## Integration types bar plot
nAll <- nAll %>% arrange(desc(n))
nAll2$type <- factor(nAll2$type, levels = nAll$type)

p1 <- ggplot(nAll2, aes(x = type, y = n, fill = is.event.transcribed)) +
  geom_bar(stat = "identity", colour = "black") + 
  theme_minimal() +
  labs(y = "# of events", x = NULL, fill = NULL) +
  scale_fill_manual(values = c("#8d99ae", "#fb8500"))+
  theme(axis.text.y = element_text(colour = "black", size = 13),
        axis.text.x = element_text(colour = "black", size = 13, angle = 45, hjust = 1, vjust = 1),
        axis.title = element_text(colour = "black", size = 14, face = "bold"),
        legend.text = element_text(size = 12, colour = "black"),
        panel.grid.minor = element_blank(), 
        panel.grid.major.x = element_blank(), 
        axis.ticks.y = element_line(),
        axis.line = element_line())
ggsave(filename = "figure1/intType_num.pdf", plot = p1, height = 3, width = 6, units = "in")
p1
```

#### Test if dup-like events are transcribed more than other events

It appears a higher proportion of dup-like events are transcribed
than other types. We will use a Fisher’s Exact test to see if that is
significant.

```
# Fisher's exact test
fisher.test(matrix(c(nAll2$n[nAll2$type == "dup-like" & nAll2$is.event.transcribed == "yes"],
                     nAll2$n[nAll2$type == "dup-like" & nAll2$is.event.transcribed == "no"],
                     sum(nAll2$n[nAll2$type != "dup-like" & nAll2$is.event.transcribed == "yes"]),
                     sum(nAll2$n[nAll2$type != "dup-like" & nAll2$is.event.transcribed == "no"])), 
                   byrow = T, nrow = 2))
```

```
## 
##  Fisher's Exact Test for Count Data
## 
## data:  matrix(c(nAll2$n[nAll2$type == "dup-like" & nAll2$is.event.transcribed == "yes"], nAll2$n[nAll2$type == "dup-like" & nAll2$is.event.transcribed == "no"], sum(nAll2$n[nAll2$type != "dup-like" & nAll2$is.event.transcribed == "yes"]), sum(nAll2$n[nAll2$type != "dup-like" & nAll2$is.event.transcribed == "no"])), byrow = T, nrow = 2)
## p-value = 0.0009629
## alternative hypothesis: true odds ratio is not equal to 1
## 95 percent confidence interval:
##   1.613428 10.769093
## sample estimates:
## odds ratio 
##    4.00036
```

```
# percent of  events that are transcribed
nAll2$n[nAll2$type == "dup-like" & nAll2$is.event.transcribed == "yes"] / sum(nAll2$n[nAll2$type == "dup-like"])
```

```
## [1] 0.7567568
```

```
nAll2$n[nAll2$type == "del-like" & nAll2$is.event.transcribed == "yes"] / sum(nAll2$n[nAll2$type == "del-like"])
```

```
## [1] 0.5517241
```

```
nAll2$n[nAll2$type == "multi-breakpoint" & nAll2$is.event.transcribed == "yes"] / sum(nAll2$n[nAll2$type == "multi-breakpoint"])
```

```
## [1] 0.4878049
```

```
nAll2$n[nAll2$type == "translocation" & nAll2$is.event.transcribed == "yes"] / sum(nAll2$n[nAll2$type == "translocation"])
```

```
## [1] 0.3333333
```

```
nAll2$n[nAll2$type == "unmatched" & nAll2$is.event.transcribed == "yes"] / sum(nAll2$n[nAll2$type == "unmatched"])
```

```
## [1] 0.125
```

```
nAll2$n[nAll2$type == "repeat" & nAll2$is.event.transcribed == "yes"] / sum(nAll2$n[nAll2$type == "repeat"])
```

```
## [1] 0.3333333
```

```
sum(nAll2$n[nAll2$is.event.transcribed == "yes"])
```

```
## [1] 68
```

```
sum(nAll2$n[nAll2$is.event.transcribed == "no"])
```

```
## [1] 61
```

#### Upset plot across the number of integration event types

This plot depicts the overlap of different integration event types
co-occurring in a sample using an upset plot.

```
## UPSET PLOT DIFF TYPES
p2 <- intOnly %>%
  distinct(sample, type) %>%
  group_by(sample) %>%
  summarise(list = list(type)) %>%
  ggplot(aes(x=list)) +
  geom_bar() +
  scale_x_upset() +
  theme_minimal() +
  theme(axis.text.y = element_text(colour = "black", size = 13),
        axis.title = element_blank(),
        legend.text = element_text(size = 12, colour = "black"),
        panel.grid.minor = element_blank(), 
        panel.grid.major.x = element_blank(), 
        axis.ticks.y = element_line(),
        axis.line = element_line(),
        plot.margin = margin(1,1,1.5,6, "cm"))
ggsave(filename = "figure1/intType_upset.pdf", plot = p2, height = 4, width = 6, units = "in")
p2
```

#### Statistical information about the samples and events

Here we list various statistical numbers for the manuscript to
describe HPV integration in this cohort.

```
## -------------------------------------------------------------------------
## Stats - Fig 1ef
## -------------------------------------------------------------------------

length(unique(int$sample)) # number of samples
```

```
## [1] 72
```

```
length(unique(int$sample[int$type != "no_detected_integration"])) # number of integrated samples
```

```
## [1] 69
```

```
nevents <- length(int$sample[int$type != "no_detected_integration"])
nevents # number of events
```

```
## [1] 129
```

```
nsites <- nrow(sum[sum$integration_type != "no_detected_integration",])
nsites # number of sites
```

```
## [1] 438
```

```
sum(int$type == "multi-breakpoint_integration")/nevents # percent of multi-breakpoint events
```

```
## [1] 0.3178295
```

```
sum(int$type == "multi-breakpoint_integration") # number of multi-breakpoint events
```

```
## [1] 41
```

```
sum(int$type == "del-like_integration") # number of del-like events
```

```
## [1] 29
```

```
sum(int$type == "dup-like_integration") # number of dup-like events
```

```
## [1] 37
```

```
sum(int$type == "translocation_integration") # number of translocation events
```

```
## [1] 3
```

```
sum(int$type == "repeat_integration") # number of repeat events
```

```
## [1] 3
```

```
sum(int$type == "unmatched_integration") # number of unmatched events
```

```
## [1] 16
```

```
summary(factor(intOnly$type[intOnly$HPV.type == "HPV16"])) # types of events by HPV type - HPV16
```

```
##         del-like_integration         dup-like_integration 
##                           14                           12 
## multi-breakpoint_integration           repeat_integration 
##                           20                            1 
##        unmatched_integration 
##                            8
```

```
summary(factor(intOnly$type[intOnly$HPV.type == "HPV18"])) # types of events by HPV type - HPV18
```

```
##         del-like_integration         dup-like_integration 
##                            6                           11 
## multi-breakpoint_integration           repeat_integration 
##                            8                            1 
##    translocation_integration        unmatched_integration 
##                            1                            2
```

```
summary(factor(intOnly$type[intOnly$HPV.type == "HPV45"])) # types of events by HPV type - HPV45
```

```
##         del-like_integration         dup-like_integration 
##                            3                           10 
## multi-breakpoint_integration        unmatched_integration 
##                            1                            1
```

## Meta data for sequenced samples

To introduce the samples, we will tabulate and show all the metadata
for the sequenced samples and also include a summary of the integration
events.

#### Create the metadata figure

```
kable(head(htmcpSum))
```

| sample | events | sites | transcribed.events | HPV.type | chromosomes |
| --- | --- | --- | --- | --- | --- |
| HTMCP-03-06-02001 | 1 | 2 | 1 | HPV16 | chr3 |
| HTMCP-03-06-02006 | 1 | 2 | 1 | HPV45 | chr12 |
| HTMCP-03-06-02020 | 1 | 2 | 1 | HPV45 | chr4 |
| HTMCP-03-06-02037 | 1 | 6 | 1 | HPV52 | chr6 |
| HTMCP-03-06-02040 | 1 | 2 | 1 | HPV52 | chr13 |
| HTMCP-03-06-02042 | 1 | 2 | 1 | HPV16 | chr1 |

```
# number of integration events
a1 <- ggplot(htmcpSum, aes(x = sample, y = events)) +
  geom_bar(stat = "identity", colour = "black", fill = "grey", size = 0.25) +
  theme_minimal() +
  labs(y = "# of events") +
  theme(axis.text.x = element_blank(),
        axis.title.x = element_blank(),
        axis.text.y = element_text(size = 12, colour = "black"),
        axis.title.y = element_text(size = 14, colour = "black", face = "bold"),
        legend.text = element_text(size = 12, colour = "black"),
        legend.title = element_text(size = 14, colour = "black", face = "bold"),
        panel.grid.minor = element_blank(),
        panel.grid.major.x = element_blank(),
        axis.ticks.y = element_line(),
        legend.position = "top") 

# sample information
b1 <- ggplot(anno, aes(y = row, x = Patient, fill = value)) +
  geom_tile(colour = "black") + 
  theme_minimal()+
  scale_fill_manual(values = meta_colours[["values"]]) +
  theme(axis.text.x = element_blank(),
        axis.title = element_blank(),
        axis.text.y = element_text(size = 12, colour = "black"),
        legend.text = element_text(size = 12, colour = "black"),
        legend.title = element_blank(),
        axis.ticks = element_blank(),
        legend.position = "none",
        legend.key.size = unit(0.4, "cm"))

# number of integration breakpoints
c1 <- ggplot(htmcpSum, aes(x = sample, y = transcribed.events)) +
  geom_bar(stat = "identity", colour = "black", fill = "grey", size = 0.25) +
  theme_minimal() +
  scale_y_reverse() +
  labs(y = "# of expressed events") +
  theme(axis.text.x = element_blank(),
        axis.title.x = element_blank(),
        axis.text.y = element_text(size = 12, colour = "black"),
        axis.title.y = element_text(size = 14, colour = "black", face = "bold"),
        legend.text = element_text(size = 12, colour = "black"),
        legend.title = element_text(size = 14, colour = "black", face = "bold"),
        panel.grid.minor = element_blank(),
        panel.grid.major.x = element_blank(),
        axis.ticks.y = element_line(),
        legend.position = "none")

p1 <- plot_grid(a1, b1, c1, align = "v", ncol = 1, rel_heights = c(1,1,0.2))
ggsave(filename = "figure1/htmcp_cohort_hpvInt.pdf", plot = p1, height = 6, width = 9.5, units = "in")

# save tables
write.table(anno, file = "sourceTables/fig1a-1.txt", col.names = T, sep = "\t", row.names = F, quote = F)
write.table(htmcpSum, file = "sourceTables/fig1a-2.txt", col.names = T, sep = "\t", row.names = F, quote = F)
p1
```

#### Compare HIV positive and HIV negative samples

Here, we check to see if HIV status influences the number of HPV
breakpoints in a sample. Unfortunately, the difference is insignificant,
but HIV positive samples trended higher.

```
summary$HIV.status <- htmcpMeta$HIV.status[match(summary$sample, htmcpMeta$Patient)]

p2 <- ggplot(summary, aes(x = HIV.status, y = events, fill = HIV.status)) +
    geom_boxplot(outlier.shape = NA) + 
    geom_jitter(height = 0, width = 0.2, size = 3, alpha=0.5) +
    theme_bw() + 
    scale_fill_manual(values = c(Negative="black", Positive="#FACDBE")) +
    labs(x = "HIV status", y = "# of events", fill = NULL, colour = NULL) +
    theme(axis.text = element_text(colour = "black", size = 12),
          axis.title = element_text(colour = "black", size = 12, face = "bold"),
          legend.text = element_text(size = 12, colour = "black"),
          panel.grid = element_blank(), 
          axis.ticks.y = element_line(),
          axis.line = element_line(),
          legend.position = "none")+
    stat_compare_means(method = "wilcox.test")
ggsave(filename = "figure1/hivStatusNEvents.pdf", plot = p2, height = 3, width = 3, units = "in")
p2
```

#### N values for the sample metadata

```
table(htmcpMeta$HPV.type) # HPV type
```

```
## 
## HPV16 HPV18 HPV45 HPV52 HPV58 HPV59 HPV82 HPV26 HPV31 HPV33 HPV68 
##    25    21    12     4     2     2     2     1     1     1     1
```

```
table(htmcpMeta$HPV.clade) # HPV clade
```

```
## 
##    A9    A7 Other 
##    33    36     3
```

```
table(htmcpMeta$Stage) # Stage
```

```
## 
##   Stage_I  Stage_II Stage_III  Stage_IV 
##         8        39        20         4
```

```
table(htmcpMeta$Final.histology) # histology
```

```
## 
##   Adenocarcinoma    Adenosquamous   Neuroendocrine         Squamous 
##                3                7                1               60 
## Undifferentiated 
##                1
```

```
table(htmcpMeta$Age) # Age
```

```
## 
## A45to65  Over65 Under45 
##      31       4      36
```

```
table(htmcpMeta$HIV.status) # HIV Status
```

```
## 
## Negative Positive 
##       33       39
```

```
table(htmcpMeta$Grade) # Grade
```

```
## 
## G1 G2 G3 
##  3 36 32
```

```
table(htmcpMeta$HRD.score) # HRD Score
```

```
## 
## A10to30  Over30 Under10 
##      46      11      15
```

```
table(htmcpMeta$Max.APOBEC.score) # HRD Score
```

```
## 
## A0.2to0.4   Over0.4  Under0.2 
##        37        13        22
```

```
table(htmcpMeta$Ploidy) # Ploidy
```

```
## 
##  four three   two 
##     5    21    46
```

## The genomic position of HPV integration events (Figure 1)

In this analysis, we are looking to see how the genomic positions of
HPV integrated loci are distributed across the genome. We also want to
test if the distribution is different in expressed and non-expressed
events.

#### Read in the integration events sorted by distance

```
kable(head(dist))
```

| sample | chr | start | end | sites | hpv\_site | event | integration.type | nsites | HPV.type | is.event.transcribed |
| --- | --- | --- | --- | --- | --- | --- | --- | --- | --- | --- |
| HTMCP-03-06-02001 | chr3 | 189823818 | 189825182 | hpv.site1,hpv.site2 | hpv.site1 | event1 | dup-like\_integration | 2 | HPV16 | yes |
| HTMCP-03-06-02006 | chr12 | 45934723 | 45934738 | hpv.site1,hpv.site2 | hpv.site1 | event1 | del-like\_integration | 2 | HPV45 | yes |
| HTMCP-03-06-02020 | chr4 | 102391305 | 102429178 | hpv.site1,hpv.site2 | hpv.site1 | event1 | dup-like\_integration | 2 | HPV45 | yes |
| HTMCP-03-06-02037 | chr6 | 157310357 | 157314365 | hpv.site1,hpv.site2,hpv.site4,hpv.site5,hpv.site6,hpv.site7 | hpv.site1 | event1 | multi-breakpoint\_integration | 6 | HPV52 | yes |
| HTMCP-03-06-02040 | chr13 | 74511383 | 74511402 | hpv.site1,hpv.site2 | hpv.site1 | event1 | del-like\_integration | 2 | HPV52 | yes |
| HTMCP-03-06-02042 | chr1 | 151056158 | 151057019 | hpv.site1,hpv.site2 | hpv.site1 | event1 | del-like\_integration | 2 | HPV16 | yes |

#### Plot the karyogram

```
p1 <- ggplot() +
    # chromosome bars
    geom_segment(data = chromSize %>% filter(chr %in% paste0("chr", 1:12)), aes(x = chr, xend = chr, y = 0, yend = size), 
                 lineend = "round", color = "lightgrey", size = 4) +
    # centromeres
    geom_point(data = centPos %>% filter(chr %in% paste0("chr", 1:12)), aes(x = chr, y = centre), 
               size = 4, colour = "black") +
    # integration types
    geom_rect(data = hsPlot2 %>% filter(chr %in% paste0("chr", 1:12)), 
              aes(xmin = as.integer(chr) + 0.1, xmax = (as.integer(chr) + 0.1 + percMax), ymin = pos, ymax = pos+3, fill = expression),
              size = 1) +
    # expression
    #geom_rect(data = hsPlot2 %>% filter(chr %in% paste0("chr", 1:12)), 
    #          aes(xmax = as.integer(chr) - 0.1, xmin = (as.integer(chr) - 0.1 - percMax), ymin = pos, ymax = pos+3, fill = expression),
    #          size = 1) +
    # legend bars
    geom_rect(data = adL, 
              aes(xmin = xmin, xmax = xmax, ymin = ymin, ymax = ymax),
              fill = "black", size = 0.15) +
    # legend text
    geom_text(data = adW, 
              aes(x = x, y = y, label = label))+
    ylim(0, 250) +
    scale_fill_manual(values = c("#8d99ae", "#fb8500"))+
    #scale_fill_manual(values = c("#118ab2","#ef476f","#ffd166","#8d99ae","#06d6a0", "red","#caadff","black"))+
    theme_classic() +
    theme(text = element_text(size=15),axis.line=element_blank(),
          axis.ticks.x=element_blank())+
    labs(x=NULL,y="Chromosome Size (Mb)")

# very annoying but you have to filter all the dataframes or else the factor levels won't match the integer value
chromSizeFilt <- chromSize %>% filter(chr %in% c(paste0("chr", 13:22), "chrX"))
chromSizeFilt$chr <- factor(chromSizeFilt$chr,levels=c(paste0("chr", 13:22), "chrX"))
centPosFilt <- centPos %>% filter(chr %in% c(paste0("chr", 13:22), "chrX"))
hsPlotFilt <- hsPlot %>% filter(chr %in% c(paste0("chr", 13:22), "chrX"))
hsPlotFilt$chr <- factor(hsPlotFilt$chr,levels=c(paste0("chr", 13:22), "chrX"))
hsPlot2Filt <- hsPlot2 %>% filter(chr %in% c(paste0("chr", 13:22), "chrX"))
hsPlot2Filt$chr <- factor(hsPlot2Filt$chr,levels=c(paste0("chr", 13:22), "chrX"))

p2 <- ggplot() +
    # chromosome bars
    geom_segment(data = chromSizeFilt, aes(x = chr, xend = chr, y = 0, yend = size), 
                 lineend = "round", color = "lightgrey", size = 4) +
    # centromeres
    geom_point(data = centPosFilt, aes(x = chr, y = centre), 
               size = 4, colour = "black") +
    # ASE genes
    geom_rect(data = hsPlot2Filt, 
              aes(xmin = as.integer(chr) + 0.1, xmax = (as.integer(chr) + 0.1 + percMax), ymin = pos, ymax = pos+3, fill = expression),
              size = 1) +
    #geom_rect(data = hsPlot2Filt, 
    #          aes(xmax = as.integer(chr) - 0.1, xmin = (as.integer(chr) - 0.1 - percMax), ymin = pos, ymax = pos+3, fill = expression),
    #          size = 1) +
    ylim(0, 250) +
    scale_fill_manual(values = c("#8d99ae", "#fb8500"))+
    #scale_fill_manual(values = c("#118ab2","#ef476f","#ffd166","#8d99ae","#06d6a0", "red","#caadff","grey"))+
    theme_classic() +
    theme(text = element_text(size=15),axis.line=element_blank(),
          axis.ticks.x=element_blank())+
    labs(x=NULL,y="Chromosome Size (Mb)")

# put them together
plot <- plot_grid(p1, p2, align = "v", axis = "l", nrow = 2)

# save plot
ggsave(plot, filename = "figure1/position_events_karyograph.pdf", width = 10, height = 7, units = "in")
plot
```

## Test expression in genes nearby HPV integration at integration “hotspots”

Across the genome there were three regions that had HPV integrated
multiple times, and this included loci containing TP63, MYC, and
KLF5/KLF12, respectively. We want to test and see if the the expression
of these genes is higher in the integrated samples than in unintegrated
samples.

#### Test for recurrently integrated genes

Here, we are looking for the loci with recurrent (>3) integration
across our samples.

```
## --------------------------------------------------------------------------------------
## Genes with > 3 events nearby
## --------------------------------------------------------------------------------------
# Genes with >3 samples integrated nearby
nint <- sort(table(as.factor(allgenes$gene.id)), decreasing = T)
nint <- nint[nint > 3]
print(nint)
```

```
## 
##   KLF12    BORA   CLDN1  CLDN16    DIS3 FAM243B  IL1RAP  KCNE1B    KLF5     MYC 
##       5       4       4       4       4       4       4       4       4       4 
##    MZT1    P3H2   PIBF1 POU5F1B SMIM11B SMIM34B TMEM207    TP63   TPRG1 
##       4       4       4       4       4       4       4       4       4
```

```
# recurrently integrated loci
multiGenes <- allgenes[allgenes$gene.id %in% names(nint),]
multiLoci <- unique(multiGenes$chr.locus)
print(multiLoci)
```

```
## [1] "13q21.33" "13q22.1"  "21p11.2"  "3q28"     "8q24.21"
```

#### Test expression in recurrently integrated genes

The genes KLF5, TP63, and MYC, all have HPV recurrently integrated
nearby. Here we test the expression levels of integrated samples for
each.

```
## --------------------------------------------------------------------------------------
## Test specific genes
## --------------------------------------------------------------------------------------

#gene <- c("MYC")
gene <- c("KLF5")
test_sample <- unique(gsub("-", ".",allgenes$sample[allgenes$gene.id %in% gene]))
expr_mat <- htmcp_mat_sub

p1 <- expr_mat %>%
  filter(gene.id %in% gene) %>%
  gather(sample, tpm,-gene.id) %>%
  mutate(colour = ifelse(sample %in% test_sample, "test", "others")) %>%
  ggplot(aes(x = colour, y = log10(tpm), colour = colour)) +
    geom_boxplot(outlier.shape = NA, position = "dodge") +
    geom_jitter(aes(colour = colour, x = colour), height = 0, width = 0.2, size =3, alpha=0.5) +
    facet_grid(~ gene.id) +
    stat_compare_means(method = "wilcox.test") +
    labs(x = NULL, y = "log10(TPM)") +
    scale_colour_manual(values = c("grey", "dark red")) +
    theme_minimal() +
    theme(panel.grid = element_blank(), 
          axis.text = element_text(size = 13),
          axis.title = element_text(size=14), 
          axis.ticks.y = element_line(),
          axis.line = element_line(), 
          legend.position = "none")

gene <- c("MYC")
test_sample <- unique(gsub("-", ".",allgenes$sample[allgenes$gene.id %in% gene]))
expr_mat <- htmcp_mat_sub

p2 <- expr_mat %>%
  filter(gene.id %in% gene) %>%
  gather(sample, tpm,-gene.id) %>%
  mutate(colour = ifelse(sample %in% test_sample, "test", "others")) %>%
  ggplot(aes(x = colour, y = log10(tpm), colour = colour)) +
    geom_boxplot(outlier.shape = NA, position = "dodge") +
    geom_jitter(aes(colour = colour, x = colour), height = 0, width = 0.2, size =3, alpha=0.5) +
    facet_grid(~ gene.id) +
    stat_compare_means(method = "wilcox.test") +
    labs(x = NULL, y = "log10(TPM)") +
    scale_colour_manual(values = c("grey", "dark red")) +
    theme_minimal() +
    theme(panel.grid = element_blank(), 
          axis.text = element_text(size = 13),
          axis.title = element_text(size=14), 
          axis.ticks.y = element_line(),
          axis.line = element_line(), 
          legend.position = "none")

gene <- c("TP63")
test_sample <- gsub("-", ".",allgenes$sample[allgenes$gene.id == gene])
expr_mat <- htmcp_mat_sub

p3 <- expr_mat %>%
  filter(gene.id == gene) %>%
  gather(sample, tpm,-gene.id) %>%
  mutate(colour = ifelse(sample %in% test_sample, "test", "others")) %>%
  ggplot(aes(x = colour, y = log10(tpm), colour = colour)) +
  geom_boxplot(outlier.shape = NA, position = "dodge") +
    stat_compare_means(method = "wilcox.test") +
  geom_jitter(aes(colour = colour, x = colour), height = 0, width = 0.2, size =3, alpha=0.5) +
    labs(x = NULL, y = "log10(TPM)") +
  scale_colour_manual(values = c("grey", "dark red")) +
    theme_minimal() +
    theme(panel.grid = element_blank(), 
          axis.text = element_text(size = 13),
          axis.title = element_text(size=14), 
          axis.ticks.y = element_line(),
          axis.line = element_line(), 
          legend.position = "none")

ggsave(plot = p1, filename = paste0("figure1/KLF5_integrated_vs_not_integrated.pdf"), width = 2.7, height = 2.7)
ggsave(plot = p2, filename = paste0("figure1/MYC_integrated_vs_not_integrated.pdf"), width = 2.7, height = 2.7)
ggsave(plot = p3, filename = paste0("figure1/TP63_integrated_vs_not_integrated.pdf"), width = 2.7, height = 2.7)

p1
```

```
p2
```

```
p3
```

## ONT Sequencing Statistics

#### Stats for paper

```
## ---------------------------------------------------------------------------
## Stats
## ---------------------------------------------------------------------------

# coverage
mean(datasubLong$value[datasubLong$variable == "read_length_sum"])/1000000000 # average yield
```

```
## [1] 102.2369
```

```
range(datasubLong$value[datasubLong$variable == "read_length_sum"])/1000000000 # range of yield
```

```
## [1]  52.63144 153.03922
```

```
median(datasubLong$value[datasubLong$variable == "coverage"]) # median coverage
```

```
## [1] 33.89808
```

```
range(datasubLong$value[datasubLong$variable == "coverage"]) # range of  coverage
```

```
## [1] 17.26008 50.18805
```

```
# N50
median(datasubLong$value[datasubLong$variable == "n50"])/1000 # median N50
```

```
## [1] 17.4515
```

```
range(datasubLong$value[datasubLong$variable == "n50"])/1000 # range of N50
```

```
## [1]  8.980 34.102
```

```
# chimera
median(datasubLong$value[datasubLong$variable == "chimerism_prop"], na.rm = TRUE) # median chimeria rate
```

```
## [1] 0.05
```

```
range(datasubLong$value[datasubLong$variable == "chimerism_prop"],na.rm = TRUE) # range chimeria rate
```

```
## [1] 0.010 0.124
```

```
# error_rate_by_qualimap
median(datasubLong$value[datasubLong$variable == "error_rate_by_qualimap"],na.rm = TRUE) # median error rate
```

```
## [1] 0.048
```

```
range(datasubLong$value[datasubLong$variable == "error_rate_by_qualimap"],na.rm = TRUE) # range error rate
```

```
## [1] 0.022 0.084
```

#### make figures

```
## ---------------------------------------------------------------------------
## Make figures
## ---------------------------------------------------------------------------

p <- ggplot(datasubLong, aes(y = value, x = cohort, fill = cohort)) + 
  geom_boxplot(outlier.shape = NA) + 
  geom_jitter(height = 0, width = 0.2, size =2, alpha=0.5) +
  facet_wrap(~ variable, nrow = 1, scales = "free") +
  scale_fill_manual(values = c("#DD4A48", "#C0D8C0")) +
  theme_bw() + 
  labs(x = NULL, y = NULL, fill = NULL, colour = NULL) +
  theme(axis.text.y = element_text(colour = "black", size = 13),
        axis.text.x = element_blank(),
        axis.title = element_text(colour = "black", size = 14),
        legend.text = element_text(size = 12, colour = "black"),
        panel.grid = element_blank(), 
        panel.background = element_rect(fill = "white", colour = "black", size = 0.75),
        axis.ticks.y = element_line(),
        axis.ticks.x = element_blank(),
        strip.text = element_text(size = 12, colour = "black"),
        strip.background = element_rect(fill = "#F5EEDC", colour = "black", size = 0.75))
ggsave(p, file = "supp_figures/suppFig1.pdf", width = 10, height = 3)
p
```

## HPV Integrant Analysis (Figure 2)

In this section, the breakpoint positions on the reads are used to
summarize the integrant structures, as preseneted in Figure 2.

#### Compare the HPV integrant size between HPV16 and HPV18

```
p1 <- ggplot(catC %>% filter(HPV.type %in% c("HPV16", "HPV18")), aes(x = HPV.type, colour = HPV.type, y = max_nHPV))+
    geom_boxplot(outlier.shape = NA) + 
    geom_jitter(height = 0, width = 0.2, size =3, alpha=0.5) +
    theme_minimal() +
    xlab("HPV type") + 
    ylab("max # of HPV genomes in integrant") +
    scale_colour_manual(values = c(ann_colors[["HPV.type"]])) +
    theme(panel.grid = element_blank(), 
          axis.text = element_text(size = 12,colour = "black"),
          axis.title = element_text(size=12, face = "bold", colour = "black"), 
          axis.ticks.y = element_line(),
          axis.line = element_line(), 
          legend.position = "none") +
    stat_compare_means(method = "wilcox")
ggsave(p1, file="figure2/HPV16vsHPV18.pdf", height = 3, width = 3)
p1
```

#### Integration types with heterologous integration

```
catC$category_simple <- ifelse(catC$category %in% c("partial", "full"), "single", "heterologous")
catC$integration_type <- factor(catC$integration_type, levels = c("multi-breakpoint_integration","repeat_integration",
                                                                  "del-like_integration","dup-like_integration",
                                                                  "translocation_integration"))

# integration type
p2 <- ggplot(catC %>% filter(category_simple == "heterologous"), aes(x = integration_type))+
    geom_bar() + 
    theme_minimal() +
    xlab("integration type") + 
    ylab("# of heterologous integrants") +
    #scale_colour_manual(values = c(ann_colors[["HPV.type"]])) +
    theme(panel.grid = element_blank(), 
          axis.text.y = element_text(size = 12,colour = "black"),
          axis.text.x = element_text(size = 12,colour = "black", angle = 60, hjust = 1, vjust = 1),
          axis.title = element_text(size=12, face = "bold", colour = "black"), 
          axis.ticks.y = element_line(),
          axis.line = element_line())
p2
```

```
ggsave(p2, file="figure2/fig2f.pdf", height = 4.5, width = 3.2)
p2
```

#### HPV18 vs Other HPVs

```
catC$all <- "all"
catC$HPV18_vs_all <- ifelse(catC$HPV.type == "HPV18", "HPV18", "Other_HPV")
# integration type
p3 <- ggplot(catC, aes(x = HPV18_vs_all, fill = category_simple))+
    geom_bar(position="fill", colour = "black") + 
    theme_minimal() +
    xlab("integration type") + 
    ylab("% of integrants") +
    labs(fill = NULL) +
    scale_fill_manual(values = c("#219ebc","#ffb703")) +
    theme(panel.grid = element_blank(), 
          axis.text.y = element_text(size = 12,colour = "black"),
          axis.text.x = element_text(size = 12,colour = "black", angle = 60, hjust = 1, vjust = 1),
          legend.text = element_text(size = 12,colour = "black"),
          axis.title = element_text(size=12, face = "bold", colour = "black"), 
          axis.ticks.y = element_line(),
          axis.line = element_line())
ggsave(p3, file="figure2/fig2h.pdf", height = 3.2, width = 4.5)
p3
```

#### Integration types sizes

```
p4 <- ggplot(catC, aes(x = integration_type, y = max_nHPV))+
    geom_boxplot(outlier.shape = NA) + 
    geom_jitter(height = 0, width = 0.2, size =2, alpha=0.5, colour = "dark grey") +
    theme_minimal() +
    xlab("integration type") + 
    ylab("max # of HPV genomes in integrant") +
    #scale_colour_manual(values = c(ann_colors[["HPV.type"]])) +
    theme(panel.grid = element_blank(), 
          axis.text.y = element_text(size = 12,colour = "black"),
          axis.text.x = element_text(size = 12,colour = "black", angle = 60, hjust = 1, vjust = 1),
          axis.title = element_text(size=12, face = "bold", colour = "black"), 
          axis.ticks.y = element_line(),
          axis.line = element_line(), 
          legend.position = "none") 
ggsave(p4, file="figure2/fig2g.pdf", height = 5.5, width = 5.5)
p4
```

#### Number of HPV integrant sizes per breakpoint pair

```
p5 <- ggplot(catC, aes(x = as.factor(ngroups), fill = category_simple))+
    geom_bar(colour = "black") + 
    theme_minimal() +
    xlab("# of integrant structures") + 
    ylab("# of breakpoint pairs") +
    labs(fill = NULL) +
    scale_fill_manual(values = c("#219ebc","#ffb703")) +
    theme(panel.grid = element_blank(), 
          axis.text.y = element_text(size = 12,colour = "black"),
          axis.text.x = element_text(size = 12,colour = "black"),
          legend.text = element_text(size = 12,colour = "black"),
          axis.title = element_text(size=12, face = "bold", colour = "black"), 
          axis.ticks.y = element_line(),
          axis.line = element_line())
ggsave(p5, file="figure2/fig2c.pdf", height = 2.5, width = 6)
p5
```

#### Size of incomplete integrants

```
catI <- cat[cat$status == "incomplete",]

p6 <- ggplot(catI, aes(x = max_length/1000))+
    geom_histogram(colour = "black", size = 0.5) + 
    theme_minimal() +
    xlab("max length of incomplete HPV integrant (bp)") + 
    ylab("count") +
    labs(fill = NULL) +
    theme(panel.grid.minor = element_blank(), 
          axis.text.y = element_text(size = 12,colour = "black"),
          axis.text.x = element_text(size = 12,colour = "black"),
          legend.text = element_text(size = 12,colour = "black"),
          axis.title = element_text(size=12, face = "bold", colour = "black"), 
          axis.ticks.y = element_line(),
          axis.ticks.x = element_line(),
          axis.line = element_line())
ggsave(p6, file="figure2/fig2j.pdf", height = 4, width = 4)
p6
```

#### Stats on integrants

```
### -------------------------------------------------------------------------------
### GET STATS ON THE INTEGRANTS
### -------------------------------------------------------------------------------

# Longest integrant
head(cat %>% arrange(desc(max_nHPV)), 1)
```

```
##              sample bp_pair_name        bp_pair ngroups max_length max_nHPV
## 1 HTMCP-03-06-02176  break-pair7 chr17:81211762       0      48166 6.093877
##       status   category size_category            bp1  event
## 1 incomplete incomplete         over3 chr17:81211762 event3
##       integration_type HPV.type
## 1 del-like_integration    HPV16
```

```
head(cat %>% filter(status == "complete") %>% arrange(desc(max_nHPV)), 1)
```

```
##              sample bp_pair_name                       bp_pair ngroups
## 1 HTMCP-03-06-02175 break-pair44 chr18:68247789_chr18:68297246       1
##   max_length max_nHPV   status category size_category            bp1  event
## 1      36915 4.718175 complete     full         over3 chr18:68247789 event8
##               integration_type HPV.type
## 1 multi-breakpoint_integration    HPV58
```

```
# complete integrants
table(catC$integration_type)
```

```
## 
## multi-breakpoint_integration           repeat_integration 
##                          162                            9 
##         del-like_integration         dup-like_integration 
##                           10                           29 
##    translocation_integration 
##                            2
```

```
table(catC$HPV.type)
```

```
## 
## HPV16 HPV18 HPV26 HPV31 HPV45 HPV52 HPV58 HPV59 HPV68 HPV82 
##    96    55     1     1    13     4    33     4     1     4
```

```
# percent heterologous
table(catC$category)
```

```
## 
##         full heterologous      partial 
##           62           45          105
```

```
table(catC$category_simple)
```

```
## 
## heterologous       single 
##           45          167
```

```
table(catC$HPV18_vs_all)
```

```
## 
##     HPV18 Other_HPV 
##        55       157
```

```
table(catC$category)/sum(table(catC$category))
```

```
## 
##         full heterologous      partial 
##    0.2924528    0.2122642    0.4952830
```

```
fisher.test(matrix(c(nrow(catC[catC$HPV.type != "HPV18" & catC$category == "heterologous",]),
                     nrow(catC[catC$HPV.type == "HPV18" & catC$category == "heterologous",]),
                     nrow(catC[catC$HPV.type != "HPV18" & catC$category != "heterologous",]),
                     nrow(catC[catC$HPV.type == "HPV18" & catC$category != "heterologous",])),byrow = T, ncol=2))
```

```
## 
##  Fisher's Exact Test for Count Data
## 
## data:  matrix(c(nrow(catC[catC$HPV.type != "HPV18" & catC$category == "heterologous", ]), nrow(catC[catC$HPV.type == "HPV18" & catC$category == "heterologous", ]), nrow(catC[catC$HPV.type != "HPV18" & catC$category != "heterologous", ]), nrow(catC[catC$HPV.type == "HPV18" & catC$category != "heterologous", ])), byrow = T, ncol = 2)
## p-value = 0.007183
## alternative hypothesis: true odds ratio is not equal to 1
## 95 percent confidence interval:
##  0.1778355 0.8088972
## sample estimates:
## odds ratio 
##  0.3780415
```

```
# heterologous 
catCH <- catC %>% filter(category == "heterologous")
table(catCH$integration_type)
```

```
## 
## multi-breakpoint_integration           repeat_integration 
##                           41                            2 
##         del-like_integration         dup-like_integration 
##                            0                            2 
##    translocation_integration 
##                            0
```

```
table(catCH$HPV.type)
```

```
## 
## HPV16 HPV18 HPV58 HPV68 HPV82 
##    19    19     5     1     1
```

```
table(catCH$integration_type)/sum(table(catCH$integration_type))
```

```
## 
## multi-breakpoint_integration           repeat_integration 
##                   0.91111111                   0.04444444 
##         del-like_integration         dup-like_integration 
##                   0.00000000                   0.04444444 
##    translocation_integration 
##                   0.00000000
```

```
# heterologous per HPV type
table(catC$category[catC$HPV.type == "HPV16"])/sum(table(catC$category[catC$HPV.type == "HPV16"]))
```

```
## 
##         full heterologous      partial 
##    0.2708333    0.1979167    0.5312500
```

```
table(catC$category_simple[catC$HPV.type == "HPV18"])/sum(table(catC$category_simple[catC$HPV.type == "HPV18"]))
```

```
## 
## heterologous       single 
##    0.3454545    0.6545455
```

```
table(catC$category[catC$HPV.type != "HPV18"])/sum(table(catC$category[catC$HPV.type != "HPV18"]))
```

```
## 
##         full heterologous      partial 
##    0.2929936    0.1656051    0.5414013
```

```
table(catC$category_simple)/sum(table(catC$category_simple))
```

```
## 
## heterologous       single 
##    0.2122642    0.7877358
```

```
# length per HPV type
catC %>%
  group_by(HPV.type) %>%
  summarise(mean = mean(max_nHPV))
```

```
## # A tibble: 10 × 2
##    HPV.type   mean
##    <chr>     <dbl>
##  1 HPV16    1.12  
##  2 HPV18    1.40  
##  3 HPV26    0.150 
##  4 HPV31    0.0626
##  5 HPV45    0.784 
##  6 HPV52    0.829 
##  7 HPV58    1.60  
##  8 HPV59    2.32  
##  9 HPV68    1.35  
## 10 HPV82    1.07
```

```
catC %>%
  group_by(HPV.type) %>%
  summarise(mean = mean(max_nHPV))
```

```
## # A tibble: 10 × 2
##    HPV.type   mean
##    <chr>     <dbl>
##  1 HPV16    1.12  
##  2 HPV18    1.40  
##  3 HPV26    0.150 
##  4 HPV31    0.0626
##  5 HPV45    0.784 
##  6 HPV52    0.829 
##  7 HPV58    1.60  
##  8 HPV59    2.32  
##  9 HPV68    1.35  
## 10 HPV82    1.07
```

```
# number of incomplete breakpoints
table(cat$category)
```

```
## 
##         full heterologous   incomplete      partial 
##           62           45          123          105
```

```
table(cat$HPV.type[cat$category == "incomplete"])
```

```
## 
## HPV16 HPV18 HPV31 HPV45 HPV52 HPV58 HPV59 HPV82 
##    55    22     5     4     3    31     2     1
```

```
# largest integrants
max(cat$max_nHPV[cat$category == "incomplete"])
```

```
## [1] 6.093877
```

```
max(cat$max_length[cat$category == "incomplete"])
```

```
## [1] 48166
```

```
max(catC$max_nHPV)
```

```
## [1] 4.718175
```

```
max(catC$max_length)
```

```
## [1] 36915
```

```
# incomplete integrants
mean(catI$max_nHPV)
```

```
## [1] 2.073212
```

```
mean(catI$max_length)
```

```
## [1] 16308.68
```

```
table(catI$HPV.type)
```

```
## 
## HPV16 HPV18 HPV31 HPV45 HPV52 HPV58 HPV59 HPV82 
##    55    22     5     4     3    31     2     1
```

### HPV Episome Sizes

```
sub <- paf %>%
    select(sample, qname, qlen) %>%
    filter(!duplicated(qname))

p <- ggplot(sub, aes(x = qlen)) +
    geom_vline(xintercept = 7900, colour = "red", linetype = 2, alpha = 0.5)+
    geom_vline(xintercept = 15800, colour = "red", linetype = 2, alpha = 0.5) +
    geom_vline(xintercept = 23700, colour = "red", linetype = 2, alpha = 0.5)+
    geom_vline(xintercept = 31600, colour = "red", linetype = 2, alpha = 0.5)+
    geom_histogram(binwidth = 100, colour = "black") +
    facet_wrap(sample ~ ., scales = "free_y", ncol = 2) +
    xlim(0,60000) +
    theme_bw() +
    theme(axis.text = element_text(size = 11, colour = "black"),
          axis.title = element_text(size = 13, colour = "black", face = "bold"))
pg <- ggplot_build(p)
p_df <- pg[["data"]][[5]]
ggsave(filename = "figure2/fig2e_episome_sizes.pdf", plot = p, height = 4, width = 7.5)
p
```

## Two-breakpoint events (del-like and dup-like, Figure 3)

```
summaryTwo <- summary1[summary1$n == 2,]
summaryTwo$dist <- NA

for (i in summaryTwo$sample) {
  for (j in summaryTwo$event) {
    breaks <- sum$pos[sum$sample == i & sum$event == j]
    distance <- max(breaks) - min(breaks)
    summaryTwo$dist[summaryTwo$sample == i & summaryTwo$event == j] <- distance
  }
}
summaryTwo$intType <- intOnly$type[match(paste0(summaryTwo$sample, summaryTwo$event), paste0(intOnly$sample, intOnly$event))]
summaryTwo_sub <- summaryTwo[summaryTwo$intType %in% c("dup-like_integration", "del-like_integration"),]
summaryTwo_sub$intType <- gsub("_integration", "", summaryTwo_sub$intType)

# stat test
wilcox.test(dist ~ intType,
             data = summaryTwo_sub)
```

```
## 
##  Wilcoxon rank sum test with continuity correction
## 
## data:  dist by intType
## W = 189.5, p-value = 7.561e-06
## alternative hypothesis: true location shift is not equal to 0
```

```
table(summaryTwo_sub$intType)
```

```
## 
## del-like dup-like 
##       29       37
```

#### eccDNA

Show the sizes of the potential eccDNAs

```
kable(ecOnly)
```

| id | sample | event | V1 | size | nreads | integration\_type | HPV.type | is.event.transcribed |
| --- | --- | --- | --- | --- | --- | --- | --- | --- |
| HTMCP-03-06-02040/event1 | HTMCP-03-06-02040 | event1 | ecDNA\_detected | 77999 | 8 | del-like\_integration | HPV52 | yes |
| HTMCP-03-06-02047/event1 | HTMCP-03-06-02047 | event1 | ecDNA\_detected | 60274 | 24 | dup-like\_integration | HPV18 | yes |
| HTMCP-03-06-02058/event1 | HTMCP-03-06-02058 | event1 | ecDNA\_detected | 56792 | 18 | multi-breakpoint\_integration | HPV18 | yes |
| HTMCP-03-06-02170/event1 | HTMCP-03-06-02170 | event1 | ecDNA\_detected | 58906 | 16 | dup-like\_integration | HPV45 | yes |
| HTMCP-03-06-02182/event1 | HTMCP-03-06-02182 | event1 | ecDNA\_detected | 38792 | 151 | dup-like\_integration | HPV18 | yes |
| HTMCP-03-06-02185/event1 | HTMCP-03-06-02185 | event1 | ecDNA\_detected | 52618 | 75 | dup-like\_integration | HPV18 | yes |
| HTMCP-03-06-02195/event3 | HTMCP-03-06-02195 | event3 | ecDNA\_detected | 22440 | 6 | dup-like\_integration | HPV45 | no |
| HTMCP-03-06-02242/event1 | HTMCP-03-06-02242 | event1 | ecDNA\_detected | 22644 | 72 | dup-like\_integration | HPV45 | yes |

```
pEcSize <- ggplot(ecOnly, aes(x = size)) +
    geom_histogram() + 
    #geom_vline(aes(xintercept = size), linetype = 2) + 
    theme_minimal() +
    xlab("size of eccDNA (bp)") +
    xlim(0,100000)+
    #scale_colour_manual(values = c(ann_colors[["HPV.type"]])) +
    theme(panel.grid = element_blank(), 
          axis.text.y = element_text(size = 12,colour = "black"),
          axis.text.x = element_text(size = 12,colour = "black", angle = 60, hjust = 1, vjust = 1),
          axis.title = element_text(size=12, face = "bold", colour = "black"), 
          axis.ticks.y = element_line(),
          axis.line = element_line(), 
          legend.position = "none") 
pEcSize
```

```
ggsave(filename = "figure3/eccDNA_sizes.pdf", plot = pEcSize, height = 3, width = 5)
```

#### The del-like vs. dup-like distances

```
## figure
p3 <- ggplot(summaryTwo_sub, aes(x = intType, y = dist, fill = intType)) +
  geom_boxplot(outlier.shape = NA) + 
  geom_jitter(height = 0, width = 0.2, size = 3, alpha=0.5) +
  theme_bw() + 
  scale_fill_manual(values = c("#DD4A48", "#C0D8C0", "#f5eedc")) +
  scale_y_log10() +
  labs(x = "Two breakpoint integration types", y = "log10(distance between breakpoints)", fill = NULL, colour = NULL) +
  theme(axis.text = element_text(colour = "black", size = 12),
        axis.title = element_text(colour = "black", size = 12, face = "bold"),
        legend.text = element_text(size = 12, colour = "black"),
        panel.grid = element_blank(), 
        axis.ticks.y = element_line(),
        axis.line = element_line(),
        legend.position = "none")+
  stat_compare_means(method = "wilcox.test")
ggsave(filename = "figure3/twoBreakBoxplot.pdf", plot = p3, height = 5, width = 5, units = "in")
p3
```

#### Regional differences between del-like and dup-like

```
## -------------------------------------------------------------------------
## genic region of two-break events
## -------------------------------------------------------------------------

dir1 <- "/path/to/htmcp/call_integration/output/"
files1 <- grep("genic_test/event_location.txt",list.files(dir1, recursive = T), value = T)
name1 <- gsub("intType/", "", files1)
name1 <- gsub("/genic_test/event_location.txt", "", name1)

reg1 <- NULL
for (i in 1:length(files1)) {
  reg1[[i]] <- read.delim(paste0(dir1, files1[i]), header = F)
}
names(reg1) <- name1
reg1 <- bind_rows(reg1, .id = "event")
reg <- reg1
reg <- reg %>% separate(event, c("sample", "event"), sep = "/")

# put together with the summaryTwo
summaryTwo_sub$region <- reg$V1[match(paste0(summaryTwo_sub$sample, summaryTwo_sub$event), paste0(reg$sample, reg$event))]

## figure
p4 <- ggplot(summaryTwo_sub, aes(x= region,  group=intType)) + 
  geom_bar(aes(y = ..prop.., fill = factor(..x..)), stat="count", colour = "black") +
  geom_text(aes( label = scales::percent(..prop..),
                 y= ..prop.. ), stat= "count", vjust = -.5) +
  labs(y = "% of events", fill=NULL, x = "genomic region") +
    scale_fill_manual(values = c("#f5cac3", "#84a59d", "#f28482")) +
  theme_minimal()+
  facet_grid(~intType) +
  scale_y_continuous(labels = scales::percent) +
  theme(axis.text.y = element_text(colour = "black", size = 12),
        axis.text.x = element_text(colour = "black", size = 12, hjust = 1, angle = 45),
        axis.title = element_text(colour = "black", size = 12, face = "bold"),
        strip.text = element_text(colour = "black", size = 12, face = "bold"),
        legend.text = element_text(size = 12, colour = "black"),
        panel.grid = element_blank(), 
        axis.ticks.y = element_line(),
        axis.line = element_line(),
        legend.position = "none")
ggsave(filename = "figure3/twoBreakEventRegion.pdf", plot = p4, height = 4, width = 7, units = "in")
p4
```

```
## p value test between ecDNA vs. deletions/duplications
mat <- summaryTwo_sub %>%
  group_by(intType, region) %>%
  summarise(n = n())

## save table
write.table(summaryTwo_sub, file = "tables/twoBreakIntegrationCharacteristics.txt", quote = F, sep = "\t", col.names = T, row.names = F)

kable(head(summaryTwo_sub))
```

| sample | event | n | dist | intType | region |
| --- | --- | --- | --- | --- | --- |
| HTMCP-03-06-02001 | event1 | 2 | 1363 | dup-like | genic |
| HTMCP-03-06-02006 | event1 | 2 | 14 | del-like | genic |
| HTMCP-03-06-02020 | event1 | 2 | 37872 | dup-like | genic |
| HTMCP-03-06-02040 | event1 | 2 | 18 | del-like | genic |
| HTMCP-03-06-02042 | event1 | 2 | 860 | del-like | genic |
| HTMCP-03-06-02047 | event1 | 2 | 53038 | dup-like | genic |

```
# n vals
table(summaryTwo_sub$intType)
```

```
## 
## del-like dup-like 
##       29       37
```

```
mat <- as.data.frame(spread(mat, key = intType, value = n))
rownames(mat) <- mat$region
mat <- as.matrix(mat[,-1])
fisher.test(mat)
```

```
## 
##  Fisher's Exact Test for Count Data
## 
## data:  mat
## p-value = 0.04045
## alternative hypothesis: two.sided
```

## Multi-breakpoint event summary and assembly

#### Plot the connections

```
plot_bp_connections(event_id = "HTMCP-03-06-02238/event2")
plot_bp_connections(event_id = "HTMCP-03-06-02175/event2")
plot_bp_connections(event_id = "HTMCP-03-06-02058/event1")
plot_bp_connections(event_id = "HTMCP-03-06-02267/event1")
plot_bp_connections(event_id = "HTMCP-03-06-02210/event1")
plot_bp_connections(event_id = "HTMCP-03-06-02128/event2")    
plot_bp_connections(event_id = "HTMCP-03-06-02149/event1")   
plot_bp_connections(event_id = "HTMCP-03-06-02428/event1")
```

## Multi-breakpoint events

#### figures

```
ann_colors <- list(HPV.clade = c(A7="#B55F8F", A9="#253083", Other="#A9A9A9"),
                   HPV.type = c(HPV16="#3953A4", HPV18="#9768ad", HPV45="#CC138C", HPV82="#369797",
                                HPV52="#0B8DCD", HPV31="#d2ecf9", HPV73="#737474", HPV68="#f3b2d4", HPV97="black", HPV58="#8bafba", HPV59="#ae3030"),
                   is.event.transcribed = c(yes = "#f6851f", no = "#8e9aae"))
p1 <- nbreaks %>% 
    filter(SV_pair == "human_HPV") %>%
    ggplot(aes(x = is.event.transcribed, y = n_breaks, colour = is.event.transcribed)) +
    geom_boxplot(outlier.shape = NA) + 
    geom_jitter(height = 0, width = 0.2, size =3, alpha=0.5) +
    theme_minimal() +
    xlab("HPV transcription status") + 
    ylab("# of HPV breakpoint in event") +
    scale_colour_manual(values = c(ann_colors[["is.event.transcribed"]])) +
    theme(panel.grid = element_blank(), 
          axis.text = element_text(size = 12,colour = "black"),
          axis.title = element_text(size=12, face = "bold", colour = "black"), 
          axis.ticks.y = element_line(),
          axis.line = element_line(), 
          legend.position = "none") +
    stat_compare_means(method = "wilcox")
ggsave(p1, filename = "figure4/nbreaks_transcription.pdf", height = 2.7, width = 2.7, units = "in")
p1
```

```
mean(nbreaks$n_breaks[nbreaks$is.event.transcribed == "yes" & nbreaks$SV_pair == "human_HPV"])
```

```
## [1] 8.35
```

```
mean(nbreaks$n_breaks[nbreaks$is.event.transcribed == "no" & nbreaks$SV_pair == "human_HPV"])
```

```
## [1] 4.571429
```

```
# find unique colours for the samples
n <- length(unique(nbreaksAnno$value[nbreaksAnno$variable == "sample"]))
qual_col_pals = brewer.pal.info[brewer.pal.info$category == 'qual',]
col_vector = unlist(mapply(brewer.pal, qual_col_pals$maxcolors, rownames(qual_col_pals)))
col = sample(col_vector, n)
names(col) <- unique(mb$sample)

# order the anno data
nbreaksAnno$event.id <- factor(nbreaksAnno$event.id, levels = levels(nbreaks$event.id))
nbreaksAnno <- nbreaksAnno %>% arrange(event.id)
    
meta_colours <- list(values = c(A7="#B55F8F", A9="#253083", Other="#A9A9A9",
                                HPV16="#3953A4", HPV18="#9768ad", HPV45="#CC138C", HPV82="#323636", HPV33="#2ba8be", 
                                HPV52="#0B8DCD", HPV31="#d2ecf9", HPV73="#737474", HPV68="#f3b2d4", HPV97="black", HPV58="#8bafba", HPV59="#ae3030", yes="#f6851f", no="#8e9aae", col))

# sample information
p1 <- ggplot(nbreaksAnno, aes(y = variable, x = event.id, fill = value)) +
    geom_tile(colour = "black") + 
    theme_minimal()+
    scale_fill_manual(values = meta_colours[["values"]]) +
    theme(axis.text.x = element_blank(),
          axis.title = element_blank(),
          axis.text.y = element_text(size = 12, colour = "black"),
          legend.text = element_text(size = 12, colour = "black"),
          legend.title = element_blank(),
          axis.ticks = element_blank(),
          panel.grid.major.x = element_blank(),
          legend.position = "none",
          legend.key.size = unit(0.4, "cm"))

p2 <- ggplot(nbreaks, aes(y = n_breaks, x = event.id, fill = SV_pair)) +
    geom_bar(stat = "identity") +
    theme_minimal() +
    labs(y = "# of breakpoints in the event", x = NULL) +
    scale_fill_manual(values = c("#457b9d","#e63946")) +
    theme(axis.line.x.bottom = element_line(),
          axis.line.y.left = element_line(),
          axis.ticks.y = element_line(),
          panel.grid.major.x = element_blank(),
          panel.grid.minor.x = element_blank(),
          axis.text.y = element_text(size = 12, colour = "black"),
          axis.title = element_text(size = 14, colour = "black"),
          axis.text.x = element_blank(),
          axis.ticks.x = element_blank(),
          legend.position = "none")

plot <- plot_grid(p2, p1, rel_heights = c(3,1), ncol = 1, align = "v", axis = "l")
ggsave(plot, filename = "figure4/numSVBreakpointsEvent.pdf", height = 4, width = 7, units = "in")
plot
```

```
## --------------------------------------------
# CORRELATION PLOT
## --------------------------------------------

nbreaks2 <- spread(nbreaks, SV_pair, n_breaks)
nbreaks2$human[is.na(nbreaks2$human)] <- 0

plot2 <- ggscatter(nbreaks2, x = "human_HPV", y = "human",
                    add = "reg.line",  # Add regressin line
                    add.params = list(color = "#457b9d", fill = "lightgray"), # Customize reg. line
                    conf.int = TRUE, alpha = 0.5) + stat_cor(method = "spearman") +
    labs(x = "# of HPV-human breakpoints", y = "# of human SV breakpoints")
ggsave(plot2, filename = "figure4/hpvSVCorrelation.pdf", height = 3.3, width = 3.3, units = "in")

# save table
write.table(nbreaks2,"tables/multi-breakpoint_svbreaks.txt", sep = "\t", col.names = T, row.names = F, quote = F)

plot2
```

## HPV Adjacent Methylation

#### Clean up the dataframe for figures

#### Plot methylation beside HPV

```
### ----------------------------------------------------------
### PLOT THE METHYLATION DATA ADJACENT TO HPV INTEGRATION
### ----------------------------------------------------------

mrAve$is.event.transcribed <- sum$is.event.transcribed[match(mrAve$id, paste0(sum$sample, "/", sum$event))]

mrPlots <- lapply(c("del-like_integration","dup-like_integration"), function(t){
  # Filter mrAve data for ecDNA and deletion integration types
  mrAveSub <- mrAve %>% filter(integration.type == t)
  
  # Order and factor the id variable for ecDNA data
  order <- mrAveSub %>% 
    dplyr::filter(bins %in% allbins[11:13]) %>% 
    dplyr::group_by(id, is.event.transcribed, hpv.type) %>%
    dplyr::summarise(ave = mean(average_methyl)) %>%
    dplyr::arrange(hpv.type, is.event.transcribed, ave) %>% 
    dplyr::distinct(id)
  mrAveSub$id <- factor(mrAveSub$id, levels = order$id)
  
  # Remove NA values for id variable
  mrAveSub <- mrAveSub[!is.na(mrAveSub$id) & !is.na(mrAveSub$bins),]
  
  # Create a plot for data
  plot <- ggplot(mrAveSub, aes(x = bins, y = id, fill = average_methyl)) +
    geom_tile() +
    scale_fill_distiller(palette = "RdBu") +
    theme_bw() +
    facet_grid(hpv.type ~ ., scales = "free", space = "free") +
    labs(fill = "average Methylation", y = "events", x = "distance from HPV integration") +
    theme(axis.text.x = element_text(angle = 60, hjust = 0.5, vjust = 0.5,size = 12, colour = "black"),
          axis.title = element_text(size = 14, face = "bold", colour = "black"),
          panel.grid.major.y = element_blank())
  return(list(order,plot))
}
)

mrPlots2 <- lapply(c("yes","no"), function(t){
    # Filter mrAve data for ecDNA and deletion integration types
    mrAveSub <- mrAve %>% filter(is.event.transcribed == t & grepl("HTMCP", id)) 
    
    # Order and factor the id variable for ecDNA data
    order <- mrAveSub %>% 
        dplyr::filter(bins %in% allbins[11:13]) %>% 
        dplyr::group_by(id, integration.type) %>%
        dplyr::summarise(ave = mean(average_methyl)) %>%
        dplyr::arrange(integration.type, ave) %>% 
        dplyr::distinct(id)
    mrAveSub$id <- factor(mrAveSub$id, levels = order$id)
    
    # Remove NA values for id variable
    mrAveSub <- mrAveSub[!is.na(mrAveSub$id) & !is.na(mrAveSub$bins),]
    
    # Create a plot for data
    plot <- ggplot(mrAveSub, aes(x = bins, y = id, fill = average_methyl)) +
        geom_tile() +
        scale_fill_distiller(palette = "RdBu") +
        theme_bw() +
        facet_grid(integration.type ~ ., scales = "free", space = "free") +
        labs(fill = "average Methylation", y = "events", x = "distance from HPV integration") +
        theme(axis.text.x = element_text(angle = 60, hjust = 0.5, vjust = 0.5,size = 12, colour = "black"),
              axis.title = element_text(size = 14, face = "bold", colour = "black"),
              panel.grid.major.y = element_blank())
    return(list(order,plot))
}
)
mrPlots
```

```
## [[1]]
## [[1]][[1]]
## # A tibble: 29 × 2
## # Groups:   id, is.event.transcribed [29]
##    id                       is.event.transcribed
##    <chr>                    <chr>               
##  1 HTMCP-03-06-02109/event6 no                  
##  2 HTMCP-03-06-02063/event2 no                  
##  3 HTMCP-03-06-02109/event1 no                  
##  4 HTMCP-03-06-02176/event3 no                  
##  5 HTMCP-03-06-02097/event1 no                  
##  6 HTMCP-03-06-02147/event4 no                  
##  7 HTMCP-03-06-02042/event1 yes                 
##  8 HTMCP-03-06-02217/event1 yes                 
##  9 HTMCP-03-06-02063/event1 yes                 
## 10 HTMCP-03-06-02332/event1 yes                 
## # ℹ 19 more rows
## 
## [[1]][[2]]
```

```
## 
## 
## [[2]]
## [[2]][[1]]
## # A tibble: 33 × 2
## # Groups:   id, is.event.transcribed [33]
##    id                       is.event.transcribed
##    <chr>                    <chr>               
##  1 HTMCP-03-06-02411/event9 no                  
##  2 HTMCP-03-06-02109/event5 no                  
##  3 HTMCP-03-06-02147/event5 no                  
##  4 HTMCP-03-06-02411/event7 no                  
##  5 HTMCP-03-06-02332/event2 yes                 
##  6 HTMCP-03-06-02209/event1 yes                 
##  7 HTMCP-03-06-02333/event1 yes                 
##  8 HTMCP-03-06-02155/event1 yes                 
##  9 HTMCP-03-06-02239/event1 yes                 
## 10 HTMCP-03-06-02047/event1 yes                 
## # ℹ 23 more rows
## 
## [[2]][[2]]
```

#### Methylation across the HPV genome

```
### ----------------------------------------------------------
### PLOT METHYLATION ACROSS THE HPV GENOME
### ----------------------------------------------------------

# get the order for the ecDNA and deletion plots
delOrder <- as.data.frame(mrPlots[[1]][1])[,1]
dupOrder <- as.data.frame(mrPlots[[2]][1])[,1]

# Add the event categories and other info to the methyl regions
eventMethHPV$id <- paste0(eventMethHPV$sample, "/", eventMethHPV$event)
eventMethHPV$integration.type <- summary$type[match(eventMethHPV$id, summary$id)]

# match the unintegrated samples
hpvMeth <- hpvMeth[,colnames(eventMethHPV)]

# add to the table 
eventMethHPV <- rbind(eventMethHPV,hpvMeth)

# define the integration types
types <- c("del-like_integration","dup-like_integration","translocation_integration","no_integration_detected")

# plot the HPV methylation for each integration type
hpvPlot <- lapply(types, function(type) {
  if(type == "del-like_integration"){
    eventMethHPV$id = factor(eventMethHPV$id,levels = delOrder)
  } else if (type == "dup-like_integration") {
    eventMethHPV$id = factor(eventMethHPV$id,levels = dupOrder)
  }
  
  ggplot(eventMethHPV %>% dplyr::arrange(chromosome) %>% filter(integration.type == type & !is.na(id)), 
         aes(x = E6.start, y = id, colour = perc.methylated)) +
    geom_point(size = 2) +
    facet_grid(chromosome ~ integration.type, scales = "free", space = "free") +
    theme_bw() + 
    geom_vline(xintercept = 0, linetype = 2, size = 1) +
    scale_color_distiller(palette = "RdBu") + 
    labs(x = "adjusted position", y = "sample", colour = "% methylated") +
    theme(axis.text.x = element_text(colour = "black", size = 11),
          #axis.text.y = element_blank(), 
          axis.title = element_text(colour = "black", size = 12, face = "bold"),
          legend.text = element_text(size = 10, colour = "black"),
          legend.title = element_text(size = 12, colour = "black"),
          panel.grid.minor = element_blank(), 
          axis.ticks.y = element_blank(),
          axis.line = element_line())
})
hpvPlot
```

```
## [[1]]
```

```
## 
## [[2]]
```

```
## 
## [[3]]
```

```
## 
## [[4]]
```

## Transcription and Local Methylation

#### Transcription/methylation plots

```
give.n <- function(x){
    return(c(y = median(x)*1.05, label = length(x))) 
    # experiment with the multiplier to find the perfect position
}

kable(head(tnscpt))
```

| chr | start | stop | region | id | HPVdir | integration.type | hpv.type | sample | position | zscore | rpkm | mean | median | foldchange | aveMethyl | methyl\_status | is.transcribed |
| --- | --- | --- | --- | --- | --- | --- | --- | --- | --- | --- | --- | --- | --- | --- | --- | --- | --- |
| chr12 | 45929723 | 45934723 | 5\_upstream | HTMCP-03-06-02006/event1 | forward | del-like\_integration | HPV45 | HTMCP-03-06-02006 | upstream | 2.001386 | 0.4624019 | 0.2433778 | 0.2320035 | 1.993082e+00 | 0.5361964 | methylated | yes |
| chr12 | 45934737 | 45939737 | 3\_downstream | HTMCP-03-06-02006/event1 | forward | del-like\_integration | HPV45 | HTMCP-03-06-02006 | downstream | 8.342576 | 4.3574019 | 0.1132806 | 0.0389023 | 1.120088e+02 | 0.4479789 | methylated | yes |
| chr4 | 102391305 | 102396305 | 5\_downstream | HTMCP-03-06-02020/event1 | forward | dup-like\_integration | HPV45 | HTMCP-03-06-02020 | upstream | 8.348043 | 0.0576016 | 0.0009945 | 0.0000039 | 1.471474e+04 | 0.9192602 | demethylated | yes |
| chr4 | 102424177 | 102429177 | 3\_upstream | HTMCP-03-06-02020/event1 | forward | dup-like\_integration | HPV45 | HTMCP-03-06-02020 | downstream | 8.366375 | 3.4258016 | 0.0558139 | 0.0060019 | 5.707818e+02 | 0.1423363 | demethylated | yes |
| chr13 | 74506383 | 74511383 | 5\_upstream | HTMCP-03-06-02040/event1 | reverse | del-like\_integration | HPV52 | HTMCP-03-06-02040 | downstream | 8.367429 | 20.4860027 | 0.2847528 | 0.0000030 | 6.865206e+06 | 0.2494959 | demethylated | yes |
| chr13 | 74511401 | 74516401 | 3\_downstream | HTMCP-03-06-02040/event1 | reverse | del-like\_integration | HPV52 | HTMCP-03-06-02040 | upstream | 7.970789 | 0.0060027 | 0.0002028 | 0.0000032 | 1.893934e+03 | 0.8326694 | demethylated | yes |

```
tplot1 <- ggplot(tnscpt %>% filter(position == "downstream"), aes(x = methyl_status, y = log2(foldchange), fill = methyl_status)) +
    geom_boxplot(outlier.shape = NA) + 
    geom_jitter(height = 0, width = 0.2, size =3, alpha=0.5) +
    #facet_grid(~ integration.type) +
    theme_minimal() +
    scale_fill_manual(values = c("#2166AC","#B2182B"))+
    xlab("methylation status") + 
    ylab("log2(sample/median)") +
    theme(panel.grid.minor = element_blank(), 
          axis.text = element_text(size = 13, colour = "black"),
          axis.title = element_text(size=14, face = "bold"), 
          axis.ticks.y = element_line(),
          axis.line = element_line(), 
          legend.position = "none") +
  stat_compare_means(method = "wilcox.test")
ggsave(tplot1, filename = "figure5/methylvsdemethylFC.pdf", width = 3, height = 3, units = "in")
tplot1
```

```
tplot2 <- ggplot(tnscpt %>% filter(position == "downstream"), aes(x = methyl_status, y = log10(rpkm), fill = methyl_status)) +
    geom_boxplot(outlier.shape = NA) + 
    geom_jitter(height = 0, width = 0.2, size =3, alpha=0.5) +
    theme_minimal() +
    scale_fill_manual(values = c("#2166AC","#B2182B"))+
    xlab("methylation status") + 
    ylab("log10(RPKM)") +
    stat_summary(fun.data = give.n, geom = "text", fun.y = median, 
                 position = position_dodge(width = 0.75)) +
    theme(panel.grid.minor = element_blank(), 
          axis.text = element_text(size = 13, colour = "black"),
          axis.title = element_text(size=14, face = "bold"), 
          axis.ticks.y = element_line(),
          axis.line = element_line(), 
          legend.position = "none") +
    stat_compare_means(method = "wilcox.test")
ggsave(tplot2, filename = "figure5/methylvsdemethylRPKM.pdf", width = 3, height = 3, units = "in")
tplot2
```

```
tplot3 <- ggplot(tnscpt  %>% filter(position == "downstream"), aes(x = is.transcribed, y = log10(rpkm), fill = is.transcribed)) +
    geom_boxplot(outlier.shape = NA) + 
    geom_jitter(height = 0, width = 0.2, size =3, alpha=0.5) +
    theme_minimal() +
    scale_fill_manual(values = c("#8d99ae", "#fb8500"))+
    xlab("HPV transcription status") + 
    ylab("log10(RPKM)") +
    stat_summary(fun.data = give.n, geom = "text", fun.y = median, 
                 position = position_dodge(width = 0.75)) +
    theme(panel.grid.minor = element_blank(), 
          axis.text = element_text(size = 13, colour = "black"),
          axis.title = element_text(size=14, face = "bold"), 
          axis.ticks.y = element_line(),
          axis.line = element_line(), 
          legend.position = "none") +
    stat_compare_means(method = "wilcox.test")
ggsave(tplot3, filename = "figure5/transcribedvsnotRPKM.pdf", width = 3, height = 3, units = "in")
tplot3
```

```
tplot4 <- ggplot(tnscpt  %>% filter(position == "downstream"), aes(x = is.transcribed, y = aveMethyl, fill = is.transcribed)) +
    geom_boxplot(outlier.shape = NA) + 
    geom_jitter(height = 0, width = 0.2, size =3, alpha=0.5) +
    theme_minimal() +
    scale_fill_manual(values = c("#8d99ae", "#fb8500"))+
    xlab("HPV transcription status") + 
    ylab("average methylation (<2500bp)") +
    stat_summary(fun.data = give.n, geom = "text", fun.y = median, 
                 position = position_dodge(width = 0.75)) +
    theme(panel.grid.minor = element_blank(), 
          axis.text = element_text(size = 13, colour = "black"),
          axis.title = element_text(size=14, face = "bold"), 
          axis.ticks.y = element_line(),
          axis.line = element_line(), 
          legend.position = "none") +
    stat_compare_means(method = "wilcox.test")
ggsave(tplot4, filename = "figure5/transcribedvsnotMETHYL.pdf", width = 3, height = 3, units = "in")
tplot4
```

```
tplot5 <- ggplot(tnscpt %>% filter(position == "downstream"), aes(x = aveMethyl, y = log10(rpkm))) +
    geom_point(size =3, alpha=0.5, aes(colour = is.transcribed)) + 
    geom_smooth(method='lm') +
    scale_colour_manual(values = c("#8d99ae", "#fb8500"))+
    theme_bw() +
    xlab("methylation status") + 
    ylab("log10(RPKM)") +
    theme(panel.grid.minor = element_blank(), 
          axis.text = element_text(size = 13, colour = "black"),
          axis.title = element_text(size=14, face = "bold"), 
          axis.ticks.y = element_line(),
          axis.line = element_line(), 
          legend.position = "none") +
    stat_cor(method = "pearson", label.x=0.45)
ggsave(tplot5, filename = "figure5/transcriptvsmethyl_corr.pdf", width = 3, height = 3, units = "in")
tplot5
```

```
tplot6 <- ggplot(tnscpt, aes(x = position, y = log10(rpkm), fill = position)) +
    geom_boxplot(outlier.shape = NA) + 
    geom_jitter(height = 0, width = 0.2, size =3, alpha=0.5) +
    theme_minimal() +
    facet_wrap(~ methyl_status)+
    scale_fill_manual(values = c("#2166AC","#B2182B"))+
    xlab("position relative to HPV transcription") + 
    ylab("log10(RPKM)") +
    stat_summary(fun.data = give.n, geom = "text", fun.y = median, 
                 position = position_dodge(width = 0.75)) +
    theme(panel.grid.minor = element_blank(), 
          axis.text = element_text(size = 13, colour = "black"),
          axis.title = element_text(size=14, face = "bold"), 
          axis.ticks.y = element_line(),
          axis.line = element_line(), 
          legend.position = "none") +
    stat_compare_means(method = "wilcox.test")
ggsave(tplot6, filename = "figure5/upvsdownRPKM.pdf", width = 5, height = 3, units = "in")
tplot6
```

```
ehpvH <- ehpv[paste0(ehpv$sample, "/", ehpv$event) %in% tnscpt$id,]

distplot <- ggplot(ehpvH, aes(x=distance/1000, fill = is.max.site)) +
    geom_histogram(bins = 50) +
    facet_grid(is.max.site ~ ., scales = "free_y") +
    theme_minimal() +
    scale_fill_manual(values = c("#fb8500","#8d99ae"))+
    xlab("distance from HPV (kb)") + 
    ylab("number of RNA HPV/human junctions") +
    theme(axis.text = element_text(size = 13, colour = "black"),
          axis.title = element_text(size=14, face = "bold"), 
          axis.ticks.y = element_line(),
          axis.line = element_line(), 
          legend.position = "none") 
ggsave(distplot, filename = "figure5/transcribedDistHist.pdf", width = 3, height = 3, units = "in")
distplot
```

## Allelic methylation at HPV integration events

#### DMR hotspot size

```
pSize <- ggplot(dmrSizeDF, aes(y = DMR.size, x = test)) +
  geom_violin() +
  #geom_jitter(height = 0, width = 0.1, size =2, alpha=0.5) +
  theme_bw() +
  labs(y = "DMR hotspot size", x = NULL) +
  theme(axis.text.y = element_text(size = 10, colour = "black"),
        axis.text.x = element_blank(),
        axis.ticks.x = element_blank(),
        axis.title = element_text(size = 12, colour = "black", face = "bold"))

pSize <- ggplot(dmrSizeDF %>% filter(V1 != "chrX"), aes(x = DMR.size/1000000, colour = test)) +
  geom_density(size = 1) +
  theme_bw() +
  scale_colour_manual(values = c("grey", "#ef233c")) +
  labs(x = "DMR hotspot size (Mb)", colour = NULL) +
  xlim(0,30)+
  geom_vline(xintercept = median(dmrSizeDF$DMR.size[dmrSizeDF$test == "DMR_HPV"])/1000000, linetype = 2, colour = "#ef233c")+
  geom_vline(xintercept = median(dmrSizeDF$DMR.size[dmrSizeDF$test == "all"])/1000000, linetype = 2, colour = "grey")+
  theme(axis.text.x = element_text(size = 10, colour = "black"),
        axis.text.y = element_blank(),
        axis.ticks.y = element_blank(),
        panel.grid.minor.y = element_blank(), panel.grid.major.y = element_blank(),
        axis.title = element_text(size = 12, colour = "black", face = "bold")) 

summary(dmrSizeDF$DMR.size[dmrSizeDF$V1 != "chrX" & dmrSizeDF$test == "all"])
```

```
##     Min.  1st Qu.   Median     Mean  3rd Qu.     Max. 
##    18307  2620352  3903772  5166310  6234713 56516870
```

```
summary(dmrSizeDF$DMR.size[dmrSizeDF$V1 != "chrX" & dmrSizeDF$test == "DMR_HPV"])
```

```
##     Min.  1st Qu.   Median     Mean  3rd Qu.     Max. 
##  1851296  2923136  6080581  8639463 11619011 26009941
```

```
ggsave(plot = pSize, filename = "figure6/DMRHPVHotspotSize.pdf", width = 4, height = 5, units = "in")
pSize
```

#### DMR heatmap

```
## new code
alldmrs <- lapply(sigEvents, function(x){
  df <- x[[6]]
  return(df)
})
dmrs <- bind_rows(alldmrs, .id = "event.id")

dmrsFilt <- dmrs[abs(dmrs$hpvDistStart) < 10000000 & abs(dmrs$hpvDistEnd) < 10000000,]
dmrsFilt$region <- intHPV$region[match(dmrsFilt$event.id, intHPV$event.id)]
dmrsFilt <- dmrsFilt[dmrsFilt$event.id %in% m$id,]
dmrsFilt$event.id <- factor(dmrsFilt$event.id, levels = m$id)
intDF <- data.frame(event.id = unique(dmrsFilt$event.id), integer = as.integer(unique(dmrsFilt$event.id)))
dmrsFilt$num <- intDF$integer[match(dmrsFilt$event.id, intDF$event.id)]

pdmrs <- ggplot(dmrsFilt, aes(xmin = (hpvDistStart)/1000000, xmax = (hpvDistEnd)/1000000, ymin = num-1, ymax = num+1)) +
  geom_rect(size=0.01, colour = "black") +
  #geom_rect(aes(xmin = start, xmax = end, ymin = as.integer(as.factor(region))-1, ymax = as.integer(as.factor(region))+1, fill = "#ef233c")) +
  facet_wrap(event.id ~ ., scales = "free_y", ncol = 1) +
  theme_bw() +
  labs(x = "distance from HPV integration (Mb)")+
  geom_vline(xintercept = 0, linetype = 1, colour = "#ef233c", size = 0.5)+
  xlim(-10,10)+
  theme(strip.background = element_blank(),
        strip.text = element_blank(),
        axis.text.y = element_blank(),
        axis.text.x = element_text(size = 12, colour = "black"),
        axis.title.x = element_text(size = 14, colour = "black", face = "bold"),
        panel.grid.major = element_blank(),
        panel.grid.minor = element_blank(),
        axis.ticks.y = element_blank())

ggsave(plot = pdmrs, filename = "figure6/DMRsAroundHPV10Mb.pdf", width = 8, height = 10, units = "in")
pdmrs
```

#### Direction of methylation

```
### ----------------------------------------------------------
### MAKE THE FIGURE SHOWING DMR METHYLATION AROUND HPV
### ----------------------------------------------------------
v1 <- names(sigEvents)
v2 <- m$id
order_indices <- match(v2, v1)
sigEvents <- sigEvents[order_indices]
all(names(sigEvents) == v2)
```

```
## [1] TRUE
```

```
p <- NULL
for (i in 1:length(sigEvents)) {
  sub <- sigEvents[[i]]
  
  if(is.null(sub[[3]]) & is.null(sub[[4]])){
    plot <- ggplot() +
      geom_rect(data=sub[[1]], aes(xmin = hpvDistStart/1000, xmax = hpvDistEnd/1000, ymin = 0, ymax = 1, fill = diff.Methy.Dir, colour = diff.Methy.Dir), size = 0.5) +
      theme_bw() + 
      xlim(c(-500,500))+
      scale_fill_distiller(palette = "RdBu", limits = c(-1,1)) +
      scale_colour_distiller(palette = "RdBu", limits = c(-1,1)) +
      geom_vline(xintercept = 0, linetype = 2) +
      theme(panel.grid.minor = element_blank(),
            panel.grid.major = element_blank(),
            axis.text = element_blank(),
            axis.ticks = element_blank(),
            legend.position = "none",
            plot.margin = unit(c(0, 0, 0, 0), "cm"))
  } else if(is.null(sub[[3]])){
    plot <- ggplot() +
      geom_rect(data=sub[[1]], aes(xmin = hpvDistStart/1000, xmax = hpvDistEnd/1000, ymin = 0, ymax = 1, fill = diff.Methy.Dir, colour = diff.Methy.Dir), size = 0.5) +
      theme_bw() + 
      geom_rect(data=sub[[4]], aes(xmin=(start/1000), xmax = (end/1000),ymin = 0, ymax = 1), fill = "grey")+
      xlim(c(-500,500))+
      scale_fill_distiller(palette = "RdBu", limits = c(-1,1)) +
      scale_colour_distiller(palette = "RdBu", limits = c(-1,1)) +
      geom_vline(xintercept = 0, linetype = 2) +
      theme(panel.grid.minor = element_blank(),
            panel.grid.major = element_blank(),
            axis.text = element_blank(),
            axis.ticks = element_blank(),
            legend.position = "none",
            plot.margin = unit(c(0, 0, 0, 0), "cm"))
  } else if(is.null(sub[[4]])){
    plot <- ggplot() +
      geom_rect(data=sub[[1]], aes(xmin = hpvDistStart/1000, xmax = hpvDistEnd/1000, ymin = 0, ymax = 1, fill = diff.Methy.Dir, colour = diff.Methy.Dir), size = 0.5) +
      theme_bw() + 
      geom_rect(data=sub[[3]], aes(xmin=(start/1000), xmax = (end/1000),ymin = 0, ymax = 1), fill = "grey")+
      xlim(c(-500,500))+
      scale_fill_distiller(palette = "RdBu", limits = c(-1,1)) +
      scale_colour_distiller(palette = "RdBu", limits = c(-1,1)) +
      geom_vline(xintercept = 0, linetype = 2) +
      theme(panel.grid.minor = element_blank(),
            panel.grid.major = element_blank(),
            axis.text = element_blank(),
            axis.ticks = element_blank(),
            legend.position = "none",
            plot.margin = unit(c(0, 0, 0, 0), "cm"))
  } else{
    plot <- ggplot() +
      geom_rect(data=sub[[1]], aes(xmin = hpvDistStart/1000, xmax = hpvDistEnd/1000, ymin = 0, ymax = 1, fill = diff.Methy.Dir, colour = diff.Methy.Dir), size = 0.5) +
      theme_bw() + 
      geom_rect(data=sub[[3]], aes(xmin=(start/1000), xmax = (end/1000),ymin = 0, ymax = 1), fill = "grey")+
      geom_rect(data=sub[[4]], aes(xmin=(start/1000), xmax = (end/1000),ymin = 0, ymax = 1), fill = "grey")+
      xlim(c(-500,500))+
      scale_fill_distiller(palette = "RdBu", limits = c(-1,1)) +
      scale_colour_distiller(palette = "RdBu", limits = c(-1,1)) +
      geom_vline(xintercept = 0, linetype = 2) +
      theme(panel.grid.minor = element_blank(),
            panel.grid.major = element_blank(),
            axis.text = element_blank(),
            axis.ticks = element_blank(),
            legend.position = "none",
            plot.margin = unit(c(0, 0, 0, 0), "cm"))
  } 
  
  p[[i]] <- plot
}

pFinal <- plot_grid(plotlist = p, align = "v", ncol = 1)
ggsave(plot=pFinal, filename = "figure6/dmrMethylDirSigEvents.pdf", height = 10, width = 8, units = "in")
pFinal
```

#### DMR Density comparison

```
### ----------------------------------------------------------
### DMR DENSITY COMPARISONS
### ----------------------------------------------------------

files8 <- grep("/plottingDensityValues.txt", list.files("/path/to/htmcp/call_integration/output", 
                                           recursive = T, full.names = T),value = T)
files8_500kbup <- grep("dmr_permute_500kbup", files8,value = T)
files8_500kbdown <- grep("dmr_permute_500kbdown", files8,value = T)
names8_500kbup <- gsub("/path/to/htmcp/call_integration/output/|/event_phase|/dmr_permute_500kbup/plottingDensityValues.txt", "", files8_500kbup)
names8_500kbdown <- gsub("/path/to/htmcp/call_integration/output/|/event_phase|/dmr_permute_500kbdown/plottingDensityValues.txt", "", files8_500kbdown)

# Import data
methDensity_500kbup <- lapply(files8_500kbup, read.delim, header = TRUE, sep = "\t")
names(methDensity_500kbup) <- names8_500kbup
methDensity_500kbup <- dplyr::bind_rows(methDensity_500kbup, .id = "id")
methDensity_500kbup$region <- "upstream"

methDensity_500kbdown <- lapply(files8_500kbdown, read.delim, header = TRUE, sep = "\t")
names(methDensity_500kbdown) <- names8_500kbdown
methDensity_500kbdown <- dplyr::bind_rows(methDensity_500kbdown, .id = "id")
methDensity_500kbdown$region <- "downstream"

# put together
methDensity <- rbind(methDensity_500kbup, methDensity_500kbdown)

# add info
methDensity$id <- gsub("/", ":", methDensity$id)
methDensity$pvalue <- m$pval[match(methDensity$id, m$id)]
methDensity$number <- m$number[match(methDensity$id, m$id)]
methDensity$number <- factor(methDensity$number, levels = 1:nrow(m))

# plot
plotD <- ggplot(methDensity %>% filter(!is.na(number)), aes(x = region, y = dmr.density)) +
  geom_boxplot(outlier.shape = NA) +
  geom_jitter(aes(colour = sample), height = 0, width = 0.2, size =2, alpha=0.5) + 
  facet_wrap(vars(number), nrow = 5, scales = "free_y") +
  theme_minimal() +
  labs(x = NULL, y = "DMR Density") +
  #annotate("text", x=1.3, y=max(de$dmr.density[de$region == "hpv_region"]), 
  #         label= paste0("pvalue = ", p), 
  #         colour = "grey30", size = 5) + 
  scale_colour_manual(values = c("#e63946", "#1d3557")) +
  theme(panel.grid = element_blank(), 
        axis.text.y = element_text(size = 13),
        axis.text.x = element_text(size = 13),
        axis.title = element_text(size=14), 
        axis.ticks.y = element_line(),
        axis.line = element_line(), 
        legend.position = "none")
plotD
```

```
ggsave(plot=plotD, filename = "figure6/dmrDensityBoxplots.pdf", height = 10, width = 14, units = "in")
```

#### HP Methylation Significance

```
### ----------------------------------------------------------
### HP METHYLATION IN SIGNIFICANT EVENTS
### ----------------------------------------------------------

dmrs_sig <- dmrsFilt %>% filter(event.id %in% sig_event_ids)
dmrs_sig <- dmrs_sig %>% filter(abs(hpvDistStart) < 5000000)
dmrs_sig <- melt(dmrs_sig, id.vars = c("event.id","hpvDistStart"), measure.vars = c("meanMethy1", "meanMethy2"), variable.name = "haplotype", value.name = "meanMethyl")
dmrs_sig <- dmrs_sig %>%
    group_by(event.id,hpvDistStart) %>%
    summarise(HP_low = min(meanMethyl), HP_high = max(meanMethyl)) %>%
    pivot_longer(cols = c(HP_low, HP_high),names_to = "HP_type", values_to = "HP_value")
dmrs_sig$significant_region <- m$significant_region[match(dmrs_sig$event.id, m$id)]
dmrs_sig$significant_region <- gsub("one_direction_down|one_direction_up", "one_direction",
                                    dmrs_sig$significant_region)
#dmrs_sig <- dmrs_sig %>% filter(event.id == "HTMCP-03-06-02411:event9_chr9_26300536")

plotE <- ggplot(dmrs_sig, aes(x = hpvDistStart/1000000, y = HP_value, colour = HP_type)) +
    geom_vline(xintercept = 0, size = 1)+
    geom_point(size = 1, alpha = 0.3) +
    facet_grid(event.id ~ significant_region, drop = T) +
    theme_bw() +
    labs(x = "distance from HPV (Mb)", y = "% methylated", colour = "haplotype")+
    scale_colour_manual(values=RColorBrewer::brewer.pal(11,"RdBu")[c(2,10)]) +
    theme(panel.grid.major.y = element_blank(),
          panel.grid.minor.y = element_blank(),
          axis.title = element_text(size = 13, face = "bold", colour = "black"),
          axis.text = element_text(size = 12, colour = "black"))
ggsave(filename = "figure6/sig_events_HP_spread.pdf", plotE,
       height = 6, width = 6)
plotE
```

#### Save table

```
### ----------------------------------------------------------
### SAVE TABLES
### ----------------------------------------------------------
# integration event characteristics
write.table(m, file = "tables/integrationEventsOnDMRHotspots.txt", quote = F, col.names = T, row.names = F, sep = "\t")

kable(head(m))
```

| id | aveMethyl | status | int.type | event | sample | HPV.type | DMR.size | is.event.transcribed | event.loci | nevents | pval\_up | pval\_down | significant\_region | significant\_region\_simple | number |
| --- | --- | --- | --- | --- | --- | --- | --- | --- | --- | --- | --- | --- | --- | --- | --- |
| HTMCP-03-06-02411:event9\_chr9\_26300536 | -0.5190947 | unmethylated | dup-like | event9 | HTMCP-03-06-02411 | HPV16 | 8244162 | no | HTMCP-03-06-02411:event9 | 9 | 0.082 | 0.042 | one\_direction\_down | one\_direction | 1 |
| HTMCP-03-06-02427:event2\_chr2\_158296508 | -0.4486955 | unmethylated | unmatched | event2 | HTMCP-03-06-02427 | HPV18 | 2841151 | no | HTMCP-03-06-02427:event2 | 2 | 0.002 | 0.062 | one\_direction\_up | one\_direction | 2 |
| HTMCP-03-06-02213:event1\_chr11\_100777194 | -0.3625243 | unmethylated | unmatched | event1 | HTMCP-03-06-02213 | HPV16 | 3980674 | no | HTMCP-03-06-02213:event1 | 4 | 0.857 | 0.008 | one\_direction\_down | one\_direction | 3 |
| HTMCP-03-06-02109:event3\_chr3\_119178473 | -0.7115699 | unmethylated | multi-breakpoint | event3 | HTMCP-03-06-02109 | HPV16 | 13243244 | yes | HTMCP-03-06-02109:event3 | 6 | 0.257 | 0.120 | none | none | 4 |
| HTMCP-03-06-02411:event5\_chr4\_19434144 | -0.2819767 | unmethylated | multi-breakpoint | event5 | HTMCP-03-06-02411 | HPV16 | 26009941 | no | HTMCP-03-06-02411:event5 | 9 | 0.328 | 0.829 | none | none | 5 |
| HTMCP-03-06-02411:event1\_chr13\_45223518 | -0.2461333 | variable | multi-breakpoint | event1 | HTMCP-03-06-02411 | HPV16 | 2077866 | no | HTMCP-03-06-02411:event1 | 9 | 0.083 | 0.083 | none | none | 6 |

## The significance of DMR density at HPV int events

```
densityFilesHPV <- grep("hpvDensity", list.files("/path/to/htmcp/call_integration/dmr_density", 
                                           recursive = T, full.names = T),value = T)
densityFilesCtrl <- grep("shuffleDensity", list.files("/path/to/htmcp/call_integration/dmr_density", 
                                           recursive = T, full.names = T),value = T)
window <- gsub("/path/to/htmcp/call_integration/dmr_density/hpvDensity|.txt", "", densityFilesHPV)

# Import data
densityHPVList <- lapply(densityFilesHPV, read.delim, header = TRUE, sep = "\t")
names(densityHPVList) <- window
densityCtrlList <- lapply(densityFilesCtrl, read.delim, header = TRUE, sep = "\t")
names(densityCtrlList) <- window

densityHPV <- dplyr::bind_rows(densityHPVList, .id = "window")
densityHPV$test <- "HPV"
densityCtrl <- dplyr::bind_rows(densityCtrlList, .id = "window")
densityCtrl$test <- "control"

# put dataframes together
density <- rbind(densityHPV, densityCtrl)
density <- density[density$window != "10000000",]

# factor the windows smallest to largest
options(scipen=999) # turn off scientific notation
density$window <- factor(density$window, levels = as.character(sort(as.numeric(unique(density$window)))))

# get the p values
p_values <- density %>%
  group_by(window) %>%
  summarise(p_value = wilcox.test(density ~ test)$p.value) %>%
  mutate(padj = p.adjust(p_value, method = "BH")) %>%
  mutate(log10padj = -log10(padj))

# make figure showing the DMR densities at different window sizes
pdens <- ggplot(density, aes(x = test, y = density)) +
    #geom_boxplot(outlier.shape = NA) + 
    #geom_jitter(height = 0, width = 0.2, size =0.5, alpha=0.1) +
    geom_violin()+
    theme_bw() +
    facet_grid(~ window)+
    #scale_fill_manual(values = c("#8d99ae", "#fb8500"))+
    #ggtitle("genes 1-200kb from HPV integration events")+
    xlab("region") + 
    ylab("DMR density") + 
    theme(panel.grid.minor = element_blank(), 
          axis.text = element_text(size = 13, colour = "black"),
          axis.title = element_text(size=14, face = "bold"), 
          axis.ticks.y = element_line(),
          axis.line = element_line(), 
          legend.position = "none") +
    geom_text(data = p_values, aes(x = 1.5, y = max(density$density), label = paste("padj =", signif(padj, 2))), size = 3)
ggsave(plot=pdens, filename = "figure6/testAllDMRsDistance.pdf", height = 5, width = 12, units = "in")
pdens
```

```
# make a figure showing the p values at the window sizes
p_values$start <- 5000000 - as.numeric(as.character(p_values$window))/2
p_values$end <- 5000000 + as.numeric(as.character(p_values$window))/2

ppval <- ggplot(p_values, aes(ymin = as.integer(window), ymax = as.integer(window)+0.8, xmin = start, xmax = end, fill = log10padj)) +
    geom_rect() + 
    theme_minimal() +
    scale_fill_distiller(palette = "PuBuGn", direction = 1)+
    theme(panel.grid.minor = element_blank(), 
          panel.grid.major = element_blank(), 
          axis.text = element_text(size = 13, colour = "black"),
          axis.title = element_text(size=14, face = "bold"), 
          axis.ticks.y = element_line(),
          axis.line = element_line())
ggsave(plot=ppval, filename = "figure6/pvalueDMRDistance.pdf", height = 5, width = 7, units = "in")
ppval
```

#### GC Content Testing

```
# Calculate the mean GC content for each window
mean_gc <- density %>%
  group_by(window, test) %>%
  summarize(mean_gc = mean(gc_content, na.rm = TRUE))

# make a distribution plot
pGcHpv <- ggplot(density, aes(x = gc_content, colour = window, fill = window)) +
    geom_density(alpha = 0.5, bw = 2) +
    facet_grid(test ~ window) +
    theme_bw() +
    xlab("GC content") + 
    xlim(20,65)+
    #geom_vline(data = mean_gc, aes(xintercept = mean_gc), linetype = 2, size = 0.5) +
    theme(panel.grid.minor = element_blank(), 
          axis.text = element_text(size = 13, colour = "black"),
          axis.title = element_text(size=14, face = "bold"), 
          axis.ticks.y = element_line(),
          axis.line = element_line(),
          legend.position = "none") 
ggsave(plot=pGcHpv, filename = "figure6/gcContentCtrlHPV.pdf", height = 5, width = 7, units = "in")
pGcHpv
```

## Expression of nearby genes

#### ASE At NR4A1 and NR4A3

```
testgene <- "NR4A1"
test_sample <- allgenes$sample[allgenes$gene.id == testgene]

p1_ase <- ase %>%
    filter(gene == testgene) %>%
    mutate(colour = ifelse(sample %in% test_sample, "test", "others")) %>%
    ggplot(aes(x = gene, y = majorAlleleFrequency)) +
    geom_boxplot(outlier.shape = NA) +
    geom_jitter(aes(fill = colour, colour = aseResults), height = 0, width = 0.2, size =4, shape = 21, stroke = 1, alpha = 0.8) +
    scale_colour_manual(values = c("black", "grey"), na.value = "white")+
    labs(x = NULL, y = "RNA major allele frequency") +
    scale_fill_manual(values = c("grey", "dark red")) +
    theme_minimal() +
    theme(panel.grid = element_blank(), 
          axis.text = element_text(size = 13, colour = "black"),
          axis.title = element_text(size=14, colour = "black"), 
          axis.ticks.y = element_line(),
          axis.line = element_line(), 
          legend.position = "none") 
ggsave(plot = p1_ase, filename = paste0("figure6/",testgene, "_MAF_ASE.pdf"), width = 2.7, height = 2.7)
p1_ase
```

```
# find the recurring genes
t <- table(outliergenes$gene.id)
upgenes_reocurring <-  t[t > 1]
upgenes_reocurring <- upgenes_reocurring[order(upgenes_reocurring, decreasing = T)]
upgenes_reocurring
```

```
## MIEN1 
##     2
```

```
### -------------------------------------------------------------------------------
### ADJUST THE PVALUES AROUND THE EVENT IN EACH SAMPLE
### -------------------------------------------------------------------------------
genehtmcpPFilt$log2FC.L <- "> 3"
genehtmcpPFilt$log2FC.L[genehtmcpPFilt$log2FC <= 3] <- NA

labs <- outliergenes[outliergenes$log2FC > 2 | abs(outliergenes$log2FC) > 1 & outliergenes$ase_result == "ASE",]
labs <- labs[complete.cases(labs[,1:3]),]
labs <- labs %>% filter(abs(distance) < 1000000)

p_outliers <- outliergenes %>%
    filter(abs(distance) < 1000000) %>%
    ggplot(aes(x=distance,y=event.loci,fill=log2FC, colour = ase_result)) +
    geom_hline(data=outliergenes %>% filter(abs(distance) < 1000000 & is.event.transcribed =="yes"), aes(yintercept = event.loci), colour = "grey", size = 2)+
    geom_point(size=3, shape = 21, stroke = 1) +
    scale_color_manual(values = list(ASE="black", BAE="grey", no_data="white"))+
    scale_x_continuous(minor_breaks = seq(-1000000, 1000000, 100000), breaks = seq(-1000000, 1000000, 200000), limits = c(-1000000,1000000)) +
    geom_text_repel(data=labs, 
                    aes(x=distance,y=event.loci, label = gene.id), max.overlaps = 20, colour = "black", size = 3, fontface = "italic") +
    facet_grid(integration.type ~ ., scales = "free_y", space = "free_y") +
    scale_fill_distiller(palette = "RdBu",, limits = c(-5,5)) + 
    theme_bw() +
    theme(axis.text.y = element_blank(),
          axis.ticks.y = element_blank(),
          axis.text = element_text(size = 13, colour = "black"),
          axis.title = element_text(size=14, colour = "black", face = "bold"))
ggsave(plot = p_outliers, filename = "figure6/outlier_genes_ase_expression.pdf", width = 6, height = 6)
p_outliers
```

```
# make a figure for each bin

outliergenes$log2FC <- log2(outliergenes$sample.expr/outliergenes$median.expr)

p_outlier_dist <- ggplot(outliergenes, aes(x =abs(distance), y = log2FC, colour = ase_result)) +
    geom_point(size = 3, alpha = 0.75) +
    theme_bw() +
    labs(x="distance from HPV integration (kb)", colour="ASE result")+
    scale_x_continuous(minor_breaks = seq(0, 1000000, 100000), breaks = seq(0, 1000000, 200000)) +
    scale_color_manual(values = list(ASE="#d81159", BAE="#218380", no_data="#d3d3d3")) +
    theme(axis.text = element_text(colour = "black", size = 12),
          axis.title = element_text(colour = "black", size = 14, face = "bold"),
          legend.text = element_text(size = 12, colour = "black"),
          legend.title = element_text(colour = "black", size = 14, face = "bold"))
ggsave(plot = p_outlier_dist, filename = "figure6/outlier_genes_distance.pdf", width = 6, height = 2.5)

### -------------------------------------------------------------------------------
### SAVE TABLES
### -------------------------------------------------------------------------------

write.table(outliergenes, file = "tables/outlierGeneExpression.txt", quote = F, col.names = T, row.names = F, sep = "\t")

p_outlier_dist
```

#### Gene expression

```
### -------------------------------------------------------------------------------
### DETERMINE FC DIFFERENCE IN EXPRESSED VS NON-EXPRESSED EVENTS 
### -------------------------------------------------------------------------------

give.n <- function(x){
    return(c(y = median(x)*1.05, label = length(x))) 
    # experiment with the multiplier to find the perfect position
}

# genes that are overlapping events

p0 <- allgenes %>%
    #filter(grepl("HTMCP", sample)) %>%
    filter(bin =="0") %>%
    ggplot(aes(y = log2FC, x = is.event.transcribed, fill = is.event.transcribed)) +
    geom_boxplot(outlier.shape = NA) + 
    geom_jitter(height = 0, width = 0.2, size =2, alpha=0.5) +
    theme_minimal() +
    scale_fill_manual(values = c("#8d99ae", "#fb8500"))+
    #ggtitle("genes overlapping HPV integration events")+
    xlab("HPV fusion transcript detected?") + 
    stat_summary(fun.data = give.n, geom = "text", fun = median, 
                 position = position_dodge(width = 0.75)) +
    theme(panel.grid.minor = element_blank(), 
          axis.text = element_text(size = 13, colour = "black"),
          axis.title = element_text(size=14, face = "bold"), 
          axis.ticks.y = element_line(),
          axis.line = element_line(), 
          legend.position = "none") +
    stat_compare_means(method = "wilcox.test")

p200 <- allgenes %>%
    #filter(grepl("HTMCP", sample)) %>%
    filter(bin %in% c("1-100000", "100000-200000")) %>%
    ggplot(aes(y = log2FC, x = is.event.transcribed, fill = is.event.transcribed)) +
    geom_boxplot(outlier.shape = NA) + 
    geom_jitter(height = 0, width = 0.2, size =2, alpha=0.5) +
    theme_minimal() +
    scale_fill_manual(values = c("#8d99ae", "#fb8500"))+
    #ggtitle("genes 1-200kb from HPV integration events")+
    xlab("HPV fusion transcript detected?") + 
    stat_summary(fun.data = give.n, geom = "text", fun = median, 
                 position = position_dodge(width = 0.75)) +
    theme(panel.grid.minor = element_blank(), 
          axis.text = element_text(size = 13, colour = "black"),
          axis.title = element_text(size=14, face = "bold"), 
          axis.ticks.y = element_line(),
          axis.line = element_line(), 
          legend.position = "none") +
    stat_compare_means(method = "wilcox.test")

p500 <- allgenes %>%
    #filter(grepl("HTMCP", sample)) %>%
    filter(bin %in% c("200000-300000", "300000-400000","400000-500000")) %>%
    ggplot(aes(y = log2FC, x = is.event.transcribed, fill = is.event.transcribed)) +
    geom_boxplot(outlier.shape = NA) + 
    geom_jitter(height = 0, width = 0.2, size =2, alpha=0.5) +
    theme_minimal() +
    scale_fill_manual(values = c("#8d99ae", "#fb8500"))+
    #ggtitle("genes 1-200kb from HPV integration events")+
    xlab("HPV fusion transcript detected?") + 
    stat_summary(fun.data = give.n, geom = "text", fun = median, 
                 position = position_dodge(width = 0.75)) +
    theme(panel.grid.minor = element_blank(), 
          axis.text = element_text(size = 13, colour = "black"),
          axis.title = element_text(size=14, face = "bold"), 
          axis.ticks.y = element_line(),
          axis.line = element_line(), 
          legend.position = "none") +
    stat_compare_means(method = "wilcox.test")

p_genes <- plot_grid(p0,p200,p500, align = "h",nrow = 1)
ggsave(plot=p_genes, filename = "figure6/log2FCGenesTranscribed.pdf", height = 3, width = 8, units = "in")
p_genes
```

```
genes <- outlier200$gene.id
test_samples <- gsub("-", ".", outlier200$sample)
plist <- NULL

for (i in 1:length(genes)){
  p2 <- expr_mat %>%
    filter(gene.id == genes[i]) %>%
    gather(sample, tpm,-gene.id) %>%
    mutate(colour = ifelse(sample == test_samples[i], "test", "others")) %>%
    ggplot(aes(x = gene.id, y = log10(tpm))) +
    geom_boxplot(outlier.shape = NA) +
    geom_jitter(aes(colour = colour), height = 0, width = 0.2, size =3, alpha=0.5) +
    labs(x = NULL, y = "log10(TPM)") +
    scale_colour_manual(values = c("grey", "dark red")) +
    theme_minimal() +
    theme(panel.grid = element_blank(), 
          axis.text = element_text(size = 13),
          axis.title = element_text(size=14), 
          axis.ticks.y = element_line(),
          axis.line = element_line(), 
          legend.position = "none") 
  plist[[i]] <- p2
}

pgrid <- plot_grid(plotlist=plist, nrow = 10)
ggsave(plot = pgrid, filename = "figure6/outlierGeneExpressionBoxplot.pdf", width = 12, height = 15)
pgrid
```
